# Significant East Asian Affinity of the Sichuan Hui Genomic Structure Suggests the Predominance of the Cultural Diffusion Model in the Genetic Formation Process

**DOI:** 10.3389/fgene.2021.626710

**Published:** 2021-06-14

**Authors:** Yan Liu, Junbao Yang, Yingxiang Li, Renkuan Tang, Didi Yuan, Yicheng Wang, Peixin Wang, Shudan Deng, Simei Zeng, Hongliang Li, Gang Chen, Xing Zou, Mengge Wang, Guanglin He

**Affiliations:** ^1^School of Basic Medical Sciences, North Sichuan Medical College, Nanchong, China; ^2^AnLan AI, Shenzhen, China; ^3^Department of Forensic Medicine, College of Basic Medicine, Chongqing Medical University, Chongqing, China; ^4^College of Medical Information, Chongqing Medical University, Chongqing, China; ^5^School of Medical Imaging, North Sichuan Medical College, Nanchong, China; ^6^Hunan Key Lab of Bioinformatics, School of Computer Science and Engineering, Central South University, Changsha, China; ^7^Department of Forensic Genetics, Institute of Forensic Medicine, West China School of Basic Science and Forensic Medicine, Sichuan University, Chengdu, China; ^8^Department of Anthropology and Ethnology, Institute of Anthropology, National Institute for Data Science in Health and Medicine, School of Life Sciences, Xiamen University, Xiamen, China

**Keywords:** Hui, cultural diffusion, genetic admixture, ancestral origin, genetic formation

## Abstract

The ancestral origin and genomic history of Chinese Hui people remain to be explored due to the paucity of genome-wide data. Some evidence argues that an eastward migration of Central Asians gave rise to modern Hui people, which is referred to as the *demic diffusion hypothesis*; other evidence favors the *cultural diffusion hypothesis*, which posits that East Asians adopted Muslim culture to form the modern culturally distinct populations. However, the extent to which the observed genetic structure of the Huis was mediated by the movement of people or the assimilation of Muslim culture also remains highly contentious. Analyses of over 700 K SNPs in 109 western Chinese individuals (49 Sichuan Huis and 60 geographically close Nanchong Hans) together with the available ancient and modern Eurasian sequences allowed us to fully explore the genomic makeup and origin of Hui and neighboring Han populations. The results from PCA, ADMIXTURE, and allele-sharing-based *f*-statistics revealed a strong genomic affinity between Sichuan Huis and Neolithic-to-modern Northern East Asians, which suggested a massive gene influx from East Asians into the Sichuan Hui people. Three-way admixture models in the *qpWave/qpAdm* analyses further revealed a small stream of gene influx from western Eurasians into the Sichuan Hui people, which was further directly confirmed via the admixture event from the temporally distinct Western sources to Sichuan Hui people in the *qpGraph*-based phylogenetic model, suggesting the key role of the cultural diffusion model in the genetic formation of the Sichuan Huis. ALDER-based admixture date estimation showed that this observed western Eurasian admixture signal was introduced into the Sichuan Huis during the historic periods, which was concordant with the extensive western–eastern communication along the Silk Road and historically documented Huis' migration history. In summary, although significant cultural differentiation exists between Hui people and their neighbors, our genomic analysis showed their strong genetic affinity with modern and ancient Northern East Asians. Our results support the hypothesis that the Sichuan Huis arose from a mixture of minor western Eurasian ancestry and predominant East Asian ancestry.

## Introduction

Archeologically and historically supported transcontinental exchange in North China and Northwest China during the past 5,000 years played an important role in the formation of the modern genetic, linguistic, and cultural diversity in East Asian people (Dong et al., 2020). The archeological record showed that wheat and barley agriculture and plant and animal domestication technologies spread from the Fertile Crescent into North China (Leipe et al., 2019; Dong et al., 2020). In addition, Northern Chinese millet farming technology was disseminated into western Eurasia (Leipe et al., 2019; Dong et al., 2020). Zoo-archeological/phytoarcheological findings and the existence of signature of western Bronze Age technologies (metallurgy, chariots, horses, cattle, and sheep/goats) in China suggested a massive introduction of western cultural elements into China in response to the transformation of human subsistence patterns or climate changes (Leipe et al., 2019). Late Paleocene and early Holocene genomes from Siberia have revealed several genetically diverse Paleolithic lineages (Ancient Northern Eurasian Mal'ta1 lineage, Ancient North Siberian Yana lineage, etc.) and a typical West-to-East genetic cline from the Caucasus region to the Russian Far East (Raghavan et al., 2015; Damgaard et al., 2018; De Barros Damgaard et al., 2018; Sikora et al., 2019). An ancient DNA study of 101 Bronze Age Eurasian humans found that large-scale population migration, admixture, and replacement have reshaped the modern Eurasian demographic structure, including the eastward spread of Yamnaya/Afanasievo populations into Central Asia, the Xinjiang Tianshan mountain region in Northwest China, and the Altai-Sayan region (Allentoft et al., 2015; Ning et al., 2019). Eastward-migrating steppe pastoralists and their descendants gradually admixed with or were replaced by local indigenous steppe nomads and formed the multioriginated Scythian pastoralist tribes (Damgaard et al., 2018), as well as the later-formed Xiongnu/Xianbei/Rouran/Uyghur/Turkic/Mongolic confederations. These eastern Eurasian nomadic pastoralist empires became the dominant groups, and their subsequent westward migrations reshaped the genetic landscape of populations from the Eurasian steppe once again (Damgaard et al., 2018; Jeong et al., 2020). Modern Eurasian population genomic history has also documented large-scale western–eastern Eurasian admixtures and massive population movement (Yunusbayev et al., 2015). In terms of language diversity, modern populations belonging to Indo-European, Uralic, Tungusic, Mongolic, and Turkic families/groups have been widely distributed in Eurasia (Wang and Robbeets, 2020). From a cultural perspective, significantly different steppe-pastoralist-associated cultures replaced or mixed with the incoming contemporaneous cultures, as exemplified by the cultural evolutionary sequence of the Yamnaya, Afanasievo, Sintashta, Andronovo, and other historic cultures (Allentoft et al., 2015; Damgaard et al., 2018; Damgaard et al., 2018; De Barros Damgaard et al., 2018). However, the extent to which these genetically/historically/archeologically supported shifts in culture, genetics, and language have shaped the genetic landscape of modern Chinese populations remains to be explored.

Recent ancient and modern genomes from China have provided some important new insights into the genetic development of modern East Asians (Wang M. et al., 2020). Yang et al. reported 26 Neolithic-to-historic genomes from Shandong, Fujian, and the surrounding regions and found that modern DNA-supported North-to-South genetic differentiation has existed in East Asia since the early Neolithic period. North–South bidirectional migration and coastal continental East Asian population movement have shaped the observed genetic variations among East Asians (Yang et al., 2020). Wang et al. reported one large-scale ancient DNA study focused on ancient human remains from South Siberia and Mongolia in the north to Taiwan in the south, which documented culturally/genetically strong western–eastern communication in the contact region of the eastern Mongolian steppe and reported three large Holocene population migrations from Northeast China, the Yellow River basin, and the Yangtze River basin. These expansion events were partially or completely associated with the spread of the major language families existing in East Asia (Wang C. C. et al., 2020). Ning et al. sequenced genomes from northern Chinese Neolithic-to-Iron-Age populations and documented the strong association between subsistence strategy changes and population transformation (Ning et al., 2020). Interestingly, these ancient genomic analyses focused on the Yellow River basin or South China did not reveal any western Eurasian admixture signatures (Ning et al., 2020; Wang C. C. et al., 2020; Yang et al., 2020). However, ancient genomes of Shirenzigou Iron Age people in Northwest China have documented western Yamnaya ancestry (Ning et al., 2019). The potential influence of western Eurasian ancestry in modern northwestern Chinese populations, which is relevant to molecular anthropology, medical genetics, and precision medicine, remains unknown.

The development and prosperity of commercial and trade exchanges on the historically documented Silk Road further facilitated recent eastern-to-western communication; however, the extent to which this cultural exchange or prehistoric cultural transformation in Northwest China was accompanied by population migration or only the adoption of ideas needed to be formally tested. The formation of the Hui people and the spread of Muslim culture are one of the most important cases for exploring and testing these two opposing hypotheses. The *demic diffusion model* states that all modern East Asian Muslim culture originated from western Eurasia and spread into China with substantial external gene flow. In contrast, the *cultural diffusion model* states that the eastward dissemination of Muslim culture was not accompanied by large-scale population movement. Scholars have conducted several population genetic surveys based on STRs, indels, and other lower-density genetic markers and found that the modern Chinese Hui originated from East Asia. However, others held the opposite opinion that East Asian Hui people descended from western Eurasian migrants (Wang et al., 2019). Zhou et al. (2020) genotyped 30 InDel loci in 129 Ningxia Hui individuals and found that East Asian populations contributed more genetic variants to Hui people than western Eurasian populations, which was consistent with Zou's observation in the Wuzhong Hui population (Zou et al., 2020). He et al. (2018) sequenced 165 ancestry informative single-nucleotide polymorphisms (AISNPs) in 159 Hui people and found East Asian-dominant ancestry in Hui along the Silk Road, which was in accordance with the autosomal STR-based results (Yao et al., 2016). However, the Y-SNP-based population genetic survey also identified western Eurasian founding lineages in East Asian Hui (Wang et al., 2019). Thus, a whole-genome-based genetic investigation needs to be conducted to explore the origin, diversification, and subsequent genetic admixture of modern East Asian Hui people.

Sichuan, located in the interior of southwestern China, is one of China's 23 provinces. This region is bounded by 26°03′–34°19′ north latitude and 97°21′–108°12′ east longitude and neighbors Shaanxi, Gansu, and Qinghai in the north; Tibet in the west; and Yunnan and Guizhou in the south and Chongqing in the east. Its most recent census population size was over 91.29 million, and all officially recognized Chinese ethnic populations have lived here, including the Hui people. The Hui population has a size of over 10.86 million in China. Hui people are distributed in many areas of northwestern, southern, northern and eastern Sichuan, with a population size of over 0.11 million. Here, we obtained high-density SNP data from 49 Sichuan Hui people and 60 Han individuals from neighboring areas and made one of the most comprehensive population comparisons to date based on the co-analysis of genome-wide data of all available modern and ancient Eurasian reference populations (Jeong et al., 2020; Ning et al., 2020; Wang C. C. et al., 2020; Yang et al., 2020). We aimed to address the following two questions: (I) the extent of genetic heterogeneity or homogeneity among geographically different Hui people or between Huis and their adjacent neighbors and (II) the timing, admixture sources, and origin of modern Huis and the models (cultural diffusion vs. demic diffusion) that played a key role in the establishment of the modern Sichuan Hui population.

## Materials and Methods

### Sample Collection and DNA Preparation

We collected saliva samples from 60 Nanchong Han and 49 Boshu Hui individuals in Sichuan Province, Southwest China (Supplementary Figure 1). Each included individual was an indigenous person with at least three generations of history in the area and the offspring of a non-consanguineous marriage within the Hui or Han populations. We collected all samples with written informed consent and provided genetic testing results focused on their ancestral composition and genetic health status. This project was inspected and approved by the Medical Ethics Committee of North Sichuan Medical College. Our study protocol also followed the recommendations of the Helsinki Declaration of 2000 (World Medical Association, 2001). Genomic DNA was isolated via the PureLink Genomic DNA Mini Kit (Thermo Fisher Scientific) and preserved at −4°C until the next amplification.

### Genotyping, Quality Control, Data Preparation, and Working Datasets

We used the Infinium® Global Screening Array (GSA) to genotype ~6,992,479 SNPs from the autosomes (645,199), the Y chromosome (26,341), the X chromosome (22,512), and the mitochondrial genome (4,198). Genotype calling was carried out with the default parameters. PLINK 1.9 (Chang et al., 2015) was used to filter out the raw genotype data, and only the markers with missing site rates per person or missing rates per SNP below 0.01 were remained (mind: 0.01 and geno: 0.01). Generally, the final dataset kept the autosomal SNPs and samples consistent with the following criteria: (I) initial dataset only retained samples with a genotyping success rate >0.99, (II) the *p*-value of the Hardy–Weinberg exact test was larger than 0.001 (--hwe 0.001), and III) SNPs with a minor allele frequency of >0.01 (--maf 0.01) and a genotyping success rate per SNP higher than 0.01. We merged our 109 newly generated genomes with previously published modern and ancient population data included in the Human Origins dataset (lower-density dataset with more modern reference populations, including 72,531 overlapping SNPs, Supplementary Tables 1, 2) and 1,240 K dataset (higher-density dataset with 193,838 overlapping SNPs, Supplementary Table 3) from the Reich Lab (https://reich.hms.harvard.edu/allen-ancient-dna-resource-aadr-downloadable-genotypes-present-day-and-ancient-dna-data) or recent publications (Ning et al., 2020; Wang C. C. et al., 2020; Yang et al., 2020). Genome-wide data from a geographically distinct Hui group from Guizhou were also included in our formal admixture analysis (Wang Q. et al., 2020).

### Principal Component Analysis

We carried out principal component analysis (PCA) using the smartpca package built-in EIGENSOFT (Patterson et al., 2012) with the default parameters based on the LD-pruned and MAF-filtered dataset. We also added two additional constraints (numoutlieriter: 0 and lsqproject: YES) to project ancient populations onto the PCA background. We performed two sets of PCAs based on different datasets: one East Asian-based PCA focused on 1,370 individuals in 159 populations, and one Southern East Asian-based PCA focused on 963 individuals in 107 populations.

### Pairwise Fst Genetic Distances

We calculated the Fst genetic distance matrix (Weir and Cockerham, 1984) using our in-house script and PLINK 1.9 (Chang et al., 2015) to explore the genetic similarities and differences between Boshu Hui, Nanchong Han, and other modern and ancient Eurasian populations.

### ADMIXTURE Clustering

Using PLINK 1.9 (Chang et al., 2015), we removed the strongly linked SNPs with R-values larger than 0.4. We used 200 SNPs as the window width and 25 SNPs as the sliding window with the following parameters (--indep-pairwise 200 25 0.4). We ran model-based genetic clustering using ADMIXTURE 1.3.0 (Alexander et al., 2009) in unsupervised mode. We used predefined ancestral populations ranging from 2 to 20 (*K*: 2–20) with 10 replicates and found *K* = 13 as the best resolution for ancestry dissection (Supplementary Figure 2).

### Allele-Based Shared Ancestry Estimation

We used the *qp3pop* packages in ADMIXTOOLS (Patterson et al., 2012) to perform the *outgroup-f*_3_*(Source1, Source2; Mbuti)* to evaluate the shared genetic drift among 366 Eurasian modern and ancient populations using the default parameters. Next, we used the *qp3pop* package (Patterson et al., 2012) to perform *admixture-f*_3_*(Source1, Source2; Boshu Hui/Nanchong Han)* to explore the admixture signatures with different Eurasian ancestral source candidates, where significant negative-*f*_3_ values with Z-scores < −3 denoted that the target population was a result of admixture between two parental populations designated as source1 and source2. We finally used the *qpDstat* package (Patterson et al., 2012) to estimate the *f*_4_*-statistic* value with one additional parameter (*f*_4_Mode: YES), and the standard error was estimated using the block jackknife (Patterson et al., 2012).

### Phylogenetic Relationship Reconstructions

We used the neighbor-joining (NJ) algorithm to reconstruct phylogenetic relationships based on different genetic distance matrixes using MEGA 7.0 (Kumar et al., 2016) or TreeMix (Pickrell and Pritchard, 2012). We first built one NJ tree based on the inverted *f*_3_-based genetic distances (1/*outgroup-f*_3_) among 366 Eurasian populations to explore the genetic relationships among modern and ancient populations and newly focused populations and other reference groups. We ran TreeMix (Pickrell and Pritchard, 2012) with the default settings and 100 replicates to choose the best-fitted model and explore the phylogenetic relationships among 48 modern populations with migration events ranging from 0 to 11.

### Modeling Admixture History

We ran *qpWave/qpAdm* packages in ADMIXTOOLS (Patterson et al., 2012) to explore the minimum ancestral population number via rank tests and then evaluated the corresponding ancestral admixture proportion. We used one additional parameter, “allsnps: YES,” and nine right reference outgroup populations were used (Mbuti, Ust_Ishim, Kostenki14, Papuan, Australian, Mixe, MA1, Onge, Atayal).

### Deep Population History Reconstruction

We used *qpGraph* (Patterson et al., 2012) to successfully fit one admixture graph model to explore the observed genetic variations in the Han and Hui populations. The following parameters were used: blgsize: 0.05; lsqmode: NO; diag: 0.0001; hires: YES; initmix: 1,000; precision: 0.0001; zthresh: 0; terse: NO; useallsnps: NO. We followed the basic model to reconstruct the population genomic history of our targeted Hui and Han populations (Wang M. et al., 2020). The skeletal framework from our previously reported model was used (Wang M. et al., 2020), but we replaced the proxy of the northern Yellow River farmer with the geographically closer millet farming population.

### Method for Dating Admixture Events

We used Admixture-induced Linkage Disequilibrium for Evolutionary Relationships (ALDER) (Loh et al., 2013) to estimate the date scale of population admixture events for two Hui (Boshu and Guizhou) and Nanchong Han populations based on two datasets and the default parameters. First, we employed 28 potential ancestral candidates (25 eastern Eurasian-like sources: Htin_Mal, Kinh_Vietnam, Mlabri, Ami, Atayal, Dao, Hmong, PaThen, Mongol_Uuld, Daur_HGDP, Han_Shandong, CoLao, Dai_HGDP, Gelao_Longlin, LaChi, Li_Hainan, Maonan_Huanjiang, Zhuang_Guangxi, Tibetan_Lhasa, Tibetan_Shigatse, Xijia_Kaili, Dongjia_Kaili, Gejia_Kaili, Ulchi, Nganasan; three western Eurasian-like sources: French, Greek, Basque) included in the merged Human Origins dataset to date the admixture times of Han and Hui. We also used the Nanchong Han as East Asian source and other western Eurasians to date the formation of the Boshu Hui people. Second, we used 14 populations (Dai, Altaian, French, Mongolian, Oroqen, Tajik, Ami, Sardinian, Greek, Atayal, English, Pathan, Basque, Han_Nanchong) that included the merged 1,240 K dataset to date the admixture times of Chinese Hui people. We calibrated the years using the following formula: Year = 1950-28^*^(Generation-1). To validate the concordance of our results, we also estimated the admixture times of Uyghur populations, and our observed admixture patterns were consistent with previous reports (Loh et al., 2013).

### Y-Chromosomal and mtDNA Haplogroup Assignments

We followed the recommendations of ISOGG and used our in-house script to assign the Y-chromosomal paternal lineages and used HaploGrep 2 to assign mitochondrial maternal haplogroups (Weissensteiner et al., 2016).

## Results

### General Patterns of Genetic Structure Revealed by Principal Component Analysis and ADMIXTURE Analysis

**Figure 1:**
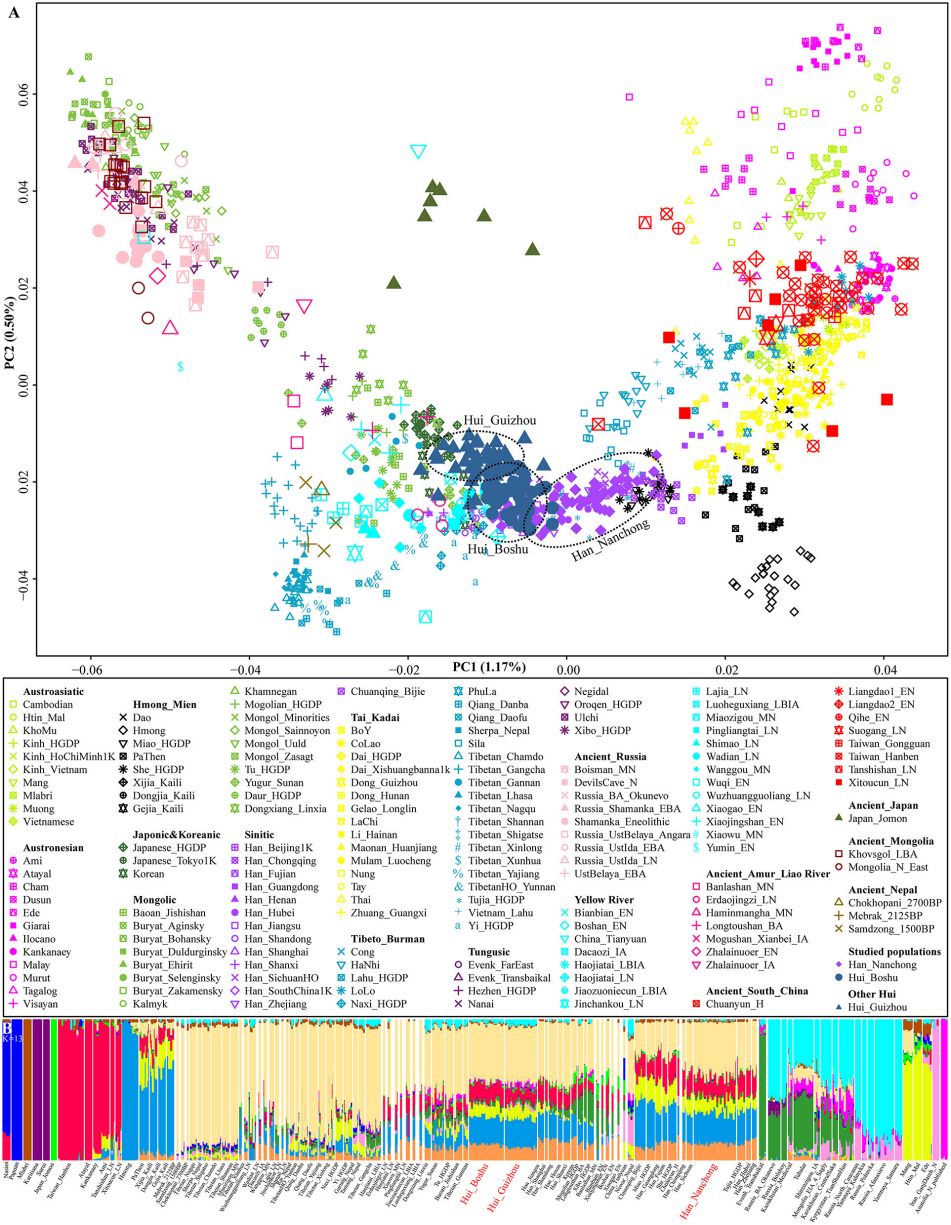
Overview of population structure. **(A)** Principal component analysis (PCA) based on the top two components of East Asian genetic variations. Included ancient individuals were projected onto the modern genetic background. **(B)** Ancestry component composition between Boshu Hui, Nanchong Han, and other Eurasian reference populations when the optimal *K* value is equal to 13.

We generated whole-genome single-nucleotide polymorphism (SNP) data from 109 Sichuan Hui and Han individuals in Sichuan Province and co-analyzed them with publicly available modern and ancient DNA data for comprehensive population genetic history reconstruction. We grouped modern reference individuals from Eurasia according to their language family categories [including Altai or Trans-Eurasian (Turkic, Mongolic, Tungusic, Japonic, and Koreanic), Hmong-Mien, Tai-Kadai, Austronesian, Austroasiatic, and Sino-Tibetan (Sinitic and Tibeto-Burman)] and classified ancient people based on their geographical affinities (Figure 1). We first carried out principal component analysis (PCA) to obtain a broad overview of the population structure of all modern eastern Eurasians, and ancient individuals were projected along the top two PC axes (Figure 1A). This inference revealed four genetic clusters (the northeastern Tungusic/Mongolic cluster, southwestern Tibeto-Burman cluster, southern inland Hmong-Mien cluster, and southern Austronesian/Austroasiatic cluster) and one Sinitic-Tai-Kadai cline. Boshu Huis were clustered with northern Han Chinese and other Northern East Asian minorities, and Nanchong Hans were loosely clustered and formed a cline that partially overlapped with previously published Han clines. Previously reported Guizhou Hui people grouped closely with the Boshu Hui people but showed a deviation toward northeastern Tungusic/Mongolic speakers. We also found a close genetic affinity between Boshu Huis and late-Bronze-Age-to-Iron-Age Northern East Asians from Henan Province (Haojiatai and Luoheguxiang). In the Southern East Asian-based PCA, we observed clear population substructures that were consistent with the language categories, such as the Hmong-Mien and Sino-Tibetan genetic lines being separated from others (Supplementary Figure 3). We expected to observe a unique genetic cluster or cline of Huis if these individuals possessed unique western Eurasian-originated ancestry. However, we found that the studied Han and Hui populations were localized in an intermediate position between highland East Asians and southern East Asians. Furthermore, we performed PCA of Eurasians, including an additional five European populations, and found that both Hui populations (Boshu and Guizhou) showed an affinity to western Eurasians relative to geographically close Hans (Supplementary Figure 4).

Model-based ADMIXTURE results with the optimal *K* value (*K* = 13) revealed multiple sources of East Asian-dominant ancestry that were enriched in both inland (Hmong) and coastal (Ami) Southern East Asians, and others were dominated by inland (Tibetan) and coastal (Japanese) Northern East Asians (Supplementary Figures 2, 5). Our studied Sichuan Huis shared the most genetic ancestry with Northern East Asians (Tokyo-Japanese-like: 0.123 and Mebrak_2025BP-like: 0.398) and Southern East Asians (Hmong-like: 0.208, Taiwan_Hanben-like: 0.116 and Htin_Mal-like: 0.082) (Figure 1B and Supplementary Table 4). We also found ancestry related to western Eurasia (Russia_Poltavka: 0.008). Adjoining Nanchong Hans possessed dominant Northern East Asian ancestry (Japanese: 0.130 and Mebrak: 0.344) and Southern East Asian ancestry (Hmong: 0.230, Htin: 0.116, and Hanben: 0.140). We also performed a Wilcoxon test to explore the differences in ancestry proportions related to western Eurasians. Compared with the Boshu Huis, the Guizhou Huis harbored more ancestry related to steppe pastoralists (Poltavka: 0.008 vs. 0.027, *Z* = −6.513 and *p* < 10^−3^), Iranian farmers (Iran_GanjDareh_N: 0.006 vs. 0.015, *Z* = −4.787 and *p* < 10^−3^), and Anatolian farmers (Anatolia_N: 0.010 vs. 0.027, *Z* = −6.803 and *p* < 0.000). The patterns of genetic relationships revealed by model-based ADMIXTURE analysis were consistent with the PCA results.

### Genomic Affinity Inferred From Fst and Shared Genetic Drift

To quantitatively evaluate the genetic differences between Hui and Han populations from Sichuan Province and 170 modern and 67 ancient Eurasian populations with a population size larger than five, we calculated the pairwise Fst genetic distances (Supplementary Table 5) according to Weir and Cockerham (Weir and Cockerham, 1984). The Fst-based comparison of genetic differences showed that Chinese Hui populations were most closely related to geographically close northern Han populations, such as Han populations from Nanchong (0.0019) and Beijing (0.0013). Among the ancient populations, the Boshu Huis had the closest genetic relationship with Mongolia's Early Iron Age SlabGrave people (Mongolia_EIA_1_SlabGrave: 0.0603), followed by Russia Shamanka Eneolithic people (0.0675) and central and western Mongolia Late Bronze Age people (Mongolia_LBA_4_CenterWest: 0.0680). We also detected close genetic relationships between northern East Asians and Guizhou Huis, as well as a relatively close relationship between Guizhou Huis and Boshu Huis (0.0095). However, a subtly different pattern of genetic affiliation of Nanchong Hans and their modern and ancient eastern Eurasian reference populations was identified in the Fst matrixes. Nanchong Hans tended to show a close relationship with central and southern Han Chinese (Zhejiang Hans: 0.0003, Hubei Hans: 0.0005 and Sichuan Hans: 0.0006).

**Figure 2:**
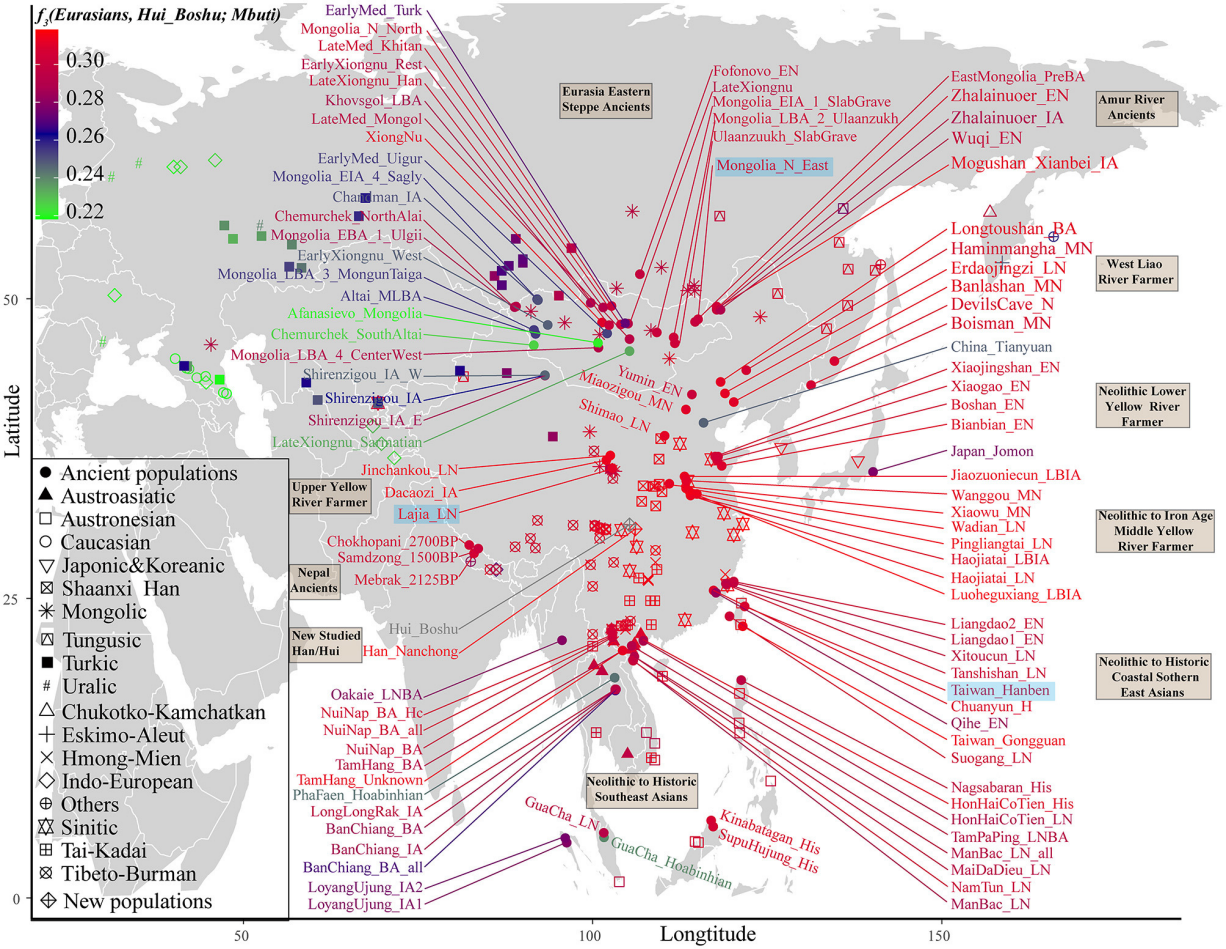
Heatmap showing the genetic affinity between Boshu Hui and modern/ancient Eurasians estimated by *f*_3_-statistic in the form *f*_3_*(Eurasian, Hui_Boshu, Mbuti)*. The red color denotes higher genetic affinity, suggesting their genetic affinity to Hui people. The green color denotes a smaller *f*_3_-value, suggesting their distant relationship with the Hui people. Here, the main ancient populations were used in the *qpGraph*-based admixture modeling marked with the light blue background. LBA, Late Bronze Age; IA, Iron Age; MLBA, Middle and Late Bronze Age; LN, Late Neolithic; EN, Early Neolithic; MN, Middle Neolithic; LBIA, Late Bronze Age and Iron Age.

**Figure 3:**
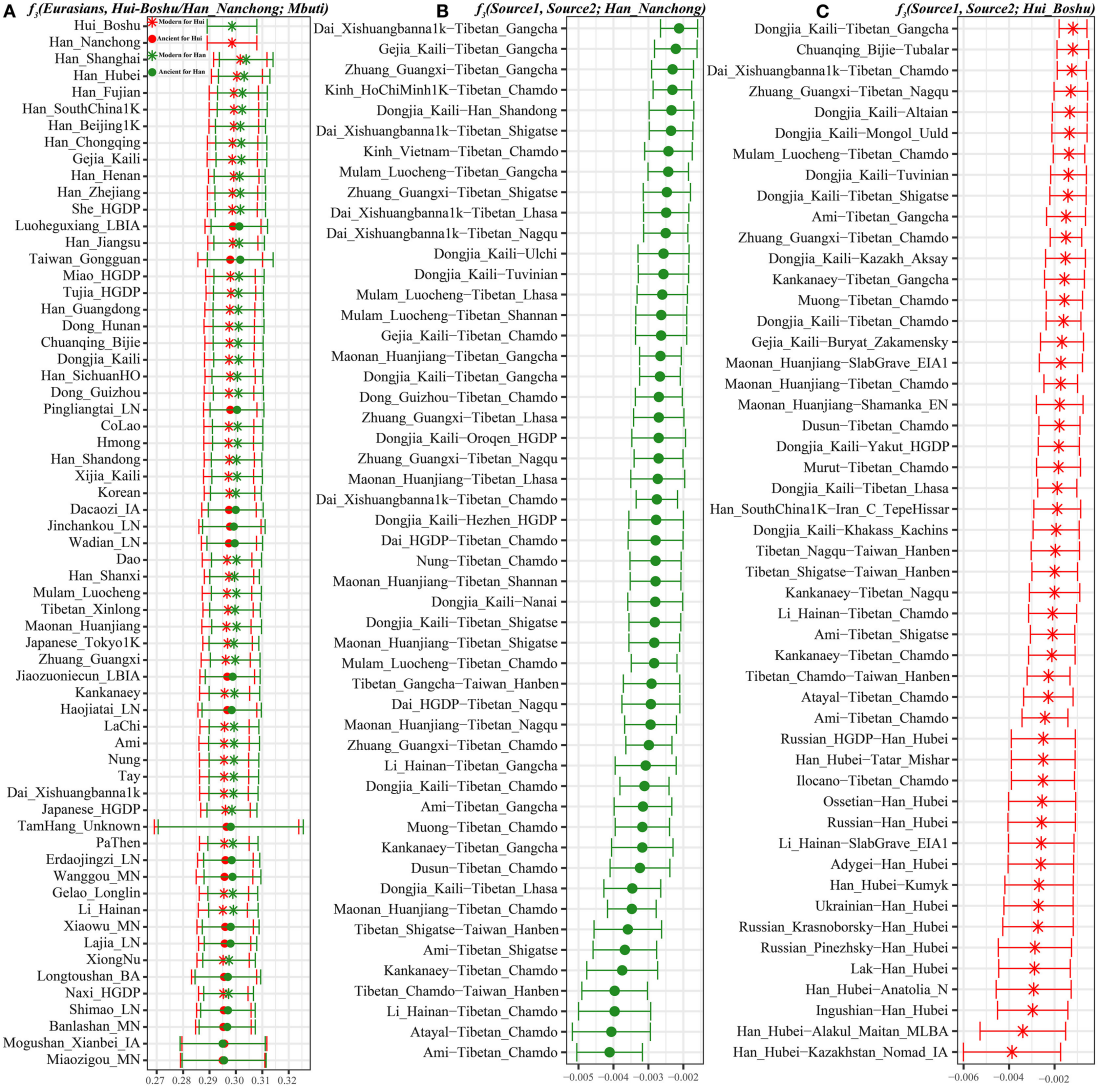
The shared genetic drift and admixture signal for the targeted Han and Hui populations. **(A)**. The top 60 populations harboring genetic affinity with Boshu Hui (red color) and Nanchong Han (green color). **(B,C)**. Top sixty pairs of source populations possessing admixture signals via three-population testing in the form of *f*_3_*(Source1, source2; Boshu Hui/Nanchong Han)*. All showed population pairs with Z-scores less than negative three.

We subsequently used a formal test of outgroup-*f*_3_ statistics in the form *f*_3_*(Test populations, Hui_Boshu/Han_Nanchong; Mbuti)* to explore the shared genetic drift between two studied populations and Eurasian reference populations, including 188 modern and 176 ancient populations, which may have contributed genetic materials to the Hui and Han gene pools in Sichuan Province (Supplementary Table 6 and Figures 2, 3A, Supplementary Figure 6). We found that Boshu Huis possessed the most shared ancestry with modern Han groups from Hubei (0.3004), Fujian (0.2992), and Henan (0.2992) provinces and ancient Iron Age Luoheguxiang (0.2989) and late Neolithic Pingliangtai (0.2978), after excluding some groups with bias introduced via sample size or batch effects. Although some differences in the patterns of genetic affinity were identified via Fst and *f*_3_ statistics, all these groups showed an affinity with modern Hans. Nanchong Hans shared the most genetic drift with modern Han groups from Hubei (0.3031), Fujian (0.3025), and Chongqing (0.3020) provinces and ancient Iron Age Luoheguxiang (0.3013) and late Neolithic Pingliangtai (0.3004). Small amounts of shared genetic drift between the studied groups and archaic peoples (Denisovan: 0.0217 in Hans and 0.0224 in Huis; Neanderthal: 0.0249 in Hans and 0.0251 in Huis) were identified. There was a strong statistically significant correlation of shared ancestry between Hui and Han individuals in the outgroup *f*_3_ values (*f*_3__Han = 0.9579^*^f_3__Hui + 0.0099; *R*^2^ = 0.9992). The correlations of shared genetic drift with latitude (0.1817 in Hans and 0.1691 in Huis) and longitude (0.6992 in Hans and 0.6777 in Huis) showed an association between shared genetic drift and longitude, which was consistent with the gradient changes in the *f*_3_-based heatmap (Supplementary Table 6). Furthermore, we also identified strong genomic affinity between Boshu Huis and Guizhou Huis based on shared genetic drift (*f*_3__Bohui_Hui = 0.941^*^f_3__Guizhou_Hui + 0.0139). However, the Guizhou Huis had a smaller amount of shared genetic drift (0.2918) with Boshu Huis than with other East Asian reference groups.

### Phylogenetic Relationships Inferred From TreeMix and Neighbor-Joining Trees

To comprehensively evaluate the overall genetic relationships and cluster patterns of Eurasians, we constructed the most representative neighbor-joining (N-J) tree to date among 190 modern and 176 ancient Eurasian populations based on inverted *f*_3_-based genetic matrixes (*1/f*_3_). We found that the Neanderthal and Denisovan populations were fitted at the root in the reconstructed phylogeny and detected genetic differentiation between modern eastern and ancient Eurasian populations (Supplementary Figure 7). Ancient populations were clustered closely with their geographically close modern populations, suggesting the long-term continuity of primary ancestry in the genetic transformation: Southeastern Neolithic-to-Historic people clustered with modern Austronesians; late-Neolithic-to-Historic Southeast Asians grouped with modern Austroasiatic and Tai-Kadai; ancient Nepal Tibetans clustered with modern highland Tibeto-Burman speakers; Northern East Asians from the Yellow River basin grouped with modern Sinitic people; Siberian ancients mainly clustered with modern Mongolic, Tungusic, and Uralic people; and ancient western Eurasians grouped with modern Indo-European or Turkic people. We found a close genetic relationship between Boshu Huis and Nanchong Hans, which in turn clustered with the geographically close Sichuan Hans. The close phylogenetic relationships between the studied populations and Eurasian modern reference populations were further confirmed via a TreeMix-based phylogeny with two population sets (Supplementary Figure 8). Among a large reference population set consisting of 47 Eurasian populations as representatives from the main language families and Mbuti as the root, we identified western gene flow events into the Kazakh population but not into Huis (Supplementary Figure 8A). However, when we included the studied populations, 16 East Asians with limited contributions from western Eurasians and five western Eurasians with limited East Asian ancestry in the new TreeMix-based model, we identified one gene flow event into Boshu Huis from the deep East Asian lineage (Supplementary Figure 8B). These observed patterns of population splits and admixture supported the strong genetic affinity between the Boshu Huis and modern East Asians and a small amount of western Eurasian-like gene influx.

### Mixture Signatures Revealed From Admixture-f_3_ Statistics

To explore the potential ancestral sources of Huis and Hans among these reference datasets, we calculated admixture-*f*_3_ statistics in the form *f*_3_*(Source1, source2; Hui_Boshu/Han_Nanchong)*, which would be expected to yield statistically negative values (Z-scores < −3) if the allele frequencies of Huis/Hans were intermediate between the frequencies of source1 and source2. We found signals supporting two-way or three-way admixture models for the genetic formation of Sichuan Huis and Hans (Supplementary Table 7). North–south admixture signatures were identified when we treated Nanchong Hans as the target population (Supplementary Table 7 and Figure 3B). Here, we could also identify genetic contributions from central and southeastern Han Chinese to Sichuan Hans via the negative values from *f*_3_*(Han_Fujian/Guangdong/Hunan, other sources; Han_Nanchong)*, suggesting the possible westward movement of Han Chinese over historical time. Focusing on the Chinese Huis, we obtained most admixture signals among the source pairs of Northern East Asians (Tibetans and southern Siberians) and Southern East Asians (Tai-Kadai and Austronesian speakers), such as *f*_3_*(Ami, Tibetan_Chamdo; Hui_Boshu)* = −7.216^*^SE (standard error). Western Eurasian admixture signals could also be obtained when we used Han Chinese as one source and Ingushian (−5.748), Lak (−5.508), or Adygei (−5.429) as the other source (Supplementary Table 7 and Figure 3C).

### Differentiated Demographic Histories Estimated From *f*_4_ Statistics

To explore the genetic heterogeneity between Hui people and their neighbors (Hans and geographically different Huis), we first calculated *f*_4_ statistics in the form *f*_4_*(Han_Nanchong, Hui_Boshu; Eurasians, Mbuti)*. Excess shared alleles between Nanchong Hans and modern and ancient Southern East Asians were identified through significant positive values, suggesting that neighboring Han individuals had more East Asian ancestry than Hui people (Table 1 and Supplementary Tables 8, 9), especially for the Southern East Asian affinity in *f*_4_*(Han_Nanchong, Hui_Boshu; Maonan_Huanjiang, Mbuti)* = 12.351^*^SE in the Human Origin dataset and *f*_4_*(Han_Nanchong, Hui_Boshu; Taiwan_Hanben, Mbuti)* = 13.599^*^SE in the 1240K dataset. However, no statistically significant negative *f*_4_ values for *f*_4_*(Han_Nanchong, Hui_Boshu; western Eurasians, Mbuti)* were identified in the Human Origin dataset, suggesting that the studied populations formed a clade compared with western Eurasians based on the lower-density dataset. Although weak statistical significance for negative *f*_4_ values is shown in Table 1, we still observed slight gene flow from modern/ancient western Eurasians to Chinese Huis though more allele sharing of western pastoralists with Huis than with Nanchong Hans, as several reference sources provided negative *f*_4_ values with absolute Z-scores larger than 2. This pattern could be validated via further whole-genome sequencing data with more SNPs and more rare variants. Indeed, we observed statistically significant *f*_4_ values when we used Iran_C_TepeHissar (−3.924^*^SE), Russia_Srubnaya_Alakul (−3.88^*^SE), Iran_IA_Hasanlu (−3.449^*^SE), Russia_Srubnaya (−3.129^*^SE), and Russia_MLBA_Sintashta (−3.019^*^SE) as the western Eurasian sources. Furthermore, we also detected relatively close genomic affinity between Huis and geographically close Hans relative to western Eurasians via significant negative *f*_4_ values for *f*_4_*(Western Eurasian, Han_Nanchong; Hui_Boshu, Mbuti)* based on two merged datasets (Supplementary Tables 10, 11).

**Table 1:** Differentiated shared alleles between Boshu Hui and Nanchong Han compared to modern and ancient Eurasians estimated via symmetrical *f*_4_-statistics in the form *f*_4_*(Han_Nanchong, Hui_Boshu; Eurasian, Mbuti)* based on the merged 1,240 K and Human Origin datasets.

**W**	**X**	**Y**	**Z**	***f*4**	**Std err**	**Z score**	**BABA**	**ABBA**	**nsnps**	**Group**		
Han_Nanchong	Hui_Boshu	Mordovian	Mbuti	−0.0002	8.00E-05	−2.833	4,318	4,335	70,948	Uralic	Hui harbored	Merged
Han_Nanchong	Hui_Boshu	Russia_Andronovo	Mbuti	−0.0003	0.000102	−2.677	4,292	4,311	70,707	Ancient_Russia	more allele	Human Origin
Han_Nanchong	Hui_Boshu	Russia_Srubnaya	Mbuti	−0.0002	9.10E-05	−2.55	4,294	4,311	70,660	Ancient_Russia	sharing of	dataset
Han_Nanchong	Hui_Boshu	Karelian	Mbuti	−0.0002	8.10E-05	−2.505	4,312	4,327	70,709	Uralic	Western	
Han_Nanchong	Hui_Boshu	Russia_MLBA_Sintashta	Mbuti	−0.0002	8.70E-05	−2.385	4,281	4,296	70,591	Ancient_Russia	Eurasian	
Han_Nanchong	Hui_Boshu	Kumyk	Mbuti	−0.0002	8.10E-05	−2.285	4,295	4,308	70,948	Turkic	ancestry related	
Han_Nanchong	Hui_Boshu	Russia_Sintashta_MLBA	Mbuti	−0.0003	0.000116	−2.174	3,838	3,854	64,020	Ancient_Russia	to Han	
Han_Nanchong	Hui_Boshu	Russia_Afanasievo	Mbuti	−0.0002	8.70E-05	−2.105	4,295	4,308	70,650	Ancient_Russia		
Han_Nanchong	Hui_Boshu	Veps	Mbuti	−0.0002	8.40E-05	−2.065	4,314	4,327	70,709	Uralic		
Han_Nanchong	Hui_Boshu	Taiwan_Hanben	Mbuti	0.0011	9.20E-05	11.451	4,583	4,508	70,752	Ancient_China	Han possessed	
Han_Nanchong	Hui_Boshu	Atayal	Mbuti	0.0011	9.40E-05	11.5	4,583	4,506	70,948	Austronesian	more southern	
Han_Nanchong	Hui_Boshu	Kinh_Vietnam	Mbuti	0.0010	8.60E-05	11.526	4,557	4,487	70,586	Austroasiatic	East Asian	
Han_Nanchong	Hui_Boshu	Zhuang_Guangxi	Mbuti	0.0010	8.50E-05	11.622	4,580	4,510	70,836	Tai-Kadai	ancestry related	
Han_Nanchong	Hui_Boshu	Li_Hainan	Mbuti	0.0011	9.50E-05	11.639	4,590	4,512	70,836	Tai-Kadai	to Hui	
Han_Nanchong	Hui_Boshu	Vietnamese	Mbuti	0.0010	8.70E-05	11.655	4,563	4,492	70,586	Austroasiatic		
Han_Nanchong	Hui_Boshu	Muong	Mbuti	0.0010	8.80E-05	11.694	4,558	4,485	70,586	Austroasiatic		
Han_Nanchong	Hui_Boshu	Kinh_HoChiMinh1K	Mbuti	0.0010	8.30E-05	11.79	4,583	4,514	70,948	Austroasiatic		
Han_Nanchong	Hui_Boshu	Dai_Xishuangbanna1k	Mbuti	0.0010	8.40E-05	11.92	4,589	4,519	70,948	Tai-Kadai		
Han_Nanchong	Hui_Boshu	Nung	Mbuti	0.0010	8.70E-05	11.981	4,564	4,490	70,586	Tai-Kadai		
Han_Nanchong	Hui_Boshu	Maonan_Huanjiang	Mbuti	0.0011	8.60E-05	12.351	4,586	4,511	70,836	Tai-Kadai		
Han_Nanchong	Hui_Boshu	Iran_C_TepeHissar	Mbuti.DG	−0.0003	0.000078	−3.924	9,193	9,238	148,815	Ancient_Iran	More allele	Merged
Han_Nanchong	Hui_Boshu	Russia_Srubnaya_Alakul	Mbuti.DG	−0.0003	0.000073	−3.88	10,748	10,797	172,022	Ancient_Russia	sharing of	1,240 k
Han_Nanchong	Hui_Boshu	Iran_IA_Hasanlu	Mbuti.DG	−0.0004	0.000105	−3.449	9,142	9,196	147,968	Ancient_Iran	ancient western	dataset
Han_Nanchong	Hui_Boshu	Russia_Srubnaya	Mbuti.DG	−0.0002	0.000074	−3.129	10,521	10,560	168,316	Ancient_Russia	Eurasian	
Han_Nanchong	Hui_Boshu	Russia_MLBA_Sintashta	Mbuti.DG	−0.0002	0.000068	−3.019	10,575	10,610	169,259	Ancient_Russia	ancestry of Hui	
Han_Nanchong	Hui_Boshu	Russia_Afanasievo	Mbuti.DG	−0.0002	0.000071	−2.93	10,619	10,655	169,744	Ancient_Russia	related to Han	
Han_Nanchong	Hui_Boshu	Iran_C_HajjiFiruz	Mbuti.DG	−0.0002	0.000082	−2.738	9,570	9,605	154,368	Ancient_Iran		
Han_Nanchong	Hui_Boshu	Mongolia_EBA_2_Chemurchek	Mbuti.DG	−0.0003	0.000101	−2.689	8,785	8,823	139,943	Ancient_Mongolia		
Han_Nanchong	Hui_Boshu	Iran_C_SehGabi	Mbuti.DG	−0.0002	0.00008	−2.671	9,563	9,596	154,829	Ancient_Iran		
Han_Nanchong	Hui_Boshu	Russia_Alan	Mbuti.DG	−0.0002	0.000079	−2.643	10,605	10,641	170,839	Ancient_Russia		
Han_Nanchong	Hui_Boshu	Kazakhstan_Botai	Mbuti.DG	−0.0003	0.000108	−2.616	9,844	9,889	156,181	Ancient_Kazakhstan		
Han_Nanchong	Hui_Boshu	Russia_Andronovo	Mbuti.DG	−0.0002	0.000082	−2.559	10,643	10,678	170,623	Ancient_Russia		
Han_Nanchong	Hui_Boshu	Yamnaya_Kalmykia_EBA	Mbuti.DG	−0.0002	0.000084	−2.536	10,089	10,123	162,363	Ancient_Russia		
Han_Nanchong	Hui_Boshu	Russia_Sintashta_MLBA	Mbuti.DG	−0.0002	0.000089	−2.334	9,161	9,192	148,661	Ancient_Russia		
Han_Nanchong	Hui_Boshu	Chemurchek_southAltai	Mbuti.DG	−0.0002	0.000107	−2.265	9,328	9,364	148,332	Ancient_Mongolia		
Han_Nanchong	Hui_Boshu	Yamnaya_Samara_EBA	Mbuti.DG	−0.0002	0.000079	−2.124	10,338	10,365	165,089	Ancient_Russia		
Han_Nanchong	Hui_Boshu	LateMed_Khitan	Mbuti.DG	0.0007	0.000099	6.91	9,560	9,461	145,659	Ancient_Mongolia	Han possessed	
Han_Nanchong	Hui_Boshu	Wadian_LN	Mbuti.DG	0.0007	0.000098	6.929	11,203	11,088	169,529	Ancient_China	more ancient	
Han_Nanchong	Hui_Boshu	Taiwan_Gongguan	Mbuti.DG	0.0009	0.000131	7.028	7,024	6,927	106,500	Ancient_China	East Asian	
Han_Nanchong	Hui_Boshu	Liangdao1_EN	Mbuti.DG	0.0011	0.000159	7.17	3,734	3,669	57,273	Ancient_China	ancestry	
Han_Nanchong	Hui_Boshu	Dacaozi_IA	Mbuti.DG	0.0006	0.00009	7.22	10,876	10,770	164,849	Ancient_China	compared with	
Han_Nanchong	Hui_Boshu	Erdaojingzi_LN	Mbuti.DG	0.0008	0.000103	7.305	9,316	9,209	141,199	Ancient_China	Hui	
Han_Nanchong	Hui_Boshu	Liangdao2_EN	Mbuti.DG	0.0009	0.000121	7.464	8,727	8,607	132,852	Ancient_China		
Han_Nanchong	Hui_Boshu	Pingliangtai_LN	Mbuti.DG	0.0008	0.000091	8.303	11,292	11,164	170,579	Ancient_China		
Han_Nanchong	Hui_Boshu	Xitoucun_LN	Mbuti.DG	0.0011	0.000125	8.638	5,660	5,567	85,992	Ancient_China		
Han_Nanchong	Hui_Boshu	Taiwan_Hanben	Mbuti.DG	0.0011	0.000079	13.599	11,325	11,142	170,610	Ancient_China		

Additionally, we calculated these types of *f*_4_ statistics (Supplementary Table 12) to explore the genetic heterogeneity between Guizhou Huis and Boshu Huis, and we found that Guizhou Huis harbored excess shared alleles related to western steppe pastoralists compared to Boshu Huis. Focusing on the differentiated genetic contributions to Huis of eastern Eurasians and western Eurasians, we calculated *f*_4_*(Eastern Eurasian, Western Eurasian; Hui_Boshu/Hui_Guizhou, Mbuti)* and *f*_4_*(Eastern Eurasian, Hui_Boshu/Hui_Guizhou; Western Eurasian, Mbuti)*. As shown by the significant positive values in Supplementary Table 13, compared with populations harboring higher western Eurasian ancestry, two Hui populations shared more eastern Eurasian-related derived alleles. However, compared with the representative populations from East Asia, modern western populations and ancient steppe pastoralist populations shared more ancestry with two Hui populations, as more statistically negative *f*_4_ values are shown in Supplementary Table 14. The identified differentiated population structure revealed the different demographic histories of these groups.

Previous genetic studies have demonstrated that the historically/archeologically supported spread of millet and rice agriculture in East Asia was accompanied by the bidirectional spread of farmers from the northern Yellow River basin and southern Yangtze River basin (Yang et al., 2020). They also revealed significant population stratification among East Asians according to language categories. Next, we focused on the population differentiation between Huis and East Asians (Wang et al., 2021). As shown in Supplementary Table 15, most statistically positive *f*_4_ values observed among modern Eastern panels based on the merged 1,240 K dataset demonstrated that the Boshu Huis harbored more East Asian-derived ancestry than their respective predefined comparative populations, which is consistent with the genetic affinity identified via the outgroup *f*_3_ statistics and pairwise *f*_4_ analysis based on the Human Origin dataset (Supplementary Table 16). To further explore the demographic process with the plausible differentiated genetic contributions to our studied populations, we calculated *f*_4_*(East Asian reference1, Hui_Boshu; East Asian reference2, Mbuti)* using all possible East Asian pairs (Supplementary Figures 9–14 and Supplementary Tables 17, 18). Population clustering based on the Z-scores of *f*_4_ statistics showed that modern Austronesian, Tai-Kadai, and Sino-Tibetan-speaking populations (Ami, Atayal, Dai, Han, Thai, Kinh, Lahu, She, and Miao fixed as reference 2) and ancient Hanben peoples shared more alleles with Huis than other East Asians (Supplementary Figure 9 and Supplementary Table 17) based on the 1,240 K dataset. The Human Origins dataset had more representative populations with a high coverage of geographic and linguistic features. Thus, when fixed Sino-Tibetan speakers and northern East Asians were used as reference 2 in the Human Origins dataset, we found that Sino-Tibetan and Northern East Asians shared more alleles with Huis than with other East Asians, in contrast to non-Sino-Tibetan and Southern East Asians (Supplementary Figures 10, 11 and Supplementary Table 18).

**Figure 4:**
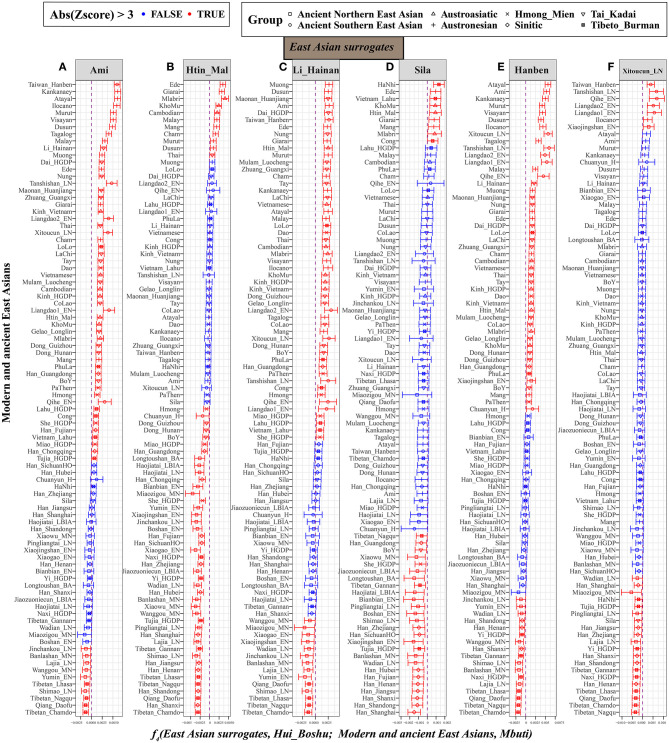
Higher gene flow in Hui from Sino-Tibetan speakers and ancient Northern East Asian in Hui than other East Asian populations inferred from *f*_4_*(East Asian surrogates, Hui_Boshu; East Asians, Mbuti)*. This type of affinity-*f*_4_-statistics was used to explore the genetic continuity and admixture between potential ancestral sources and Boshu Hui based on the merged Human Origin dataset. East Asians were listed in the left parts of the bar plots, and we used Ami **(A)**, Htin_Mal **(B)**, Li_Hainan **(C)**, Sila **(D)**, Hanben_Taiwan **(E)**, and Xitoucun_LN **(F)** as the surrogates of our focused East Asians. All Y-axis-related populations were sorted via the Z-scores. The bar denotes the three times of the standard errors. The red color denotes East Asians (left population lists) sharing more derived alleles with Boshu Hui compared with our used East Asian surrogates. The green color denotes the surrogate population formed with one clade with Hui people. Statistically significant *f*-statistics were marked as red colors.

Furthermore, we fixed reference 1 as the East Asian representative populations (left-column populations in Supplementary Figure 10). When focusing on geographically different Han Chinese, positive *f*_4_ values from *f*_4_*(Sinitic, Hui_Boshu; East Asian, Mbuti)* suggested that other East Asians shared more alleles with Han Chinese than with Hui people. Significant positive values for *f*_4_(Southern East Asians. Hui_Boshu; East Asians, Mbuti) showed a similar affinity pattern, but Sino-Tibetan and ancient northern East Asians shared more alleles with Hui people than with others. Thus, we further chose the Austronesian-speaking Ami, Austroasiatic-speaking Htin, Tai-Kadai-speaking Li, Tibeto-Burman-speaking Sila, and ancient southern East Asians of Hanben as the surrogates in the *f*_4_ statistic calculations. As detailed in Figure 4, compared with Ami, Tibeto-Burman speakers (Z-scores of Tibetan_Chamdo: −6.438; Qiang_Daofu: −6.379; Tibetan_Nagqu: −5.235 and Tibetan_Lhasa: −4.7) and ancient Northern East Asians (Shimao_LN: −4.889; Yumin_EN: −3.816; Wanggou_MN: −3.806; Lajia_LN: −3.798; Banlashan_MN: −3.615 and Jinchankou_LN: −3.469) shared more alleles with Hui people (Figure 4A). Similar genetic patterns were further confirmed via negative *f*_4_ values for *f*_4_*(Li/Htin, Hui_Boshu; northern East Asians, Mbuti)* (Figures 4B,C). Furthermore, compared with the pattern observed for the lowland Tibeto-Burman Sila people (Figure 4D), higher gene flow to Huis from the Sinitic speakers than from other East Asian populations was evidenced by the negative values for *f*_4_*(Sila, Hui_Boshu; Han, Mbuti)*. We also found that Neolithic-to-Iron-Age populations from the Yellow River basin shared more derived alleles with Boshu Huis than with ancient Southern East Asians, which also pointed to predominant East Asian assimilation and the stronger Neolithic-to-modern Northern East Asian affinity of the modern Huis as supporting evidence from *f*_4_*(Hanben_Taiwan/Xitoucun_LN, Hui_Boshu; Northern East Asians, Mbuti)* (Figures 4E,F). These genetically supported patterns of genetic admixture signatures were also confirmed and supported by evidence from the 1,240 K dataset (Supplementary Table 17). Interestingly, we found that the middle Yellow River farmers formed one clade with the Boshu Huis when compared with modern East Asians when reference 1 was fixed as the ancient Northern East Asians from the Yellow River basin (Supplementary Figure 10). Overall, the shared genetic profiles between Huis and East Asians not only illuminated higher gene flow in Huis from Sinitic speakers than from other East Asian populations but also suggested higher gene flow in Huis from Yellow River farmers than from other East Asian populations.

Finally, our data can also provide some new insights into the genomic history of geographically adjacent Han Chinese populations. As shown in Supplementary Figures 12–14 and Supplementary Tables 15–18, modern and ancient East Asian populations shared more alleles with Nanchong Hans than with other reference groups, as shown by the positive *f*_4_ values in Supplementary Figures 12A, 13. Symmetric *f*_4_ statistics for the Nanchong Han population in Supplementary Figures 12B, 14 further demonstrated that Nanchong Han individuals shared more Northern East Asian-like derived alleles as well as additional gene flow from Southern East Asians. Positive values from *f*_4_*(Han_Nanchong, Hui_Boshu; modern/ancient Southern East Asians, Mbuti)* also documented more Southern East Asian-like ancestry in Nanchong Hans than in Boshu Huis (Table 1).

### Estimates of Admixture Proportion via *qpWave/qpAdm*

Considering the admixture events and sources that we observed in our studied Han and Hui populations, we applied *qpWave/qpAdm* to validate different proposed admixture scenarios. *QpWave/qpAdm* was the formal test that could evaluate whether the pattern for Hans/Huis was consistent with descent from one or more ancestral source populations relative to some chosen correct or reference outgroup populations (having a differentiated genetic relationship with the included source populations) and then evaluate their corresponding ancestral proportions. The best-fitting *qpAdm* model was dependent on the following criteria: (1) the *p*-value of the rank test was larger than 0.05, (2) there was no negative ancestry proportion, and (3) the minimum mixture proportion was larger than the corresponding standard error. To better visualize the differentiated population structure of Hui and Han populations, we applied the same analysis strategies to estimate their admixture proportion. We first simulated three different two-way admixture scenarios and then analyzed the three-way admixture models with 44 western Eurasians, 17 Northern East Asians, and three Southern East Asians as the potential ancestral sources.

First, we fit 880 pairs of two-way admixture *qpAdm* models focused on the western and eastern sources. We found that all pairs focused on Nanchong Hans failed, and 95 out of 880 pairs focused on Boshu Huis yielded fitted models (Supplementary Table 19). Here, Boshu Huis can be modeled as a mixture of major eastern Eurasian ancestry ranging from 94.6 to 98.4% in different fitted models and minor western Eurasian ancestry.

Second, we fit two-way admixture models focused on northern and southern East Asians as ancestral sources. Modeling Nanchong Hans as a mixture of ancestral Northern East Asians (Yellow River farmers) and Southern East Asians (Yangtze River farmer-related people), we found that 0.314 (Mongolia_N_North-like) ~0.891 (Haojiatai_LBIA-like) of the ancestry of Nanchong Hans related to Northern East Asians mostly associated with Yangshao and Longshan people from Henan Province (Supplementary Table 19). A higher proportion of Northern East Asian ancestry could be modeled when we used populations from later periods (Late Neolithic to historic sources) and geographically close populations from the Central Plain in North China. The Southern East Asian-related ancestry proportion was 0.109–0.686 in different two-way admixture models. These patterns of admixture were consistent with the North China Origin hypothesis of modern Sinitic speakers and continuously received gene flow from Southern East Asians. Interestingly, we also obtained 16 fitted models for Boshu Huis with peripheral ancient Northern East Asians as the northern sources, with ancestry proportions ranging from 0.386 to 0.944 among the models (Supplementary Table 19).

Third, to directly assess the genetic relationship between Boshu Huis and Nanchong Hans, we used Nanchong Hans as the East Asian source to fit two-way admixture models. We obtained 17 fitted models with five modern European and 12 western Eurasian steppe pastoralist populations as the western Eurasian source (Supplementary Table 20). The Huis can be modeled as an admixed group with 0.023–0.033 ancestry related to Western Eurasians and 0.967–0.977 ancestry related to Nanchong Hans with different source pairs.

Finally, considering the different proportions of western Eurasian ancestry in different models with different predefined ancestry sources, we further fit three-way *qpWave*-based admixture models with diverse modern or spatiotemporally different ancient western sources to explore the full landscape of the admixture process (Supplementary Table 21). Here, we found western Eurasian admixture proportions from 0.036 in the Jiaozuoniecun_LBIA-Ami-Sardinian model to 0.223 in the Jinchankou_LN-Taiwan_Hanben-Kyrgyzstan_Medieval_Nomad model for Boshu Huis and ranging from 0.017 in the DevilsCave_N-Tanshishan_LN-European model to 0.128 in the Jinchankou_LN-Taiwan_Hanben-Kyrgyzstan_Medieval_Nomad model for Nanchong Hans. The Northern East Asian ancestry in Boshu Huis ranged from 0.269 in the DevilsCave_N-Tanshishan_LN-Kyrgyzstan_Medieval_Nomad model to 0.87 in the Jiaozuoniecun_LBIA-Ami-Sardinian model, with 404 out of 477 models yielding a Northern East Asian ancestry proportion larger than 0.5. The Southern East Asian ancestry in Boshu Huis ranged from 0.094 in the Jiaozuoniecun_LBIA-Ami-Sardinian model to 0.619 in the DevilsCave_N-Taiwan_Hanben-Russia_Karasuk model, with 411 out of 477 models yielding Southern East Asian ancestry <0.5. To further compare the different genetic contributions to geographically distant Guizhou Huis, we also fit three-way admixture models with the same batch of predefined sources and outgroups, and we found the largest western Eurasian contribution in Guizhou Huis, followed by Boshu Huis. Minimal western gene flow in Nanchong Hans was also identified in the models with the same predefined sources. Overall, western Eurasian admixture increased when we used geographically close central Asians as potential source candidates.

### *qpGraph*-Based Phylogenetic Framework

**Figure 5:**
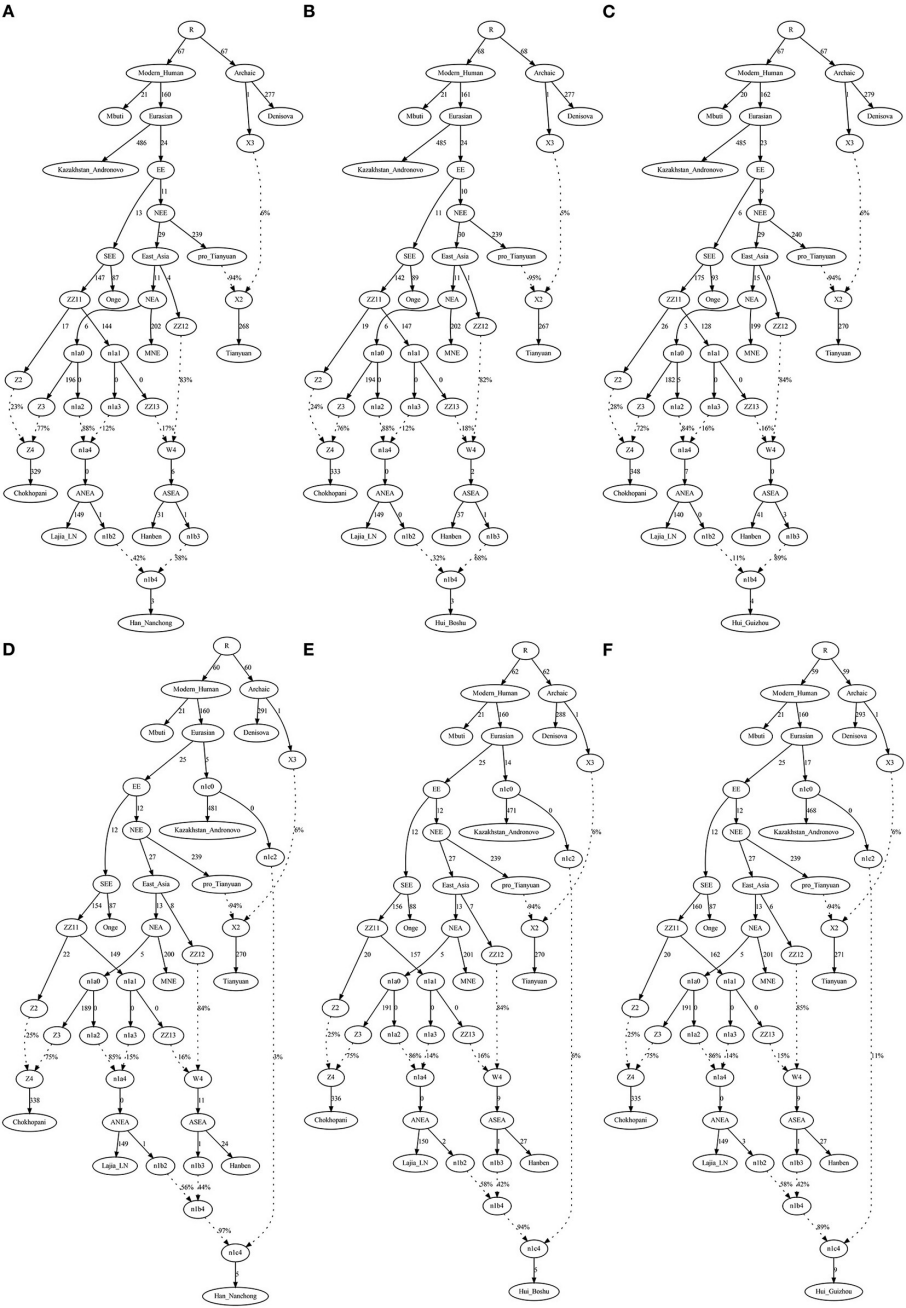
*QpGraph*-based admixture graphs illustrate that a western gene flow can improve the fitness of the deep genomic model of Chinese Hui. Branch length was marked with the *f*_2_ shared drift distance (1,000 times). Admixture events were denoted as dotted line. The admixture proportion was marked along the dotted line. **(A)** *f4(Kaz, Tia; MNE, Laj)* = 2.417*SE, Final score: 19.834. **(B)** *f4(Kaz, Tia; Han, Hiu)* = −2.897*SE, Final score: 26.782. **(C)** *f4(Kaz, Tia; Han, Hiu)* = −4.683*SE, Final score: 53.444. **(D)** *f4(Kaz, Tia; MNE, Laj)* = 2.516*SE, Final score: 18.555. **(E)** *f4(Kaz, Tia; MNE, Laj)* = 2.487*SE, Final score: 17.383. **(F)** *f4(Kaz, Tia; MNE, Laj)* = 2.488*SE, Final score: 17.994. EBA, Early Bronze Age; LN, Late Neolithic; N, Neolithic.

**Figure 6:**
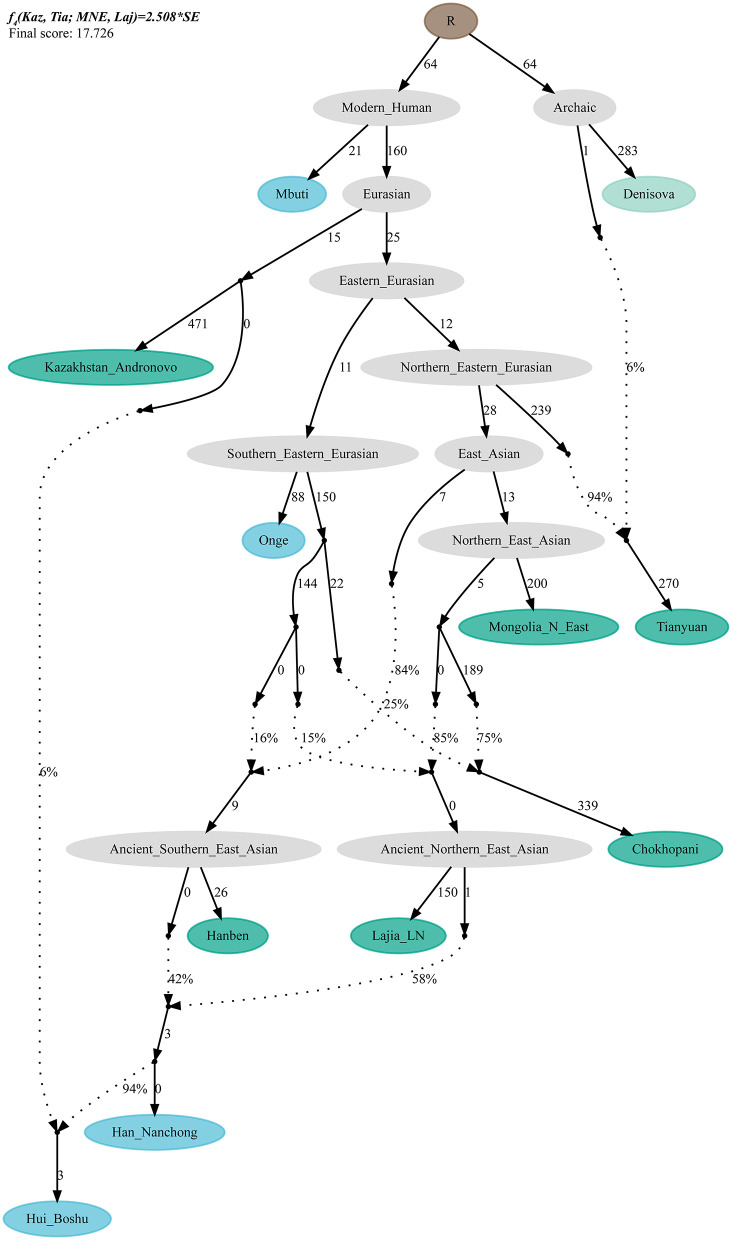
*QpGraph-based* admixture graph. *QpGraph*-based phylogeny showed the western Eurasian gene flow event in Boshu Hui. Branch length was marked with the *f*_2_ shared drift distance (1,000 times). Admixture events were denoted as dotted line. Admixture proportion was marked along the dotted line. Here, we used Hungary Scythian as the western source.

To validate the hypothesis of additional western Eurasian gene flow during Chinese Huis' genomic formation history, we used *qpGraph* to fit a phylogeny-based model with orders of population splits, branch length measured by *f*_2_ values, admixture events, and corresponding ancestral proportions. We used modern South Asian indigenous hunter–gatherer (Onge) and 40,000-year-old Tianyuan people as the early eastern Asian lineage; Mongolian Neolithic people (Mongolia_N_East), late Neolithic Qijia people (Qijia_LN), and ancient Tibetan Plateau people (Chokhopani) as proxies for the Northern East Asian lineages; and Iron Age Hanben as a Southern East Asian proxy. We obtained the best-fitting model (Model A) for the Nanchong Hans with an absolute Z-score of 2.417 and a likelihood of 19.834 (Figure 5A). Then, we replaced Nanchong Hans by Boshu Huis (Model B) and Guizhou Huis (Model C) and added additional western gene flow to the Nanchong Hans (Model D), Boshu Huis (Model E), and Guizhou Huis (Model F). We expected Model A to have a better fit than Model D and Model E/F to have a better fit than Model B/C if the hypotheses were true. Indeed, we observed the expected patterns in Figure 5. Interestingly, we found that Nanchong Hans possessed decreased likelihood scores when western Eurasian gene flow was added, although the reduction was small and Kazakhstan Andronovo contributed only 3% to the gene pool of Nanchong Hans. However, the score reduction in two Hui groups was large, which probably also suggested very low gene flow from western Eurasian sources to Nanchong Hans. Additionally, we tested several models where Huis were treated as a descendant population from Nanchong Hans with gene flow from west Eurasians from different spatiotemporally different western sources (Figure 6 and Supplementary Figures 15–31). Generally, Nanchong Han Chinese were modeled as resulting from the admixture of mostly ancient Northern East Asian ancestors related to Lajia_LN (58–82%) and some ancient Southern East Asians related to Hanben (18–42%). We successfully fitted a model with minor gene influx from western Eurasians to East Asian Hui people (~6% related to Kazakhstan Andronovo in Figure 6). We further validated this model and confirmed the minor genetic contribution from western Eurasians with temporally different central Asian or European sources (7% in Hungary Scythian people (Supplementary Figure 15), 7% in Early Neolithic Bronze Age Kalmykia Yamnaya (Supplementary Figure 16), 7% in Middle and Late Bronze Age Sintashta (Supplementary Figure 17), and 6–8% in other Iron Age to modern western Eurasians (Supplementary Figures 18–22). We used the same models to explore the evolutionary history of Guizhou Huis and found that the group harbored a relatively large amount of western gene influx, ranging from 11 to 13% in the best-fitting models (Supplementary Figures 23–31).

### Proxy of Admixture Dates Based on Weighted Linkage Disequilibrium Statistics

**Figure 7:**
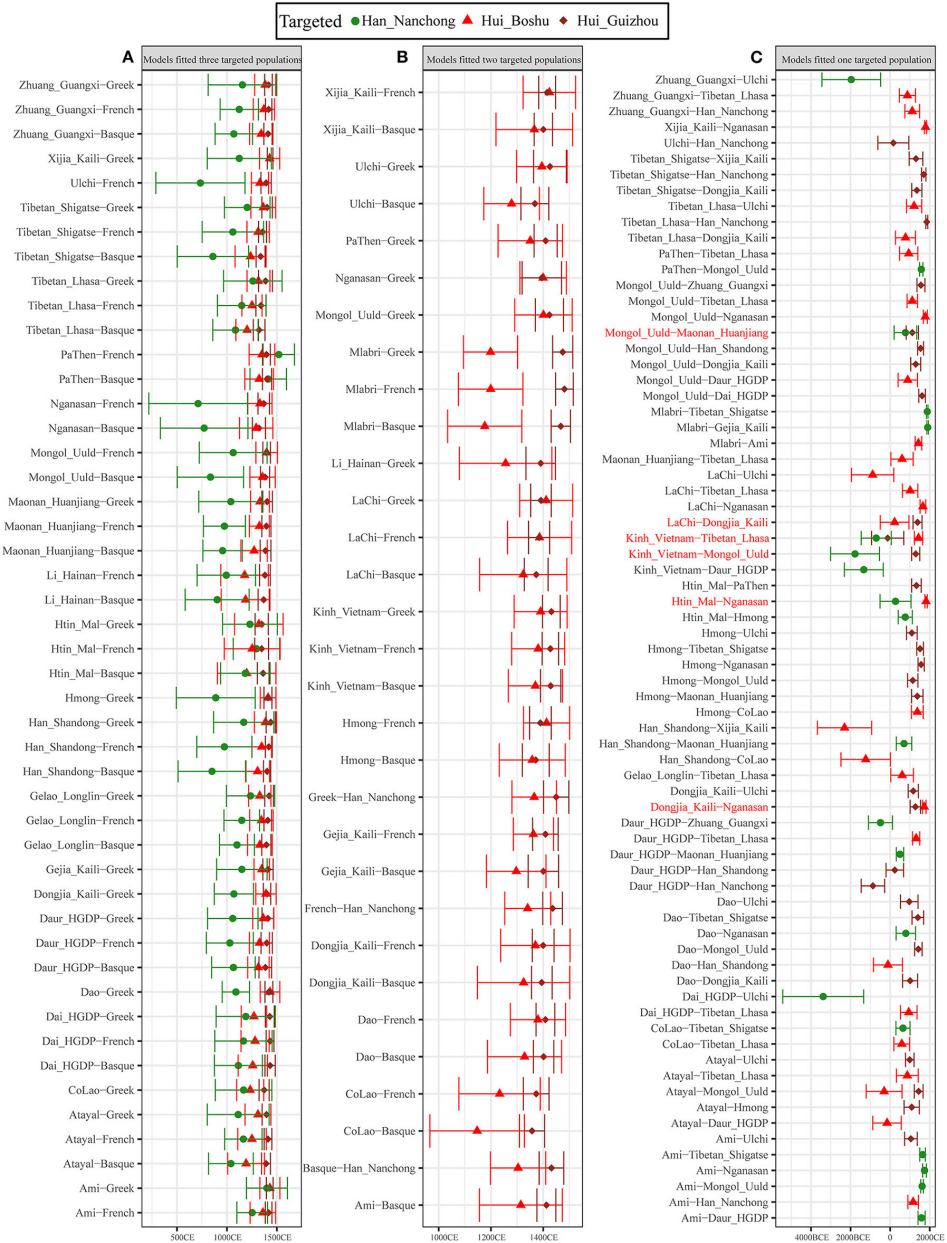
Admixture-introduced linkage disequilibrium (ALDER)-based admixture time between different northern and southern or eastern and western ancestral sources based on the 1,240 K dataset. **(A)** East–west ancestral source pairs fitted all three studied populations. **(B)** East–west ancestral source pairs fitted all two studied Hui populations but not fitted Nanchong Han. **(C)** East–west ancestral source pairs only fitted one studied population. One north–south ancestry source pair fitted three studied populations, and five north–south ancestry source pair fitted two studied populations also included in here; pairs were marked with the red color. We used 28 years as the one-generation length. All marked years in the bottom was calculated using the formula as Year = 1950-28*(Generation-1). All comprehensive raw data were presented in Supplementary Table 23.

We further used the ALDER-based method to estimate the date when western Eurasian ancestry was introduced into the gene pool of East Asian populations and reconstructed the detailed process of genetic contact between western and eastern Eurasians. We first validated whether our merged datasets were suitable for dating admixture events by examining the admixture history of the Uyghur population, which was genetically characterized as admixture between French and Hans 20 generations ago (Loh et al., 2013). Our results also showed evidence of admixture for Uyghurs (Supplementary Table 22) ~17.9 ± 1.65 generations (501.2 years) ago in the French–Nanchong Han model and 18.86 ± 1.79 generations (528.08 years) ago in the French–Shandong Han model, which suggested that our merged datasets were suitable for inferring the Sichuan Hui admixture process. Thus, we modeled the admixture process of Hui people using geographically close Han individuals as the East Asian source and found that the number of generations of admixture ranged from 18.08 ± 2.62 in the French–Han model to 25.35 ± 7.27 in the Tajik–Han model. For Guizhou Huis, we observed more recent admixture times, ranging from 17.29 ± 3.18 (Basque–Han_Nanchong) to 18.81 ± 1.9 (French–Han_Nanchong) generations based on the merged 1,240 K dataset and from 18.87 ± 1.73 (Han_Nanchong–Greek) to 19.51 ± 1.68 (Han_Nanchong–Basque) generations based on the merged Human Origin dataset. Then, we estimated the admixture times using other potential East Asian sources based on two datasets (Figure 7 and Supplementary Figure 31) and found that admixture events mainly occurred during historic times ranging from 1,314.12 ± 103.6 CE (21.71 ± 3.7 generations ago) to 1,091.24 ± 180.88 CE (29.67 ± 6.46 generations ago) with Basque as the proxy for western sources or from 1,367.6 ± 100.52 CE (19.8 ± 3.59 generations) to 1,120.64 ± 238 CE (28.62 ± 8.5 generations) with French as the proxy for western sources. Other admixture times for Guizhou Huis are presented in Supplementary Table 23, which also evidenced their more recent admixture process, such as 20.04 ±1.18 generations for Guizhou Huis but 22.09 ± 4.57 generations for Boshu Huis in the Ami-French admixture model. As shown in Figure 7, we identified 47 pairs of west–east admixture sources fitted to all three included populations with different admixture times (Figure 7A): the admixture time estimated for Nanchong Hans was 31.79 ± 9.55 generations ago (1,087.86 ± 267.38 CE), followed by those for Boshu Huis at 23.64 ± 4.74 generations ago (1,315.97 ± 132.71) and Guizhou Huis at 20.88 ± 1.69 generations ago (1,393.29 ± 47.26 CE). We identified 31 pairs that fit the Hui populations but did not fit the Han Chinese populations, with an early admixture time for Boshu Huis of 23.12 ± 4.32 generations ago (1,330.53 ± 121.02 CE) and a recent admixture time for Guizhou Huis of 20.27 ± 1.58 generations ago (1,410.39 ± 44.34 Figure 7B). We also observed a complex genetic admixture process between northern and southern East Asians in both Han and Hui populations (Figure 7C, Supplementary Figure 32 and Supplementary Table 23). We should consider this estimated time as a general time that succeeded the first genetic admixture event due to continuous gene flow and admixture.

### Paternal/Maternal Founding Lineages and Sex-Biased Admixture

We assigned 109 tested mitochondrial genomes based on 4,198 maternal mitochondrial lineage-informative SNPs (MLISNPs) and 60 Y-chromosomal genomes (28 Huis and 32 Hans) based on 22,512 paternal Y-chromosomal lineage-informative SNPs (YLISNPs). Among 49 studied Hui people (Supplementary Table 24), we identified 36 maternal lineages with terminal lineage frequencies ranging from 0.0204 to 0.0816 (D4b2b, 4; R9b1b: 4). The maternal lineages B4a, B4i1, D4a, F2, M7b1a1, M8a3a1, and F2d were also identified at least twice in the Hui population. Twenty-eight Hui males were assigned to 13 different terminal paternal lineages with frequencies ranging from 0.0357 to 0.5357 (O2a2b1a2a1a3, 15). We also identified some samples with Siberian-dominant paternal lineages (C1b1a2b, C2c1a1, and C2c1b2b2~). R1b1a1a2a2c1a, which originated from ancient North Eurasians according to the study of ancient DNA, was also identified in Boshu Huis. However, on a more recent historical time scale, to further determine whether R1b occurred in Huis because of gene flow from the Yamnaya-related group or from more recent Central Asians after Islamization, denser ancient geographic and demographic sample collections and sequencing were needed. For the studied Han individuals, we identified 51 different maternal lineages with frequencies ranging from 0.0167 to 0.0500 (M7b1a1e1, 3). We obtained 25 terminal paternal lineages among 32 males with frequencies ranging from 0.0323 to 0.0968 (N1b2a2~, 3). We were also able to identify Siberian-derived lineages in Han populations (C2c1a1a1a, C2c1a2a2, C2c1a2b2, C2c1b2b2~, and Q1a1a1a1a~). Different from the western Eurasian or Siberian dominant Y-chromosomal haplogroups of Q1b2b1b2a-L330-F1893 and R1a identified in the Guizhou Hui population, more East Asian Y-chromosomal founding lineages were identified in Boshu Huis. To further validate the potential sex bias among Hui populations, we used *qpAdm* to estimate the ancestry composition based on genetic variations from autosomes (P_A_) and X chromosomes (P_X_, chrom:23). The sex-bias Z-score was calculated using the formula $Z_{SexBais}=\frac{P_{A}-P_{X}}{\sqrt{{SE_{A}}^{2}+{SE_{X}}^{2}}}$, in which SE_A_ and SE_X_ denoted the standard errors of the admixture proportions. Positive Z_SexBais_ scores suggested a male-driven admixture process due to autosomes possessing more western Eurasian ancestry than X chromosomes, and negative values supported a female-driven admixture process. As shown in Supplementary Table 25, we observed positive Z_sexBais_ scores in different three-way admixture models focused on both Guizhou Huis and Boshu Huis, which suggested a male-dominated admixture of western Eurasians and East Asians.

## Discussion

We provided newly generated genome-wide SNP data from Hui and Han Chinese individuals from western China and performed a comprehensive population genetic analysis to investigate their origin and admixture history. Shared genetic ancestry inferred from *f*_4_ statistics showed differentiated demographic histories between Huis and Hans: compared with geographically close Hans, Boshu Hui people possessed more ancestry related to modern western Eurasians (such as a *Z*-score for Mordovians of −53.986^*^SE in Supplementary Table 10) and Bronze Age steppe nomadic pastoralists (Sintashta: −58.585^*^SE in Supplementary Table 11). Fst- and outgroup-*f*_3_-based results further demonstrated the complex admixture histories of both Han and Hui people. Despite extensive studies focused on the genetic structure of Han Chinese, our results provide new insights into the Sichuan Hans. First, we found genetic homogeneity between our studied Nanchong Hans and other Han Chinese references in the PCA, Fst values, outgroup-*f*_3_ values, and model-based ADMIXTURE cluster patterns. *QpAdm*-based admixture models and a *qpGraph*-based phylogeny focusing on Nanchong Hans consistently demonstrated that Nanchong Hans have major ancestry from Northern East Asians and relatively minor ancestry from Southern East Asians. The identified model was consistent with the genetically attested model of the origins of East Asians based on both ancient DNA and modern genetic data (Wang M. et al., 2020). In addition, shared ancestry among geographically diverse Han Chinese populations is also supported by the linguistic affinity among Sino-Tibetans and their common origin from the middle and upper Yellow River basin in the Neolithic Yangshao period (Zhang et al., 2019). Additionally, we cannot exclude the scenario of their recent admixture because admixture signatures were also identified in the admixture-*f*_3_*(Central Han Chinese, Northern East Asians; Nanchong Hans)* calculation and historically documented migrations from southeastern China to western China. This needs to be further explored via historic ancient DNA from these areas. Second, in addition to the admixture signatures from Northern and Southern East Asians in the Nanchong Hans, we also identified a small amount of western Eurasian ancestry in this group, which was consistent with our recent findings for Shaanxi Hans and Gansu Hans (He et al., 2020; Yao et al., 2021). Due to the limited number of studied populations and the lack of temporally and geographically different ancient DNA from northwestern China, especially the lack of ancient DNA from the Hexi Corridor, we cannot clearly elucidate how this ancestry was introduced into Han Chinese. It may have been dispersed directly via western steppe pastoralists into East Asia or indirectly mediated by ancient southern Siberian populations during the interaction between northern Chinese and southern Siberian populations. Evidence of a genetic contribution from Hui to Han people, such as *f*_3_(Hui_Boshu, She_Fujian; Han_Nanchong) = −2.784^*^SE, also suggested the possibility that some of this western Eurasian signature may have been indirectly introduced via Hui people or other gene flow events into East Asia. In summary, modern northern Han Chinese, modern northwestern Han Chinese, and some modern western Han Chinese result from the admixture of ancient northern and southern sources within East Asia and some gene flow from western Eurasia.

Two well-known hypotheses have been proposed to explain the diffusion processes of certain cultures or population movements: the cultural diffusion model and the demic diffusion model (Wen et al., 2004). Many cases of the demic diffusion model have been reported in ancient and modern DNA studies, such as the Bantu expansion in Africa (Patin et al., 2017), the spread of barley/wheat agriculturalists into Europe from the Fertile Crescent in the Near East, and the broad dissemination of steppe nomadic pastoralists in the Eurasian steppe (Lazaridis et al., 2014; Narasimhan et al., 2019). In East Asia, ancient DNA studies have documented that the spread of rice/millet agriculture, as well as the corresponding language (agriculture–language–codispersal), is strongly correlated with the large-scale movement of people (Wang M. et al., 2020). Few cases of the cultural diffusion model have been recorded in relation to the formation of human populations, such as local cultural diffusion with no gene flow events or limited genetic influx. Wang et al. recently also found two cases of Afanasievo spread into Central Mongolia via the simple cultural diffusion model; however, the introduced early Bronze Age ancestry was quickly replaced via indigenous lineages (Wang M. et al., 2020). Previous historic and physical anthropology measurements suggested that the culture of Hui people (Muslim) and some of their ancestors spread into China via multiple migration events at different times in history (Yao et al., 2004). Findings from historic documents showed that Persian Silk Road travelers migrated into the southeastern coastal regions of China via the Maritime Silk Road, which formed their first wave. Documents focused on northeastern Chinese Hui people showed that they originated from Khorezmians who traveled to the area as merchants and soldiers in the Mongolian Empire and further mixed with local East Asians and other subsequent incoming Central Asians (https://en.wikipedia.org/wiki/Hui_people#cite_note-71). Currently, Hui people have unique cultural beliefs but use the same language as Han Chinese (Leslie, 1998; Wang et al., 2019). The formation processes for Chinese Hui people also raise interesting questions, including which models (cultural or demic) played a key role in their genomic formation.

To explore the historic demographic model of western Hui people, we performed one of the most comprehensive whole-genome-based population comparison studies between Chinese Hui (Boshu and Guizhou) and modern and ancient Eurasian populations to date. Our results suggested that neither hypothesis can simply fit the complexity of Huis' admixture history. Both East Asians and western Eurasians contributed to the Sichuan Hui people, with a major influence on assimilation from East Asians, which suggested that cultural diffusion played the dominant role in the Hui admixture history. We used different methods and western sources as potential ancestral sources in our comprehensive admixture model reconstructions and found a small amount of western Eurasian gene flow in the Hui people but more than that in geographically close Han Chinese. First, we observed western Eurasian ancestry in Chinese Huis in the model-based ADMIXTURE result, which was maximized in both western steppe pastoralists and modern Central Asian people and deviated toward western Eurasians in the PCA plots. Second, admixture-*f*_3_ statistics also revealed statistically significant admixture signatures when we used western Eurasians as one source and East Asians as the other source. Third, Chinese Hui individuals, especially Guizhou Hui individuals, shared more alleles with Bronze Age to contemporary western Eurasians in the symmetrical *f*_4_-based statistics than with East Asians. Fourth, we obtained a better-fit score with the *qpGraph*-based phylogenetic framework introducing western gene flow, and we also successfully fit the data with two-way or three-way *qpAdm*-based admixture models including one western Eurasian source. Finally, more ALDER-based admixture models can be obtained with western sources in Huis than Hans with admixture times ranging from 600 to 1,000 years ago. These western Eurasian genetic materials in Chinese individuals were also reported in other genetic investigations. Wang et al. also identified some Y-chromosomal lineages enriched in western Eurasian populations, such as western Eurasian-related lineages E, F, G, H, J, L, Q, R, and T (Wang et al., 2019). However, the dominant ancestry in Chinese Hui people was derived from East Asian populations, suggesting that large-scale genetic assimilation occurred between the original Hui ancestors and local East Asians. Y-chromosomal analysis in this study revealed a high proportion of East Eurasian lineages (O1 and O2) and a limited proportion of western Eurasian lineages. The Boshu Huis harbored more ancestry related to Northern East Asians than to Southern East Asians, further supporting the model in which the ancestors of Huis mixed with Northern East Asians. We also observed different demographic histories between the Boshu Huis and Guizhou Huis (Wang Q. et al., 2020). Thus, to better understand the genomic history of all geographically diverse Hui people, more genome-wide or sequence data should be collected.

The complex admixture and migration history in western Eurasians complicated the inference of the ancestry of Chinese Hui people. Recent ancient DNA studies have demonstrated that modern western Eurasians descended from three main ancestral populations: one related to local European hunter–gatherers, one related to incoming Anatolian farmers, and the other related to Early Bronze Age pastoralists from the Pontic-Caspian Steppe (Lazaridis et al., 2014). Paleolithic genomes from Siberia also illustrated that ancient western Eurasians made genetic contributions to modern Siberian, Central Asian, and Native American populations (Raghavan et al., 2014). Large-scale Holocene genomes from these areas demonstrated that the eastward migration of steppe herders contributed to the genomic formation of Iron Age people in Xinjiang and on the Mongolia Plateau (Ning et al., 2019; Jeong et al., 2020). Thus, the best-fitting models obtained in our *qpAdm* and *qpGraph* analyses (Supplementary Figures 22–30) may be explained by the indirect genetic influence of western Eurasian gene flow on Huis. The recent admixture time obtained from the ALDER analysis suggested that the potential direct ancestral sources of Chinese Huis are historic populations from Central Asia, which is consistent with documented history and the better-fitting Z-scores in the *qpGraph* models. Another possibility is that the ancestral sources of Huis' western genetic materials are complex, such as the genetically attested complex admixture history of Central Asians or Southern Asians (Narasimhan et al., 2019). One potential issue is that some different proposed ancestral sources were used in these analyses (*ALDER* and *qpAdm*) because of different datasets or the limitations of some analyses. However, the overlapping ancestral sources provided consistent admixture scenarios.

In addition to providing genetically documented knowledge of the Chinese Hui people, our findings raised some questions. The first is why some western Eurasian admixture signatures appeared or disappeared in Hui and Han populations when using different analysis methods. The limitation of the statistical algorithm can cause this, and other reasons, such as SNP ascertainment bias and the influence of overlapping SNPs or rare variants, should be further explored. Two other important raised questions are which is the major western admixture source for Chinese Huis and whether both Iranian barley farmer-related southwestern Eurasians and northern steppe pastoralists are ancestors of Chinese Huis. The fitted *qpGraph*-based models with different western Eurasian sources obtained in Supplementary Figures 22–30 revealed western Eurasian gene flow to Chinese Huis but cannot provide evidence that they were the direct ancestors of Huis due to ALDER-based findings and historically documented evidence, consistently suggesting that this western gene flow occurred. Thus, questions related to the plausible western Eurasian proxy, single, or multiple western sources and other topics must be explored in the future. In summary, population genomic studies based on a greater temporal transect of historic populations from Central Asia and Northwest China need to be conducted to provide more detailed temporal dynamics of the population transformation of Chinese Huis and provide a better direct western Eurasian source for Hui genomic formation.

## Conclusion

We generated genome-wide data from 49 Sichuan Hui and 60 Han individuals and merged them with all publicly available modern and ancient genomes to conduct one of the most comprehensive population genetic comparisons focused on testing the two contrasting hypotheses of the origin, diversification, migration and admixture of Hui people: the East Asian origin (cultural diffusion model) and western Eurasian origin (demic diffusion) hypotheses. We first identified significant genetic differentiation among geographically different Hui, as well as between Hui and adjacent Han populations, suggesting their different demographic structures. Findings based on the *f*-statistics demonstrated that both Huis and Hans possessed stronger Northern East Asian affinity, especially for late Neolithic-to-Iron-Age millet farmers from the middle Yellow River basin in the Central Plain, supporting the North China Origin hypothesis. The successfully fitted three-way admixture model with a newly identified small western Eurasian component and dominant northern and southern East Asian-like ancestry demonstrated that Sichuan Hui people represented a mix of minimal Central Asian genetic contribution along with their culture and massive Eastern Asian ancestry. The mixed populations adopted Muslim culture and ideas, which shaped the observed Hui gene pool composition, consistent with the cultural diffusion model playing a key role in the formation of the modern Hui population. In addition, the estimated admixture time based on the decay of admixture-introduced linkage disequilibrium revealed that the time of introduction of western Eurasian ancestry into the modern Hui population occurred ~600 years ago, consistent with the eastern-to-western Eurasian contact during the Tang, Song, and Yuan dynasties.

## Data Availability Statement

The original contributions presented in the study are included in the article/Supplementary Material, further inquiries can be directed to the corresponding author/s.

## Ethics Statement

The studies involving human participants were reviewed and approved by this project was inspected and approved by the Medical Ethics Committee of the North Sichuan Medical College. The patients/participants provided their written informed consent to participate in this study.

## Author Contributions

GH, YXL, GC, XZ, and MW conceived the idea for the study. YL, JY, YXL, RT, DY, YW, PW, SD, SZ, and HL performed or supervised wet laboratory work. GH, GC, XZ, YL, JY, YXL, RT, DY, YW, PW, SD, SZ, and HL analyzed the data. GH, YL, MW, and XZ wrote and edited the manuscript. All authors contributed to the article and approved the submitted version.

## Conflict of Interest

YXL was employed by company AnLan AI, Shenzhen, China. The remaining authors declare that the research was conducted in the absence of any commercial or financial relationships that could be construed as a potential conflict of interest.
