# Supplementary material for: Significant East Asian Affinity of the Sichuan Hui Genomic Structure Suggests the Predominance of the Cultural Diffusion Model in the Genetic Formation Process
Source: Front Genet. 2021 Jun 14;12:626710. doi: 10.3389/fgene.2021.626710 (PMC8237860; doi:10.3389/fgene.2021.626710)
Supplement: Supplementary file 2 [file Data_Sheet_2.pdf]

Supplementary Figures S1-32

Significant East Asian affinity of the Sichuan Hui genomic structure suggests the predominance of the cultural diffusion model in the genetic formation process

Yan Liu[1,*], Junbao Yang[1,*], Yingxiang Li[2,*], Renkuan Tang[3], Didi Yuan[3], Yicheng Wang[3], Peixin Wang[4], Shudan Deng[5], Simei Zeng[1], Hongliang Li[1], Gang Chen[6,#], Xing Zou[7,#], Mengge Wang[7,#], Guanglin He[7,8,*,#]

[1]School of Basic Medical Sciences, North Sichuan Medical College, Nanchong, Sichuan, 637100, China
[2]AnLan AI, Shenzhen, China
[3]Department of Forensic Medicine, College of Basic Medicine, Chongqing Medical University, Chongqing, 400331, China
[4]College of Medical Information, Chongqing Medical University, Chongqing, 400331, China
[5]School of Medical Imaging, North Sichuan Medical College, Nanchong, Sichuan, 637000, China
[6]Hunan Key Lab of Bioinformatics, School of Computer Science and Engineering, Central South University, Changsha, 410075, China
[7]Institute of Forensic Medicine, West China School of Basic Science and Forensic Medicine, Sichuan University, Chengdu, 610065, China
[8]Department of Anthropology and Ethnology, Institute of Anthropology, National Institute for Data Science in Health and Medicine, and School of Life Sciences, Xiamen University, Xiamen, 361005, China
*These authors contributed equally to this work and should be considered co-first authors.
#Corresponding author
Gang Chen
Affiliation: Hunan Key Lab of Bioinformatics, School of Computer Science and Engineering, Central South University, Changsha, 410075, China
Email: chengangcs@gmail.com
Xing Zou
Affiliation: Institute of Forensic Medicine, West China School of Basic Science and Forensic Medicine, Sichuan University
Email: forensiczx@163.com
Mengge Wang
Affiliation: Institute of Forensic Medicine, West China School of Basic Science and Forensic Medicine, Sichuan University
E-mail: wmg0126@163.com
Guanglin He
Affiliation: Department of Anthropology and Ethnology, Institute of Anthropology, National Institute for Data Science in Health and Medicine, Xiamen University, Xiamen, China.
E-mail: Guanglinhescu@163.com

Supplementary Figures S1-31

Figure S1. Geographic position of new collected Hui and Han people from Sichuan Province, Southwest China....4
Figure S2. Cross-Validation error value of model-based ADMIXTURE analysis. 4
Figure S3. Principal component analysis among Sino-Tibetan speakers and southern East Asians from Austronesian, Austroasiatic, Tai-Kadai and Hmong-Mien language families. 5
Figure S4. Principal component analysis among Eurasian populations....6
Figure S5A. Ancestry compositions of model-based ADMIXTURE analysis with the predefined ancestral sources ranging from two to twenty among modern and ancient Eurasian populations. Left part of the whole picture....7
Figure S5B. Ancestry compositions of model-based ADMIXTURE analysis with the predefined ancestral sources ranging from two to twenty among modern and ancient Eurasian populations. Right part of the whole picture. 8
Figure S6. Shared genetic drift between the Hui (A), Han (B) and modern Eurasian reference populations estimated via Outgroup-f_3-statistics. 9
Figure S7. Phylogenetic relationship among Eurasian populations. The neighbor-joining tree was constructed based on the invert f_3-based matrix ($1/f_3$). All included populations were coded by different color backgrounds....10
Figure S8. TreeMix results among 47 populations with three gene flow events. 11
Figure S9. Excess of sharing alleles between Boshu Hui and modern and ancient Eurasians showed genetic admixture based on the merged 1240K dataset. 12
Figure S10. Excess of sharing alleles between Boshu Hui and modern and ancient East Asians showed genetic admixture based on the merged Human Origin dataset....13
Figure S11. Results of f_4-statistics showed genomic relationship inferred from *f_4(Reference population1, reference population2; Hui_Boshu, Mbuti)* based on the merged Human Origin dataset. 14
Figure S12. Excess of sharing alleles between Nanchong Han and modern and ancient Eurasians. 15
Figure S13. Results of f_4-statistics showed genomic relationship inferred from *f_4(Reference population1, reference population2; Han_Nanchong, Mbuti)* based on the merged Human Origin dataset. 16
Figure S14. Excess of sharing alleles between Nanchong Han and modern and ancient Eurasians based on the merged Human Origin dataset. 17
Figure S15. *QpGraph-based admixture graphs illuminated a western gene flow related to Kazakhstan Andronovo into Boshu Hui can improve the fitness of the deep genomic model of Chinese Hui.* 18
Figure S16. *qpGraph*-based admixture graph showed the western Eurasian gene flow event related to Kalmykia Yamnaya into Boshu Hui....19
Figure S17. *qpGraph*-based admixture graph showed the western Eurasian gene flow event related to Russia_Sintashta_MLBA into Boshu Hui. 20
Figure S18. *qpGraph*-based admixture graph showed the western Eurasian gene flow event related to Russia Alan into Boshu Hui....21
Figure S19. *qpGraph*-based admixture graph showed the western Eurasian gene flow event related to Moldova Scythian into Boshu Hui. 22

Figure S20. *qpGraph*-based admixture graph showed the western Eurasian gene flow event related to French into Boshu Hui. 23
Figure S21. *qpGraph*-based admixture graph showed the western Eurasian gene flow event related to Sardinian into Boshu Hui. 24
Figure S22. *qpGraph*-based admixture graph showed the western Eurasian gene flow event related to Spanish into Boshu Hui. 25
Figure S23. *qpGraph*-based admixture graph showed the western Eurasian gene flow event related to Kazakhstan Andronovo into Guizhou Hui. 26
Figure S24. *qpGraph*-based admixture graph showed the western Eurasian gene flow event related to Kalmykia Yamnaya into Guizhou Hui. 27
Figure S25. *qpGraph*-based admixture graph showed the western Eurasian gene flow event related to Russia_Sintashta_MLBA into Guizhou Hui. 28
Figure S26. *qpGraph*-based admixture graph showed the western Eurasian gene flow event related to Russia Alan into Guizhou Hui. 29
Figure S27. *qpGraph*-based admixture graph showed the western Eurasian gene flow event related to Moldova Scythian into Guizhou Hui. 30
Figure S28. *qpGraph*-based admixture graph showed the western Eurasian gene flow event related to French into Guizhou Hui. 31
Figure S29. *qpGraph*-based admixture graph showed the western Eurasian gene flow event related to Sardinian into Guizhou Hui. 32
Figure S30. *qpGraph*-based admixture graph showed the western Eurasian gene flow event related to Spanish into Guizhou Hui. 33
Figure S31. *qpGraph*-based admixture graph showed the western Eurasian gene flow event related to Hungary Scythian into Guizhou Hui. 34
Figure S32. Admixture-introduced linkage disequilibrium (ALDER) based admixture time between different northern and southern or eastern and western ancestral sources based on 1240K dataset. 35

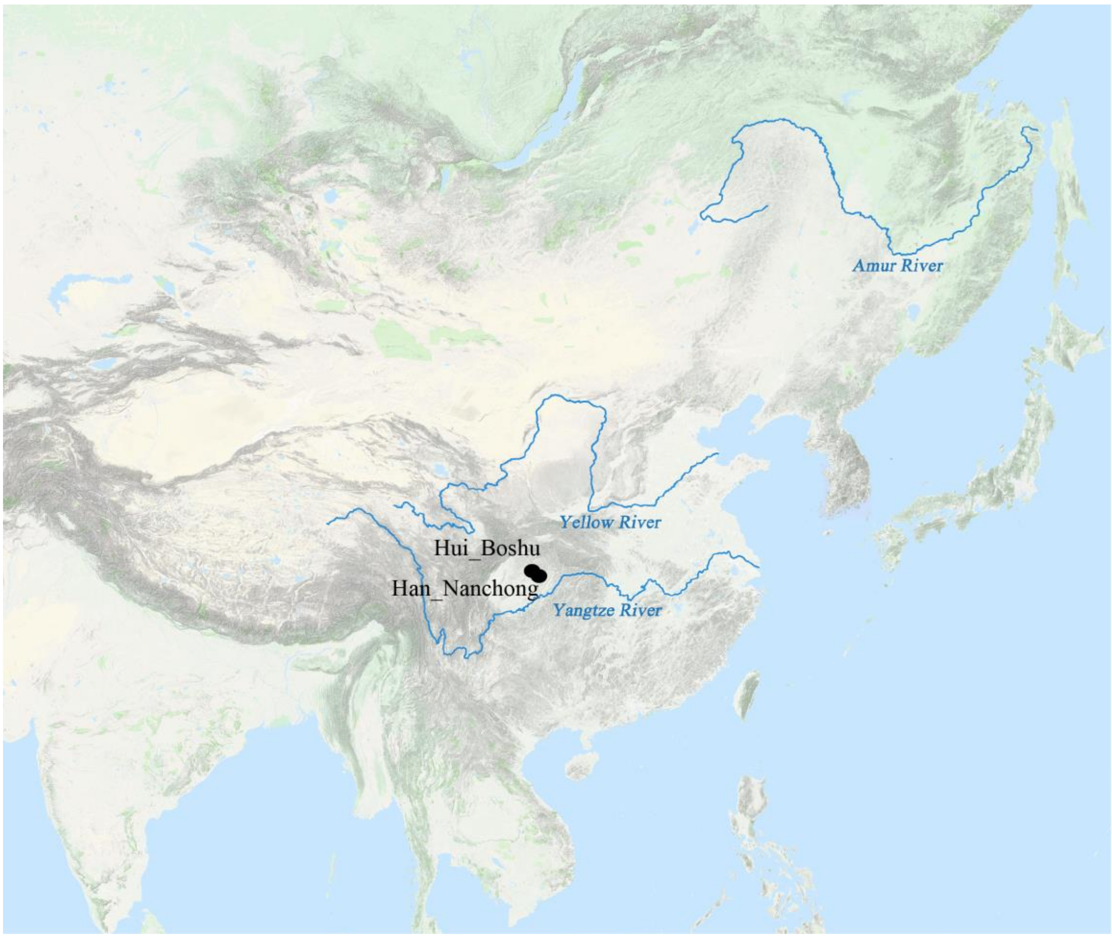

Figure S1. Geographic position of new collected Hui and Han people from Sichuan Province, Southwest China.

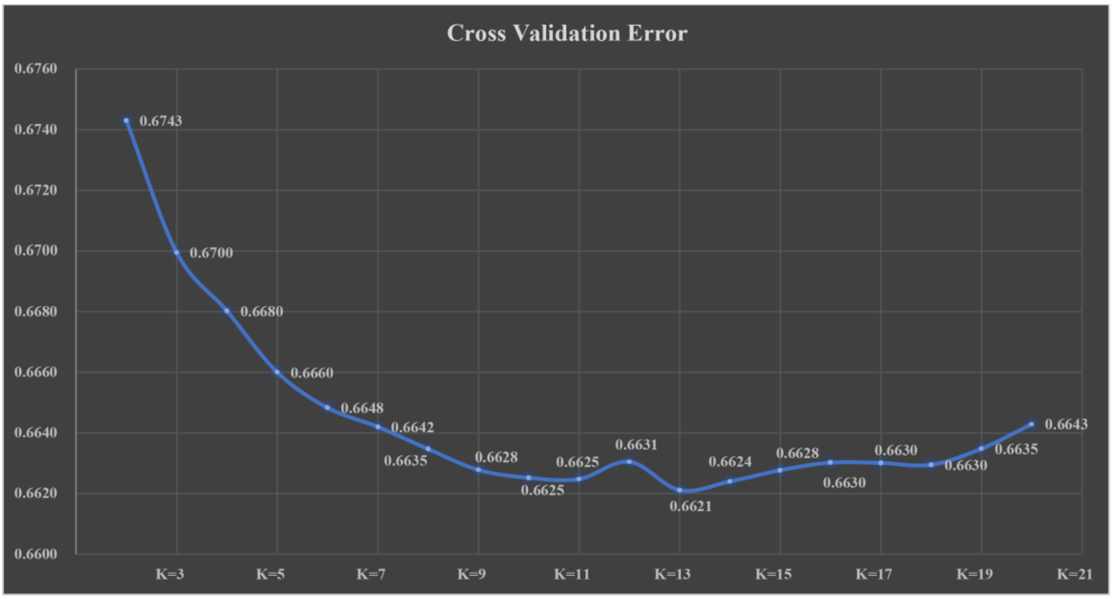

Figure S2. Cross-Validation error value of model-based ADMIXTURE analysis.

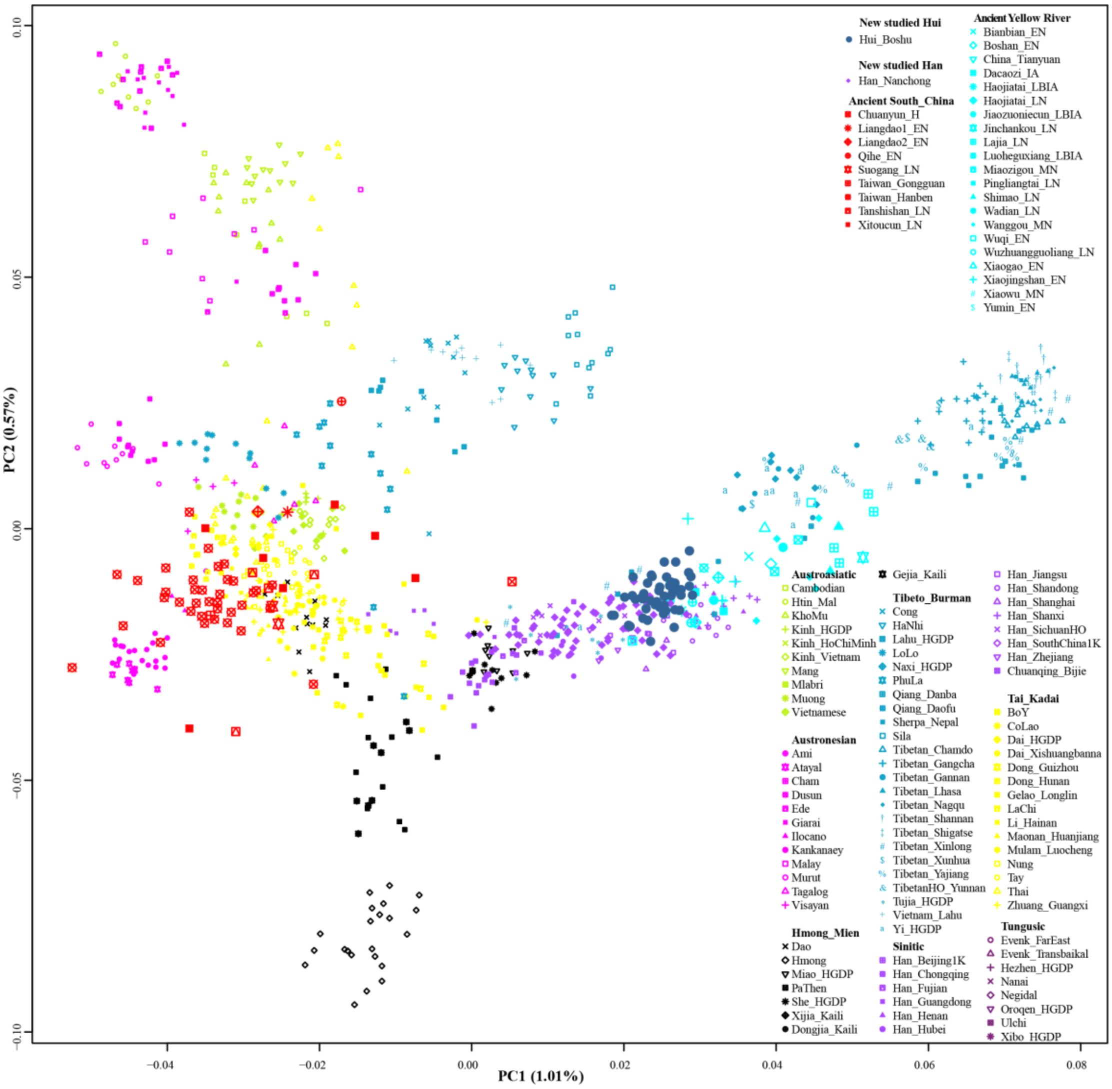

Figure S3. Principal component analysis among Sino-Tibetan speakers and southern East Asians from Austronesian, Austroasiatic, Tai-Kadai and Hmong-Mien language families.

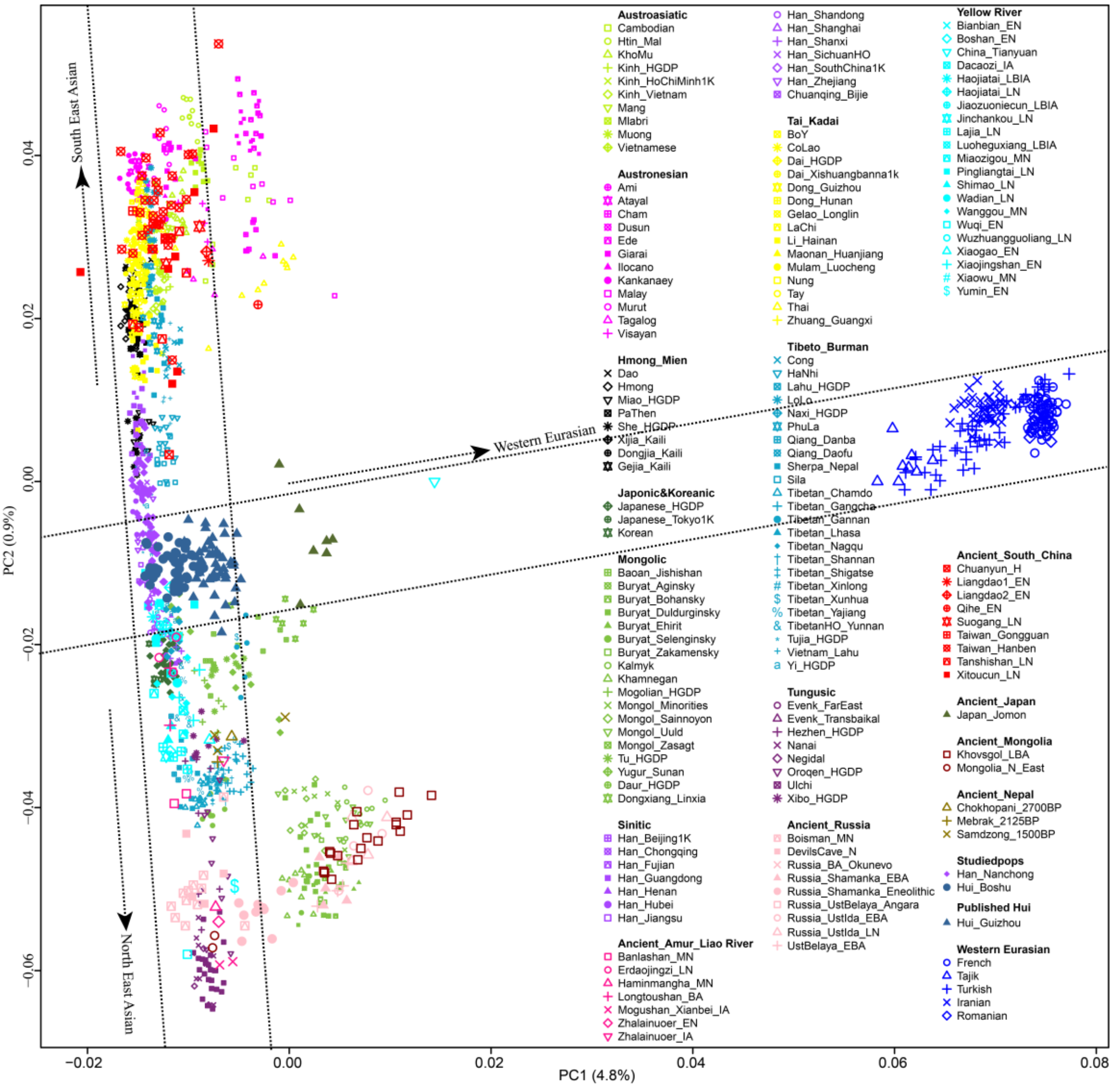

Figure S4. Principal component analysis among Eurasian populations.

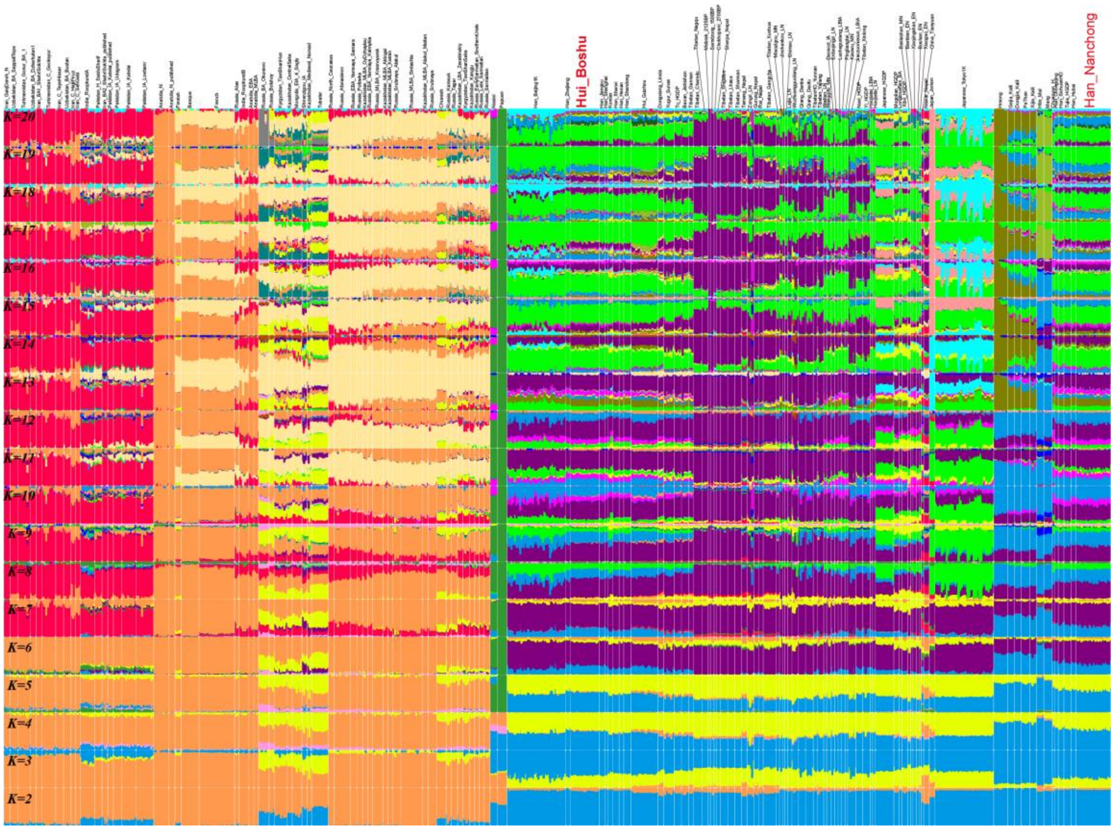

Figure S5A. Ancestry compositions of model-based ADMIXTURE analysis with the predefined ancestral sources ranging from two to twenty among modern and ancient Eurasian populations. Left part of the whole picture.

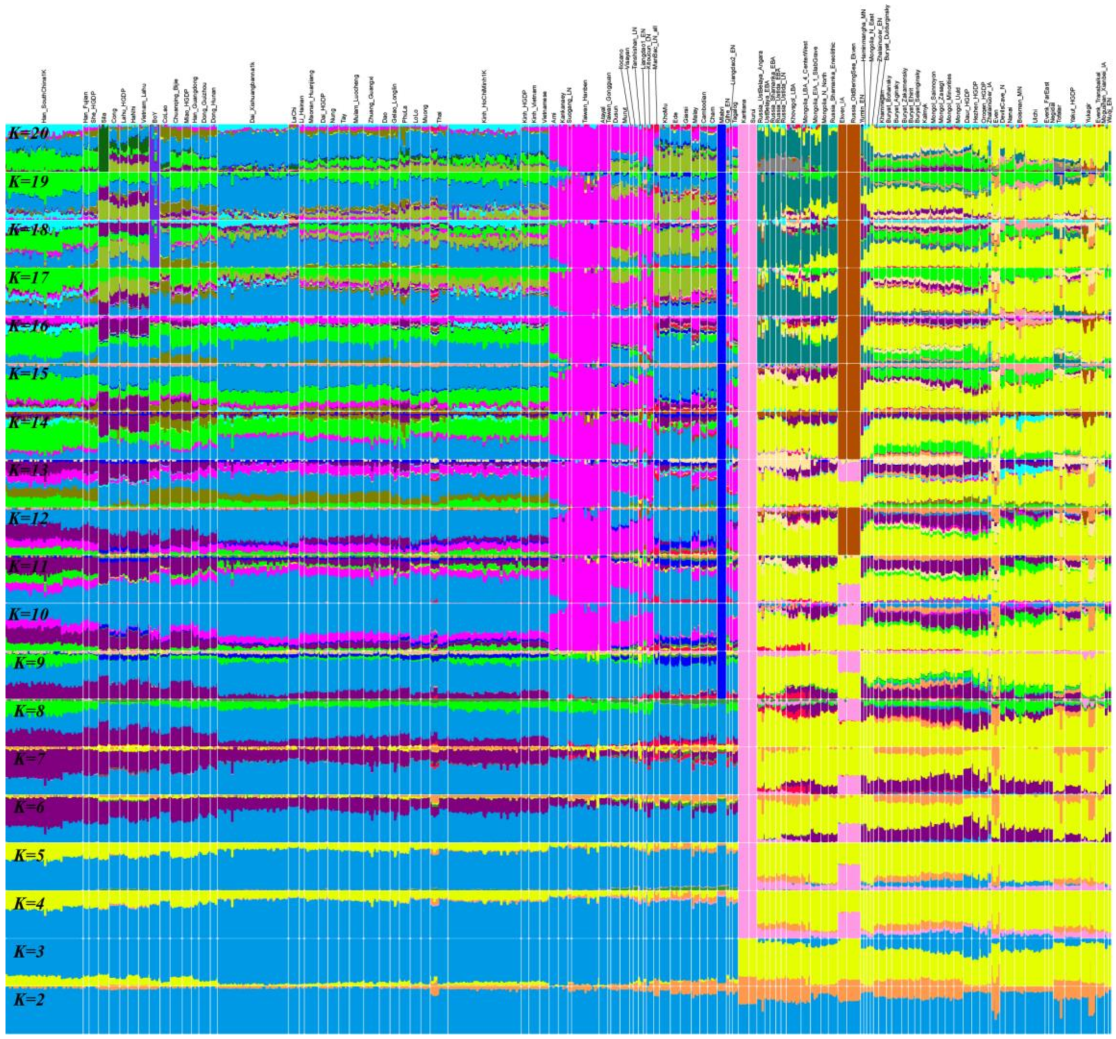

Figure S5B. Ancestry compositions of model-based ADMIXTURE analysis with the predefined ancestral sources ranging from two to twenty among modern and ancient Eurasian populations. Right part of the whole picture.

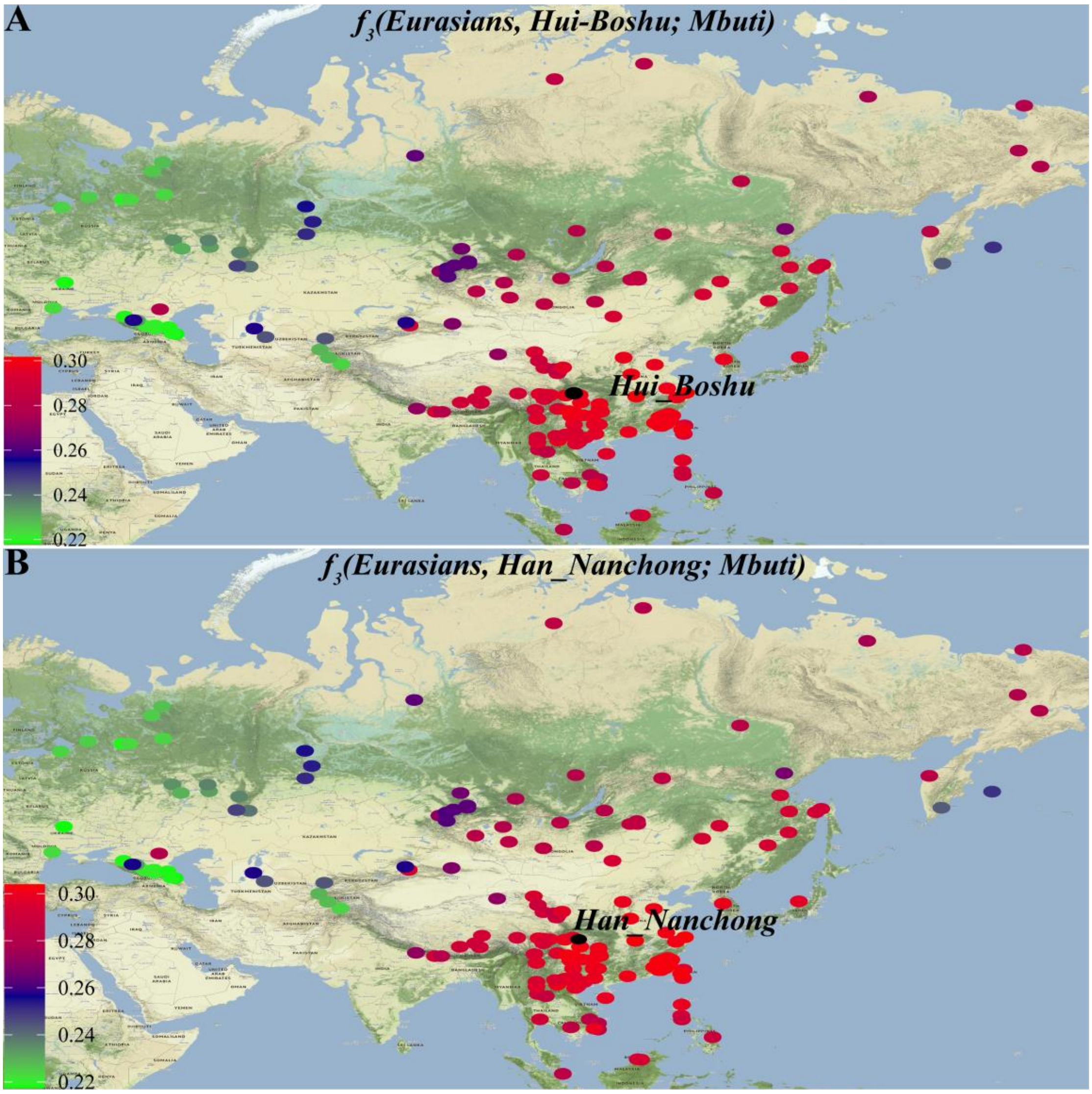

Figure S6. Shared genetic drift between the Hui (A), Han (B) and modern Eurasian reference populations estimated via Outgroup-f_3-statistics.

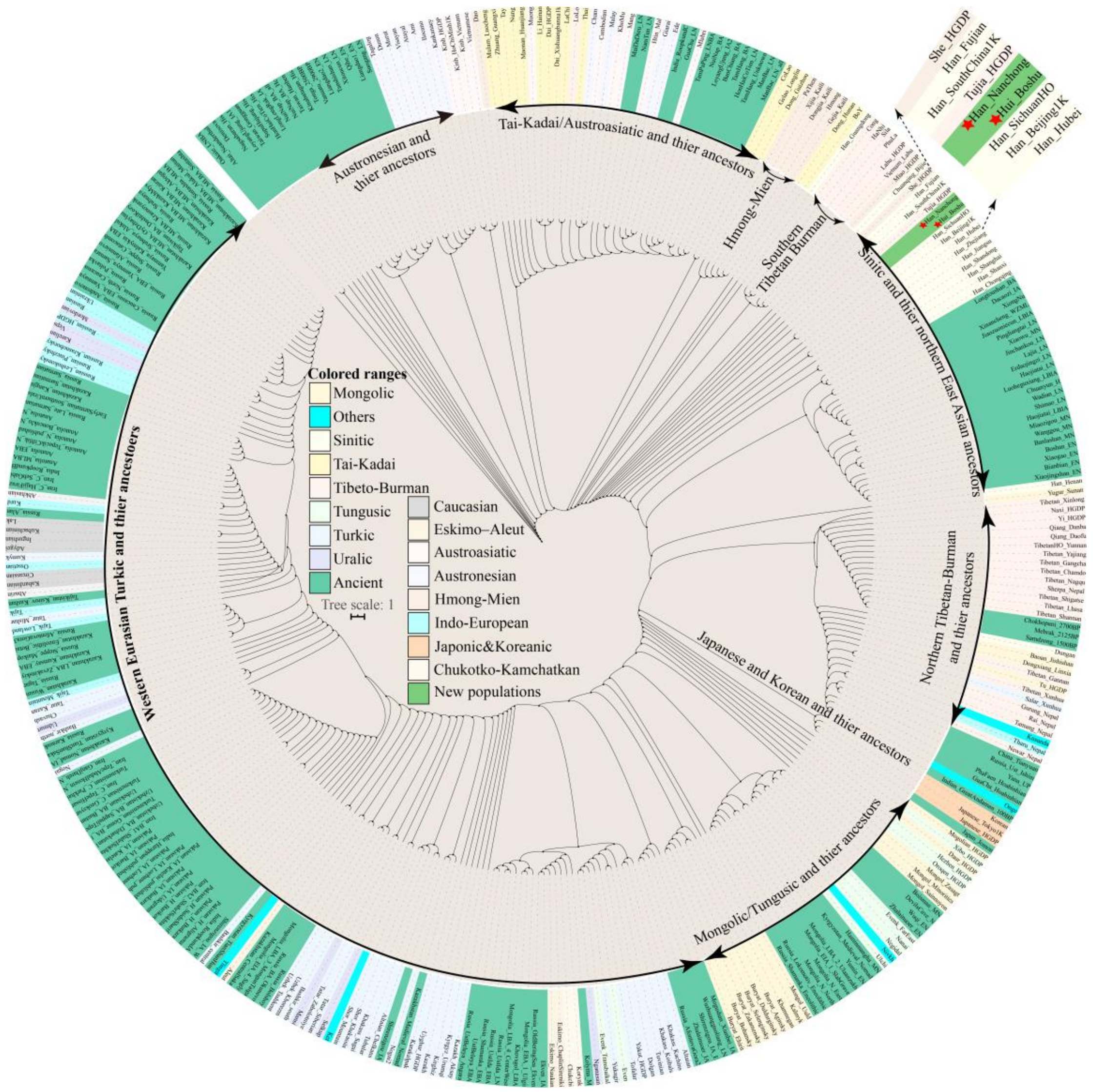

Figure S7. Phylogenetic relationship among Eurasian populations. The neighbor-joining tree was constructed based on the invert *f*$_3$-based matrix (1/*f*$_3$). All included populations were coded by different color backgrounds.

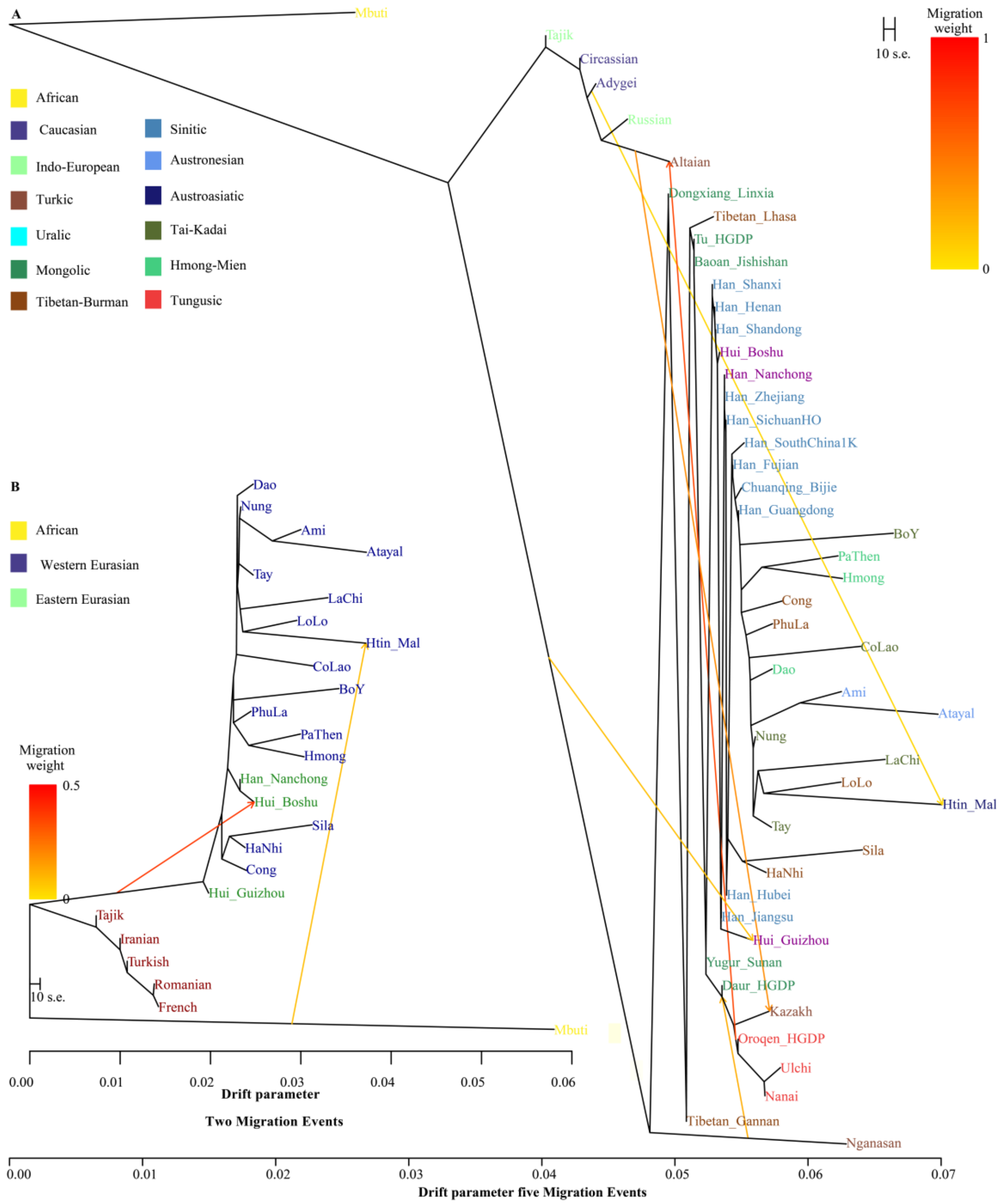

Figure S8. TreeMix results among 47 populations with three gene flow events.

(A). The left upper heatmap showed the residual matrixes and (B) the right tree showed the population splits and admixture events. Populations were colored according to their language family categories.

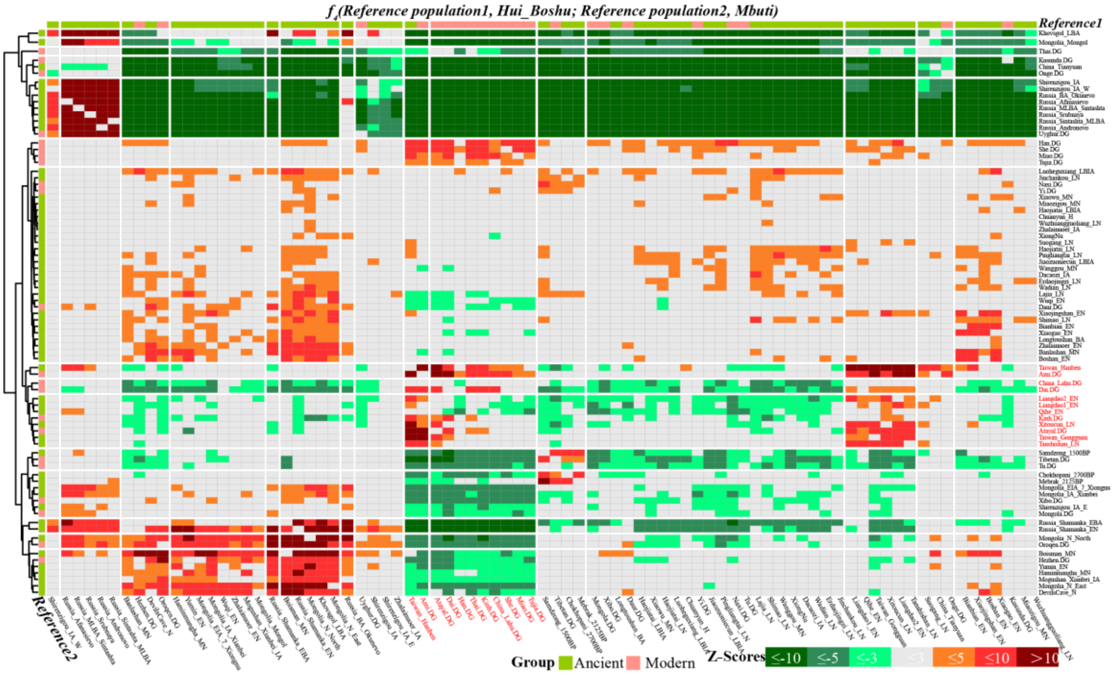

Figure S9. Excess of sharing alleles between Boshu Hui and modern and ancient Eurasians showed genetic admixture based on the merged 1240K dataset.

Affinity-f_4-statistics in the form *f_4(Reference population1, Hui_Boshu; reference population2, Mbuti)* to explore the genetic continuity and admixture between potential ancestral sources and Boshu Hui based on the merged 1240K dataset. The red color denoted the third population (Bottom population lists) shared more derived alleles with the first population (Right population lists) compared with the second one (Boshu Hui). And the green color denoted the third population shared more alleles with the Boshu Hui relative to the first one. Tree was constructed based on the f_4 matrix and red color-coded populations were possible ancestral sources.

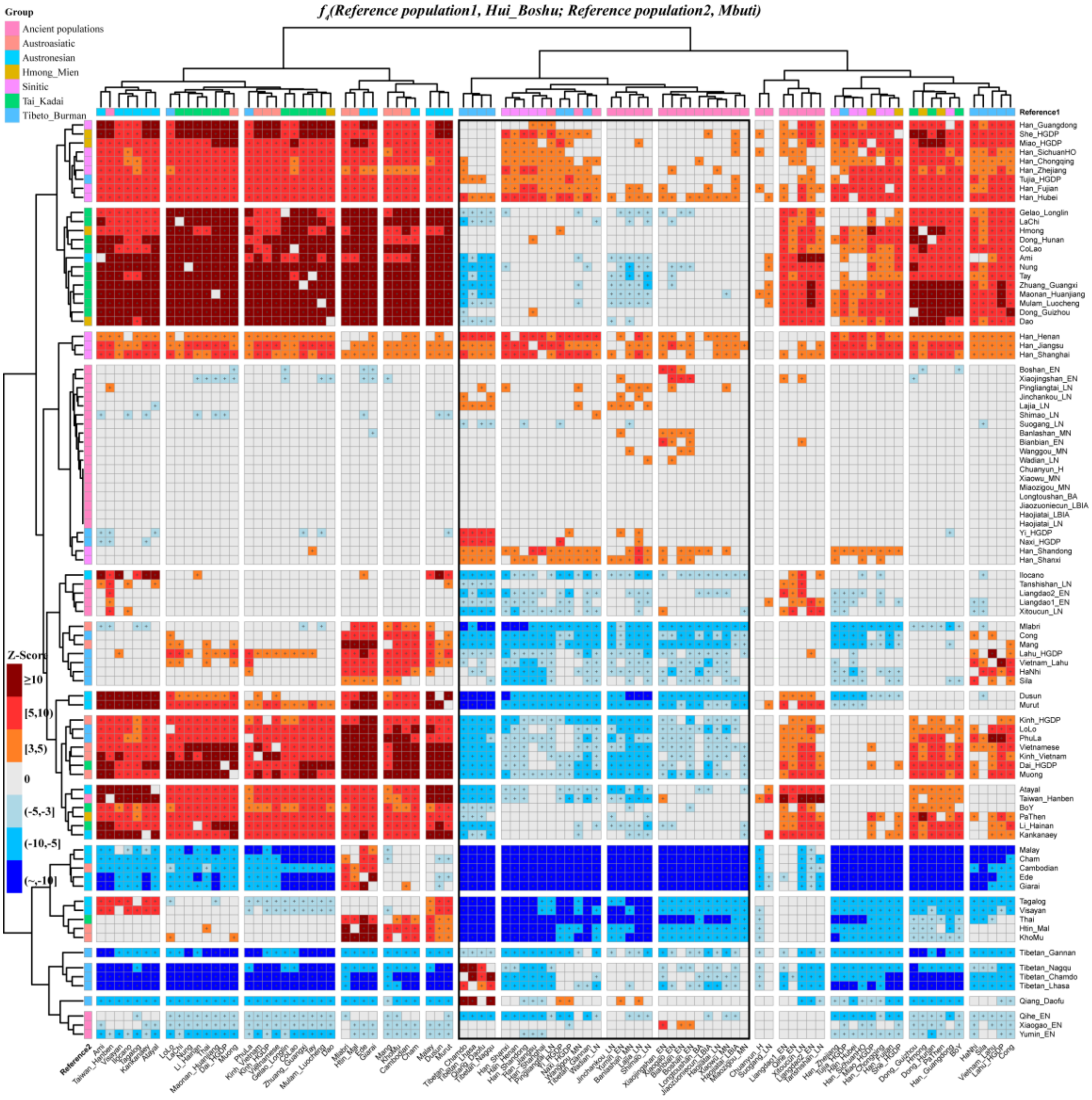

Figure S10. Excess of sharing alleles between Boshu Hui and modern and ancient East Asians showed genetic admixture based on the merged Human Origin dataset.

Affinity-f_4-statistics in the form *f_4(Reference population1, Hui_Boshu; reference population2, Mbuti)* to explore the genetic continuity and admixture between potential ancestral sources and Boshu Hui based on the merged 1240K dataset. The red color denoted the third population (Bottom population lists) shared more derived alleles with the first population (Right population lists) compared with the second one (Boshu Hui). And the green color denoted the third population shared more alleles with the Boshu Hui relative to the first one. Tree was constructed based on the f_4 matrix and red color-coded populations were possible ancestral sources.

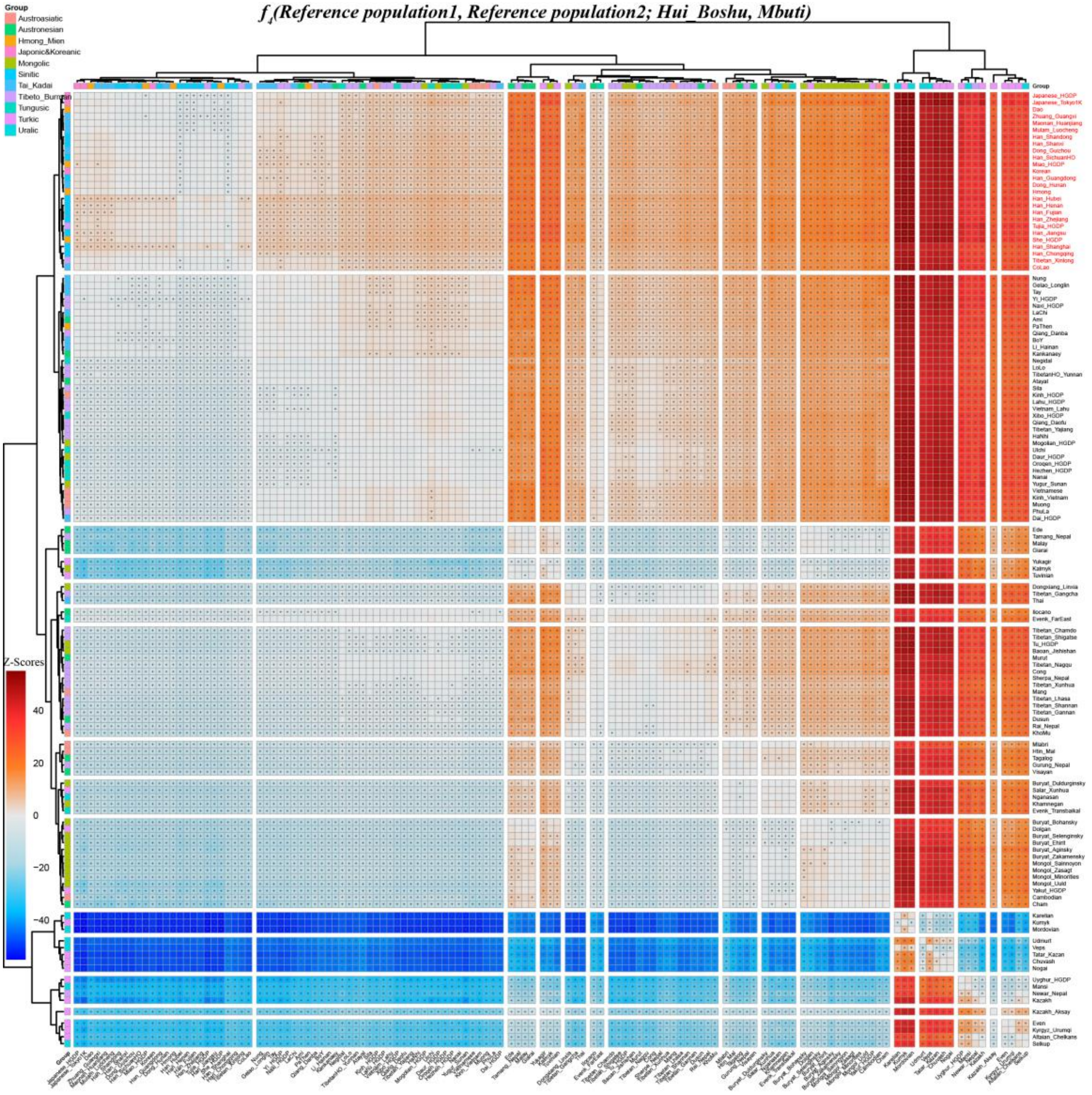

Figure S11. Results of f_4-statistics showed genomic relationship inferred from *f_4(Reference population1, reference population2; Hui_Boshu, Mbuti)* based on the merged Human Origin dataset.

Reference population1 was the right population list, and reference population2 was the bottom population list. Statistically significant *f*-statistics were marked as "+". Tree was constructed based on the f_4 matrix and red color-coded populations were possible ancestral sources. Red color showed positive f_4 values, which suggested that Boshu Hui possessed significantly more allele sharing of reference popultion1 (Right population lists) related to reference population2 (Bottom population lists). Blue color showed negative f_4 values, which suggested that Boshu Hui harbored excess sharing alleles with reference population2 (Bottom population lists) relative to reference popultion1 (Right population lists). Symmetrical f_4-statistics in the form *f_4(Reference population1, reference population2; Hui_Boshu, Mbuti)* to test excess allele sharing between Boshu Hui and northern East Asians and all Sinitic speakers (Right population lists, coded by red color) relative to other Eurasian reference populations.

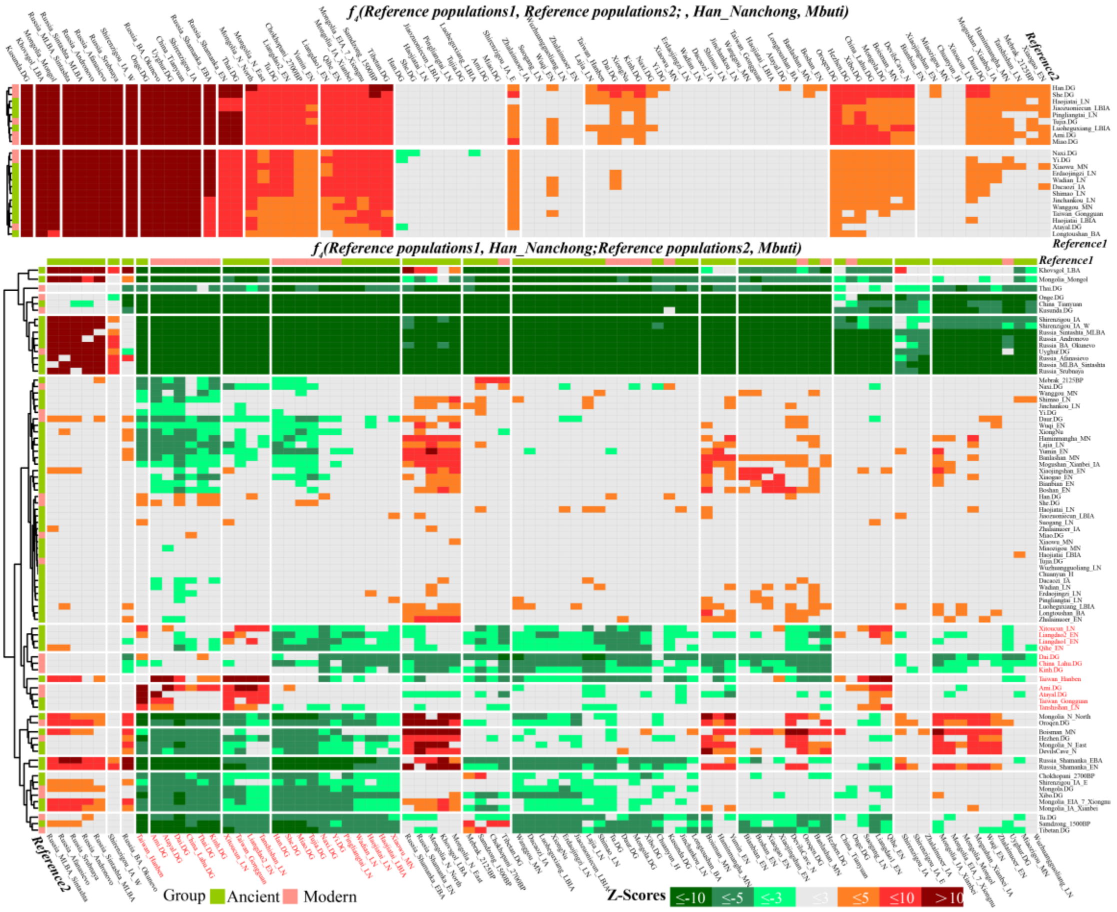

Figure S12. Excess of sharing alleles between Nanchong Han and modern and ancient Eurasians.

Symmetrical f_4-statistics in the form f_4*(Reference population1, reference population2; Han_Nanchong, Mbuti)* to test the genetic affinity between Eurasian reference populations (**A**). Affinity-f_4-statistics in the form f_4*(Reference population1, Han_Nanchong; reference population2, Mbuti)* to explore the genetic continuity and admixture between potential ancestral sources and Boshu Hui (**B**). The red color denoted the third population shared more derived alleles with the first population compared with the second one. And the green color denoted the third population shared more alleles with the second one relative to the first one. Tree was constructed based on the f_4 matrix and red color-coded populations were possible ancestral sources.

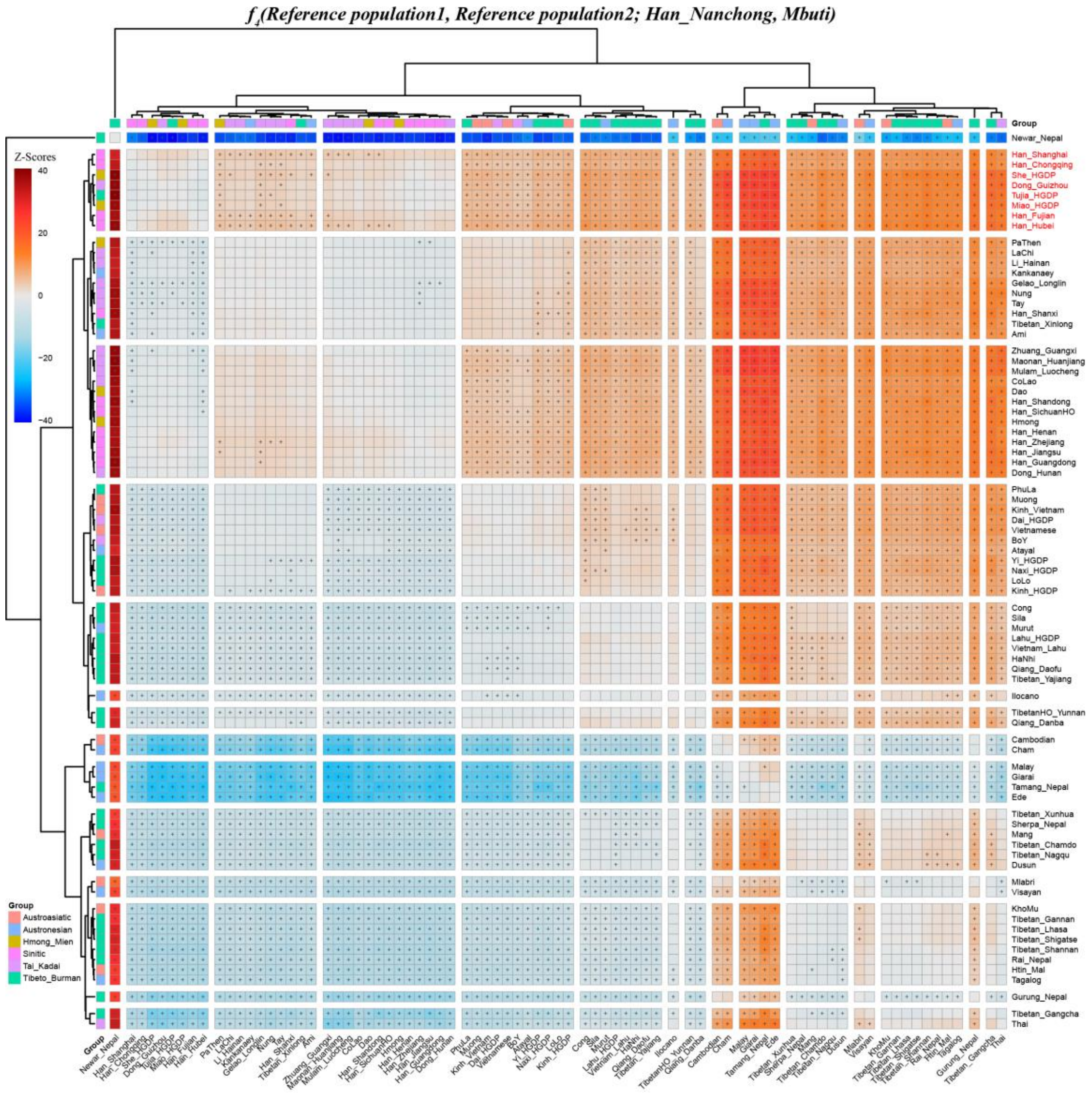

Figure S13. Results of f_4-statistics showed genomic relationship inferred from *f_4(Reference population1, reference population2; Han_Nanchong, Mbuti)* based on the merged Human Origin dataset.

Reference population1 was the right population list, and reference population2 was the bottom population list. Blue color showed negative f_4 values, which suggested that Nanchong Han harbored excess sharing alleles with reference population2 (Bottom population lists) relative to reference popultion1 (Right population lists). Red color showed positive f_4 values, which suggested that Nanchong Han possessed significantly more allele sharing of reference popultion1 (Right population lists) related to reference population2 (Bottom population lists). Statistically significant f-statistics were marked as "+". Tree was constructed based on the f_4 matrix and red color-coded populations were possible ancestral sources. Symmetrical f_4-statistics in the form *f_4(Reference population1, reference population2; Han_Nanchong, Mbuti)* to test excess allele sharing between Nanchong Han and northern East Asians (Right population lists) relative to other Eurasian reference populations.

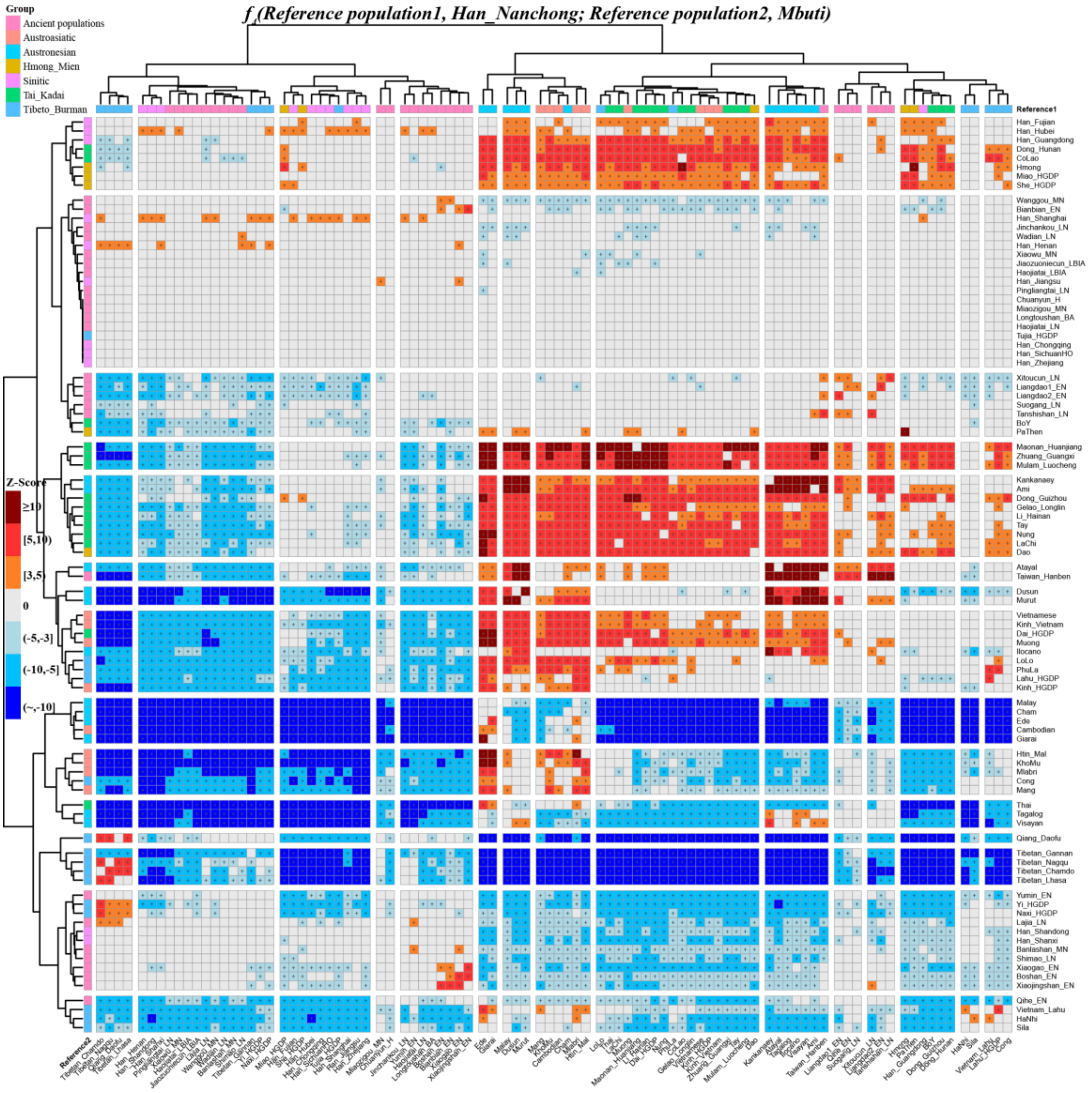

Figure S14. Excess of sharing alleles between Nanchong Han and modern and ancient Eurasians based on the merged Human Origin dataset.

Affinity-f_4-statistics in the form *f_4(Reference population1, Han_Nanchong; reference population2, Mbuti)* to explore the genetic continuity and admixture between potential ancestral sources and Nanchong Han based on the merged Human Origin dataset. The red color denoted the third population (Bottom population lists) shared more derived alleles with the first population (Right population lists) compared with the second one (Nanchong Han). And the green color denoted the third population shared more alleles with the Nanchong Han relative to the first one. Statistically significant *f*-statistics were marked as “+”. The tree was constructed based on the f_4 matrix.

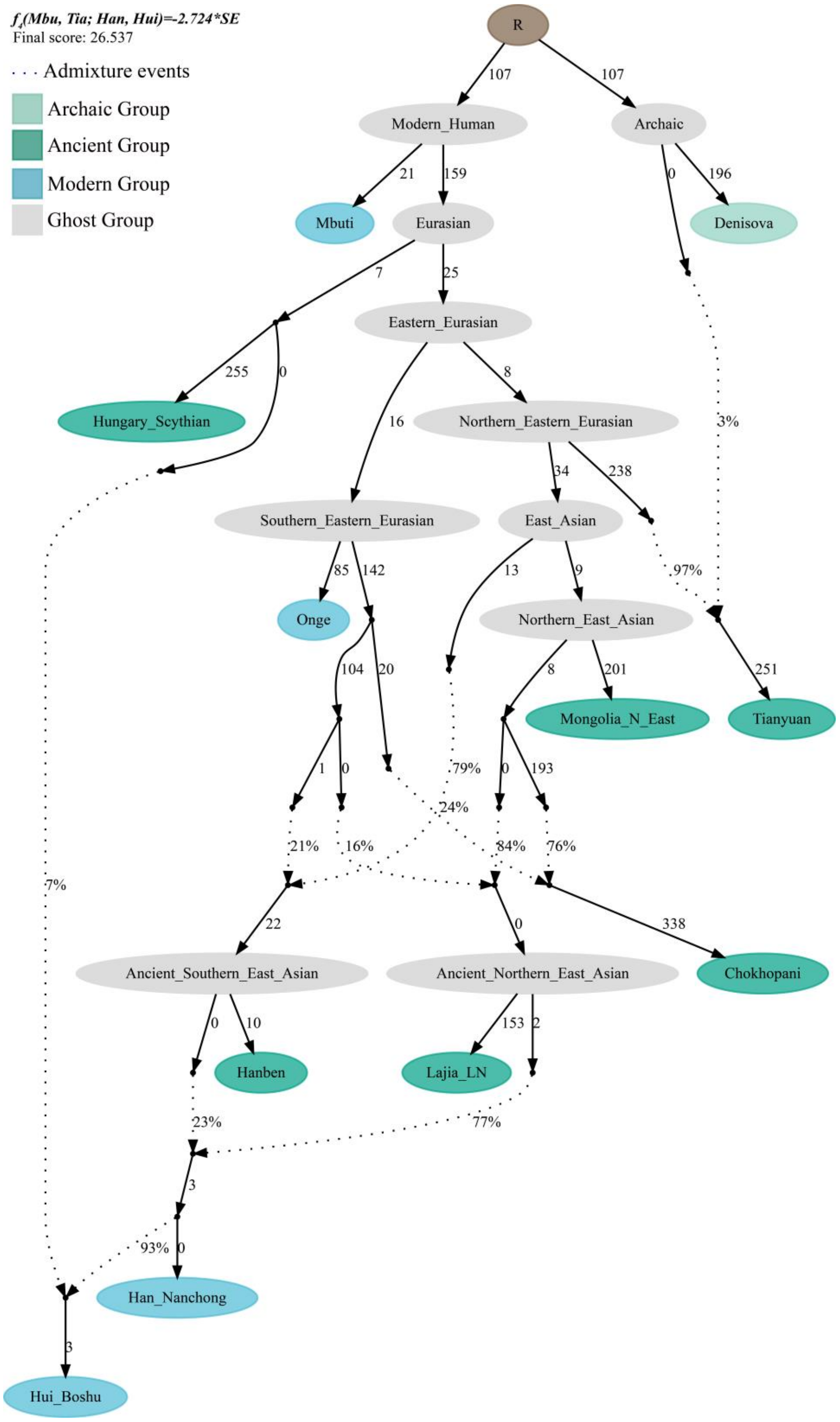

Figure S15. ***QpGraph-based admixture graphs illuminated a western gene flow related to Kazakhstan Andronovo into Boshu Hui can improve the fitness of the deep genomic model of Chinese Hui.***

Branch length was marked with the f2 shared drift distance (1000 times). Admixture events were denoted as dotted line. Admixture proportion was marked along the dotted line. EBA: Early Bronze Age; LN, Late Neolithic; N, Neolithic.

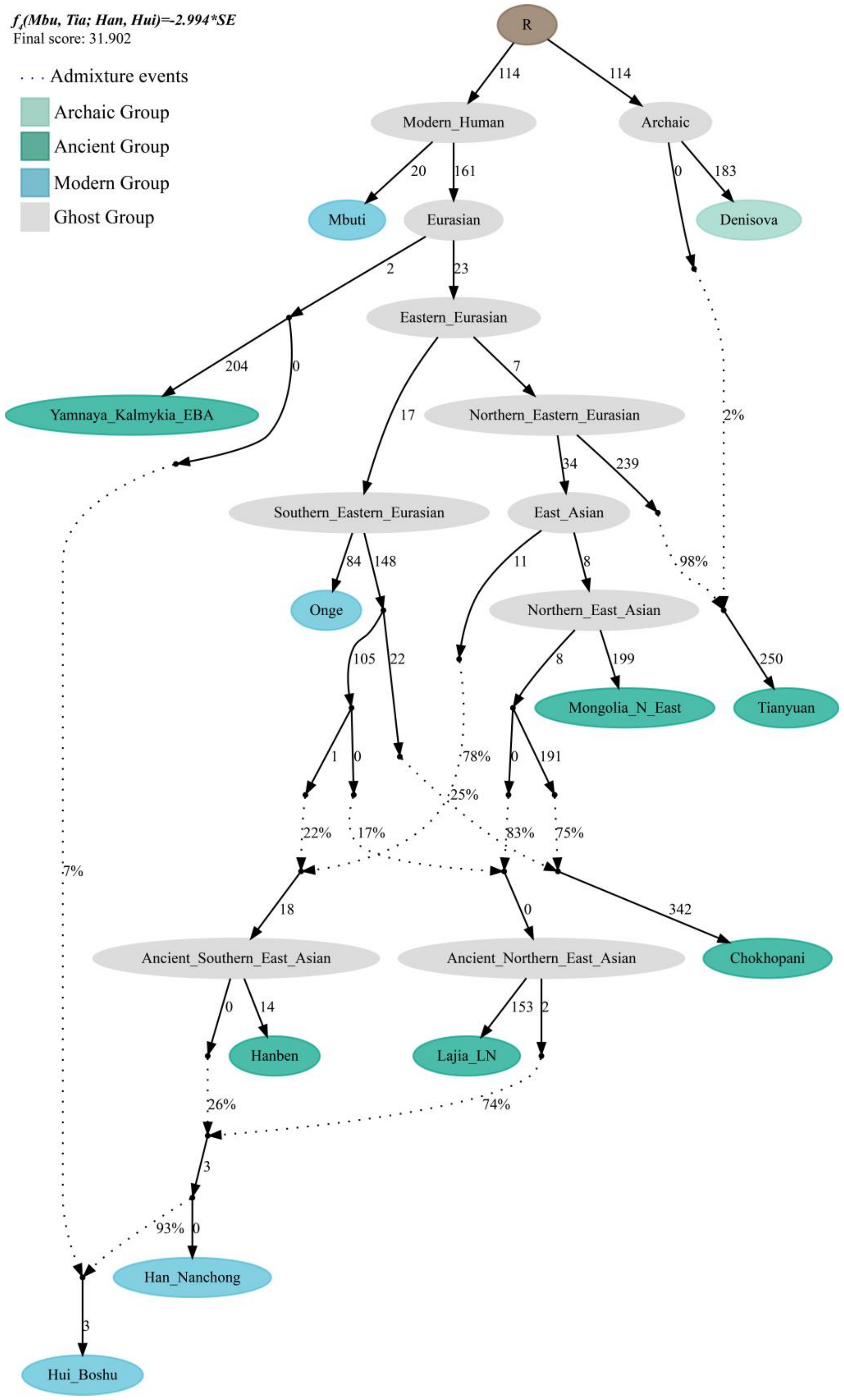

Figure S16. *qpGraph*-based admixture graph showed the western Eurasian gene flow event related to Kalmykia Yamnaya into Boshu Hui.

Branch length was marked with the f_2 shared drift distance (1000 times). Admixture events were denoted as dotted line. Admixture proportion was marked along the dotted line. EBA: Early Bronze Age; LN, Late Neolithic; N, Neolithic.

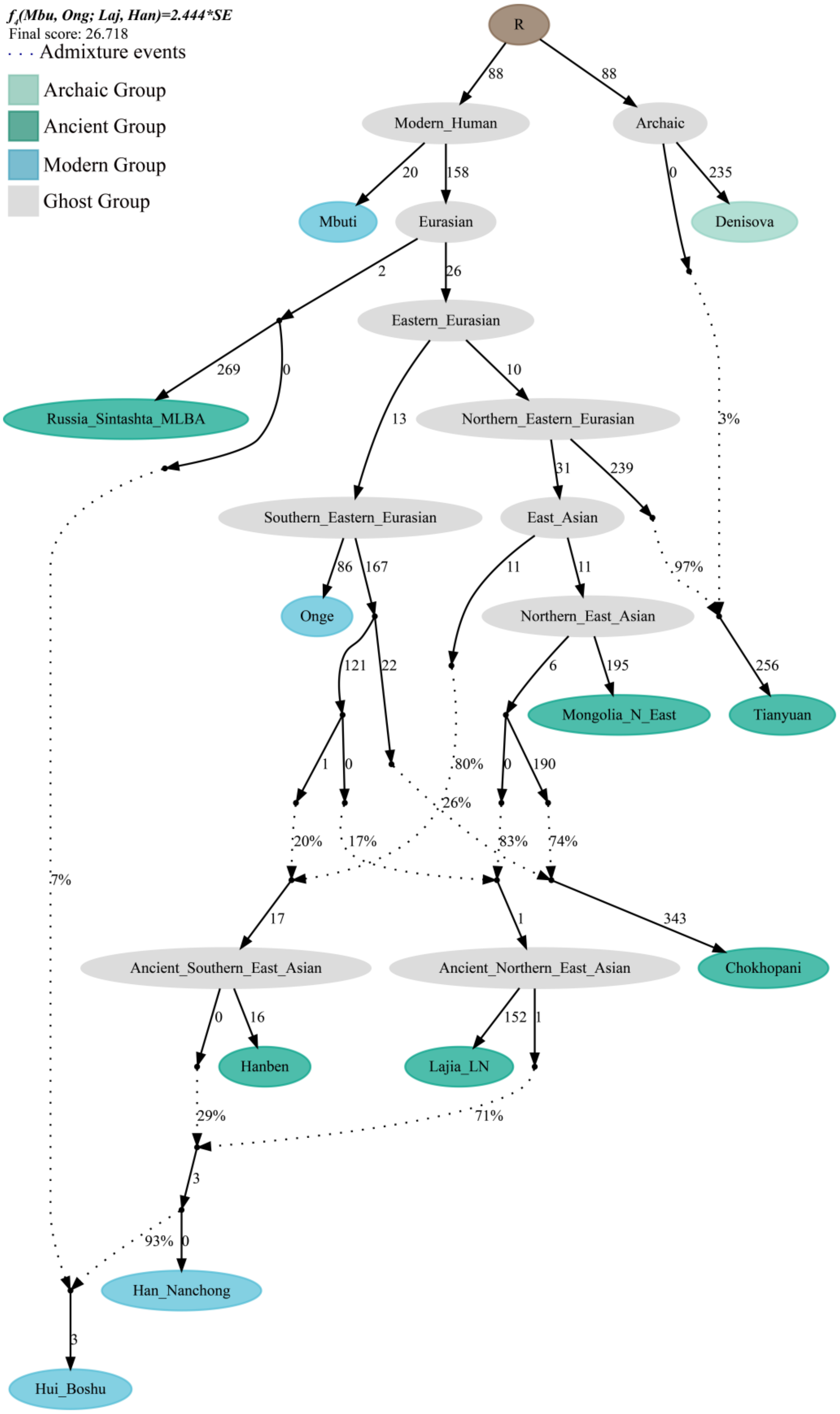

Figure S17. *qpGraph*-based admixture graph showed the western Eurasian gene flow event related to Russia_Sintashta_MLBA into Boshu Hui.

Branch length was marked with the f_2 shared drift distance (1000 times). Admixture events were denoted as dotted line. Admixture proportion was marked along the dotted line. MLBA: Middle-Late Bronze Age; LN, Late Neolithic; N, Neolithic.

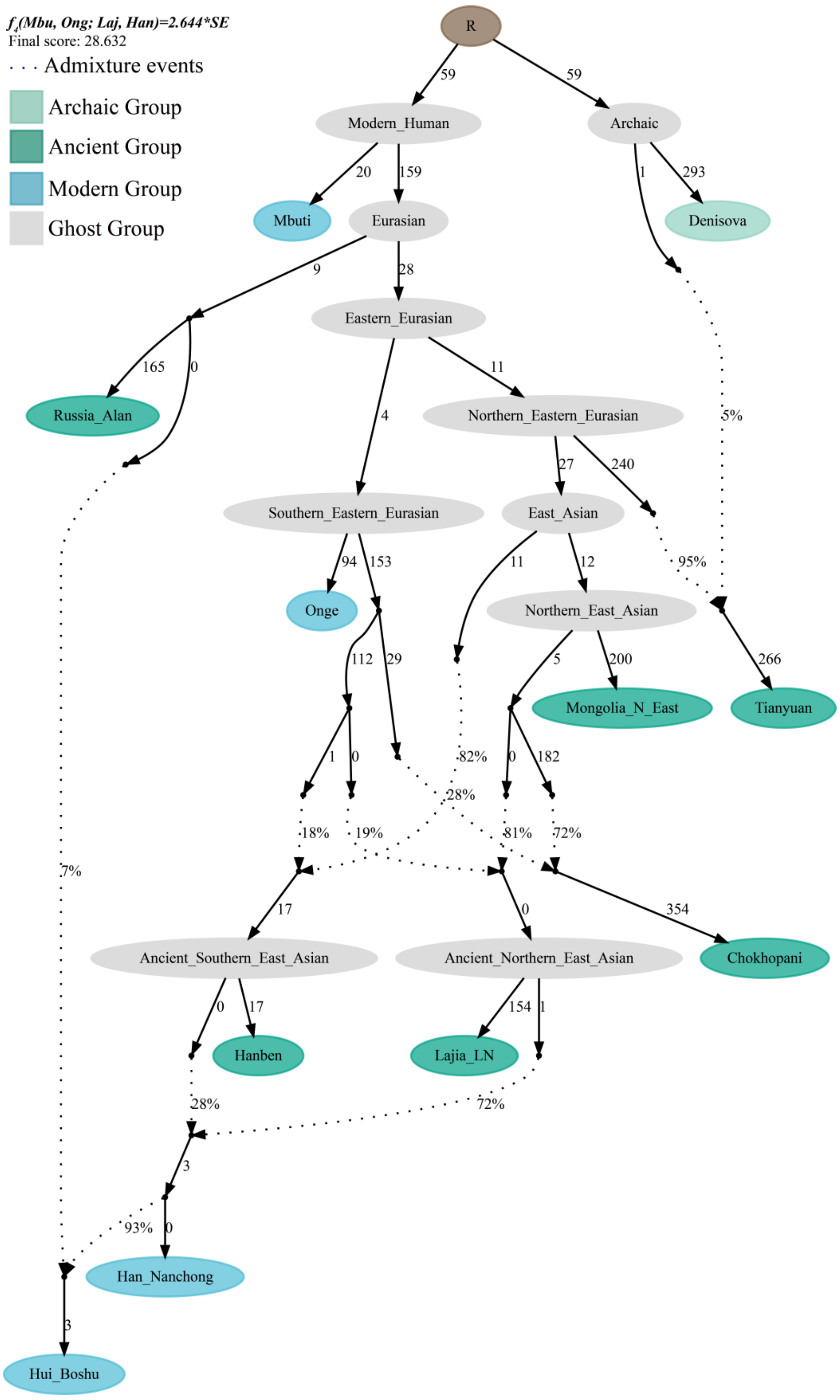

Figure S18. ***qpGraph*-based admixture graph showed the western Eurasian gene flow event related to Russia Alan into Boshu Hui.**

Branch length was marked with the f_2 shared drift distance (1000 times). Admixture events were denoted as dotted line. Admixture proportion was marked along the dotted line. LN, Late Neolithic; N, Neolithic.

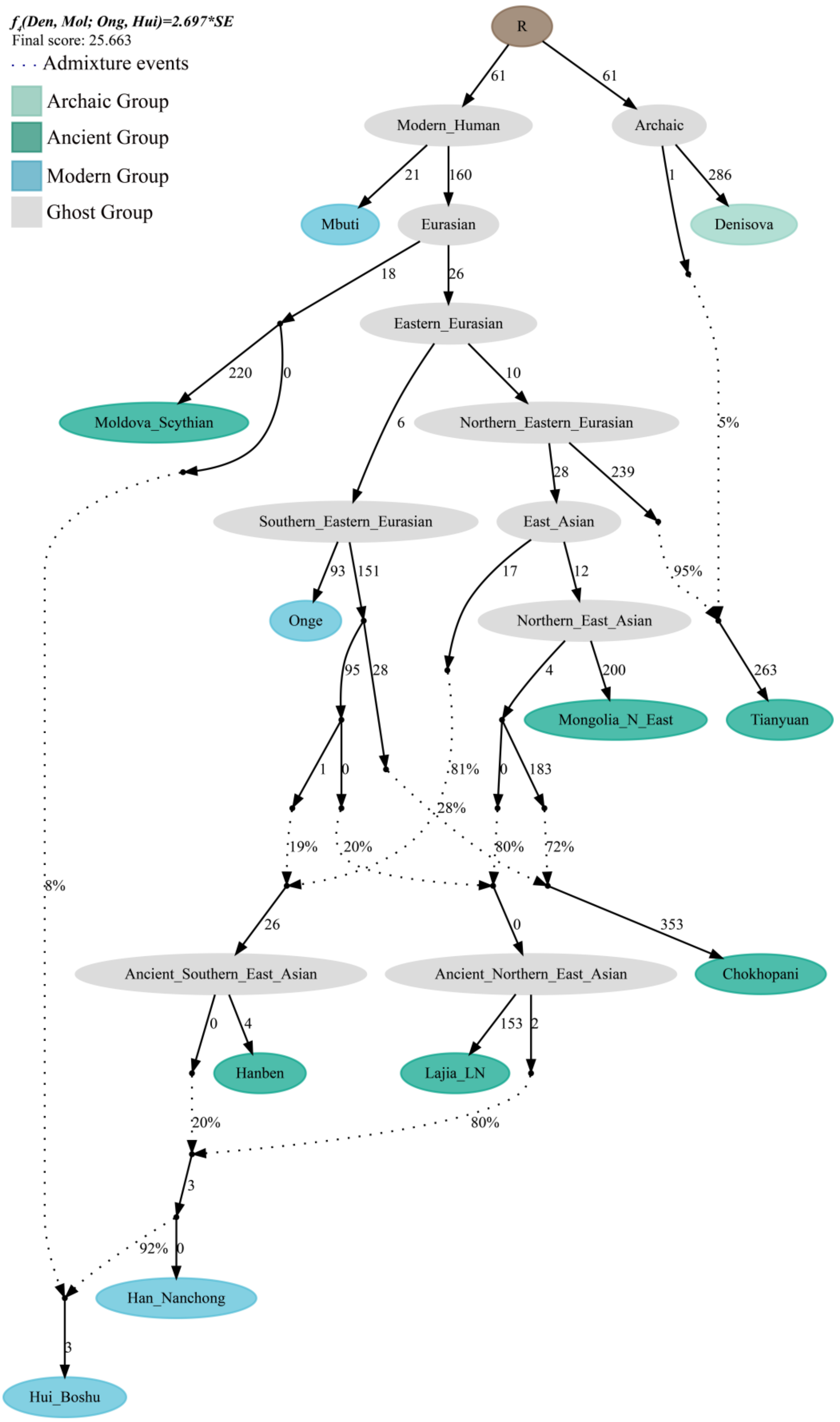

Figure S19. *qpGraph*-based admixture graph showed the western Eurasian gene flow event related to Moldova Scythian into Boshu Hui.

Branch length was marked with the f_2 shared drift distance (1000 times). Admixture events were denoted as dotted line. Admixture proportion was marked along the dotted line. LN, Late Neolithic; N, Neolithic.

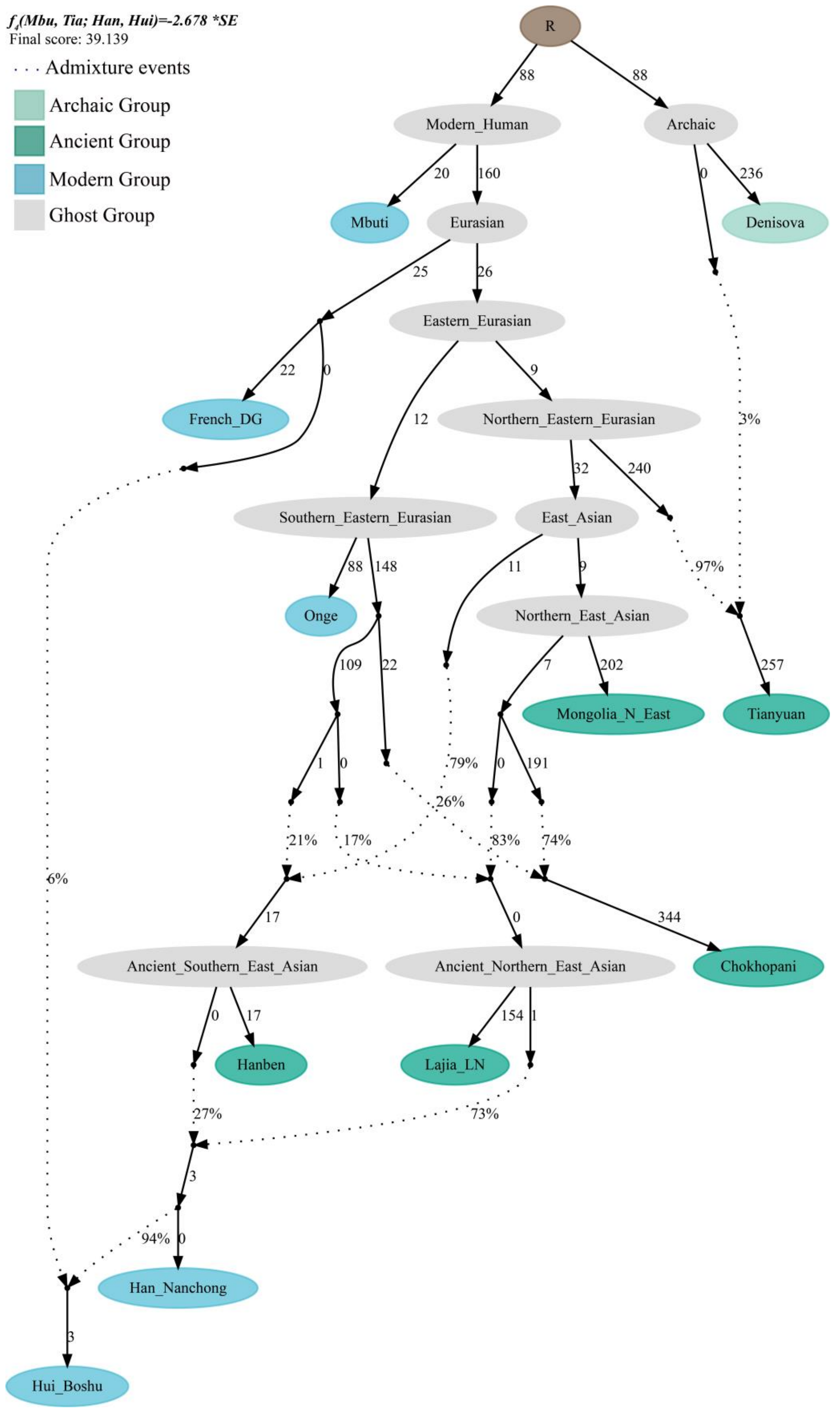

Figure S20. ***qpGraph*-based admixture graph showed the western Eurasian gene flow event related to French into Boshu Hui.**

Branch length was marked with the f_2 shared drift distance (1000 times). Admixture events were denoted as dotted line. Admixture proportion was marked along the dotted line. LN, Late Neolithic; N, Neolithic.

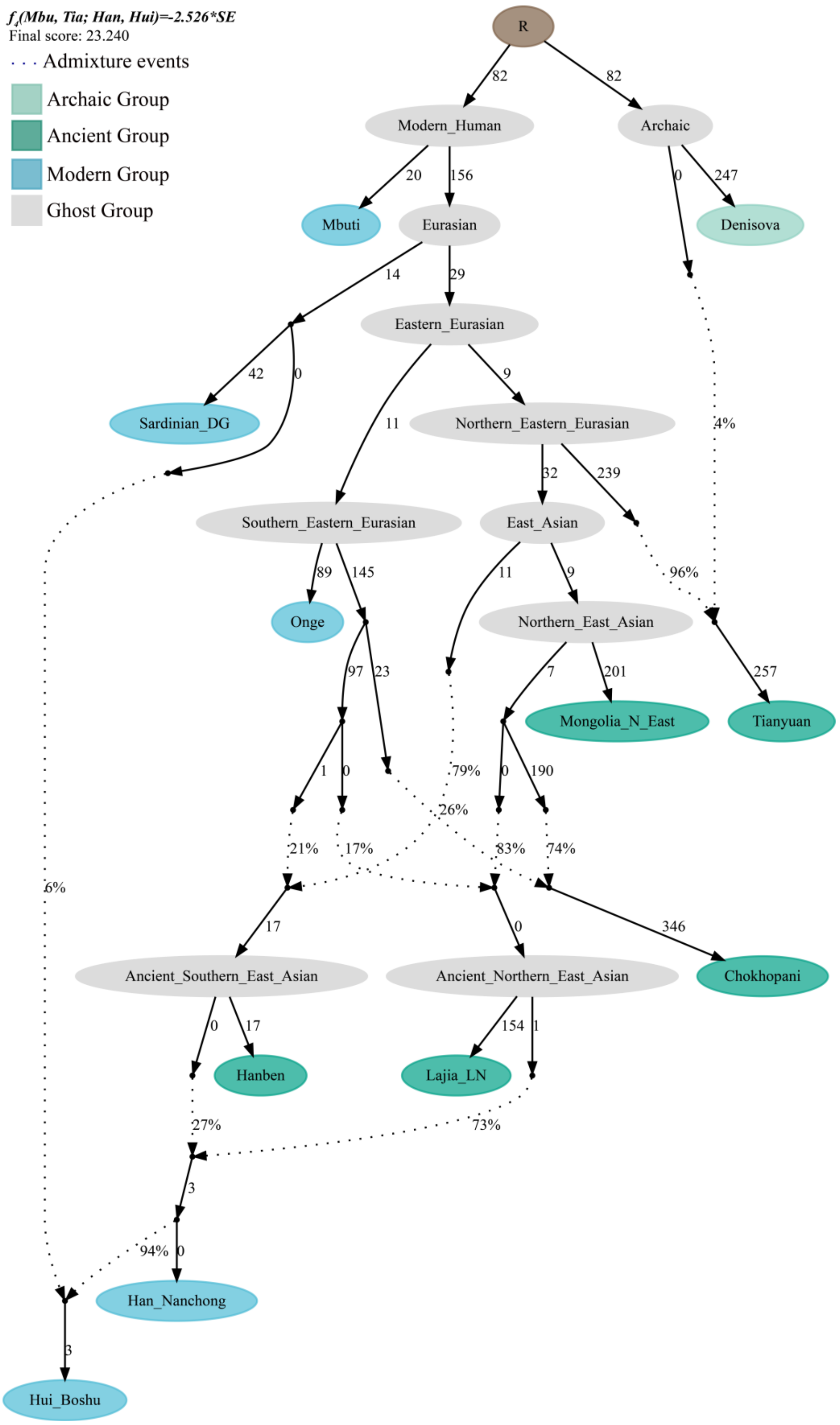

Figure S21. *qpGraph*-based admixture graph showed the western Eurasian gene flow event related to Sardinian into Boshu Hui.

Branch length was marked with the f_2 shared drift distance (1000 times). Admixture events were denoted as dotted line. Admixture proportion was marked along the dotted line. LN, Late Neolithic; N, Neolithic.

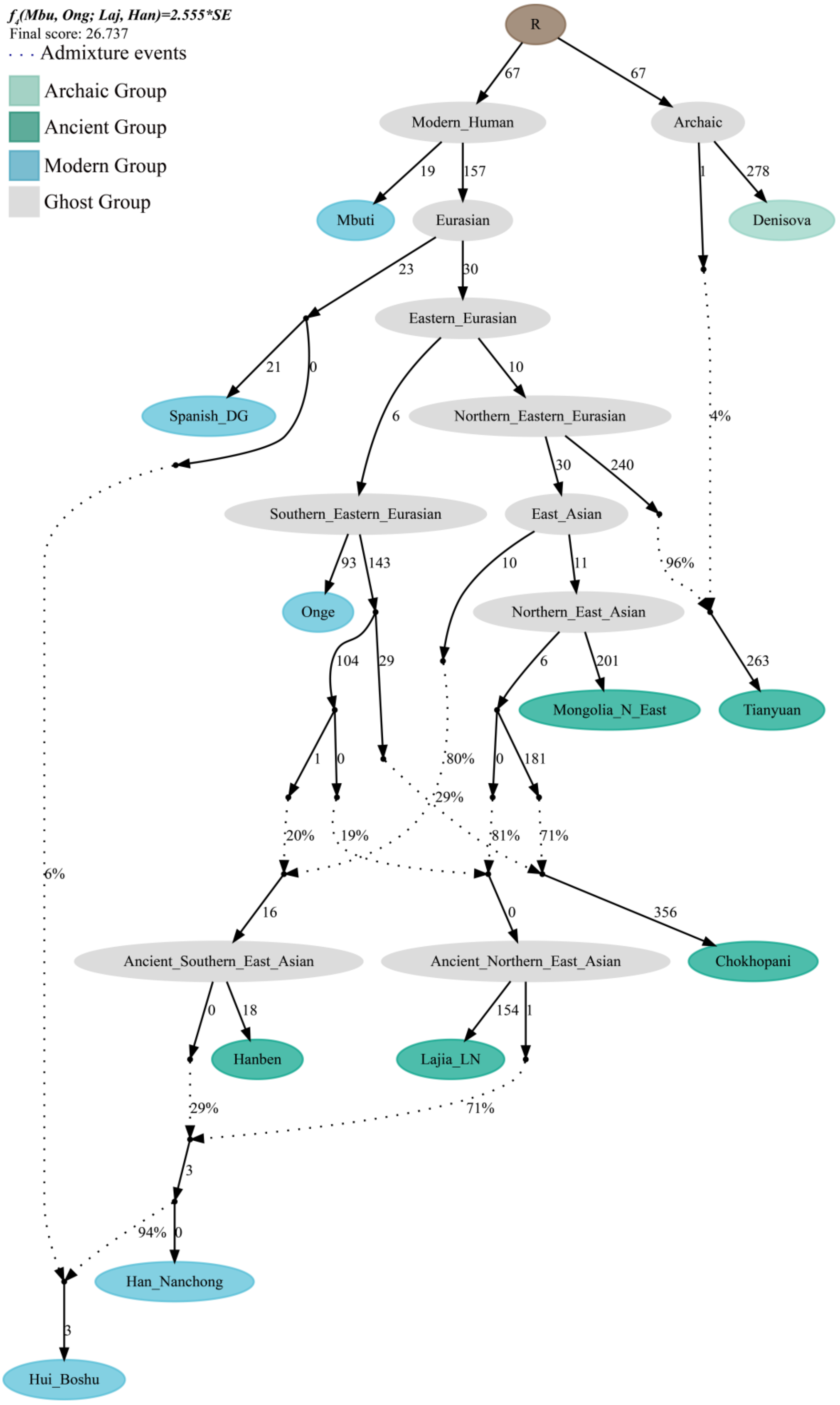

Figure S22. *qpGraph*-based admixture graph showed the western Eurasian gene flow event related to Spanish into Boshu Hui.

Branch length was marked with the f_2 shared drift distance (1000 times). Admixture events were denoted as dotted line. Admixture proportion was marked along the dotted line. LN, Late Neolithic; N, Neolithic.

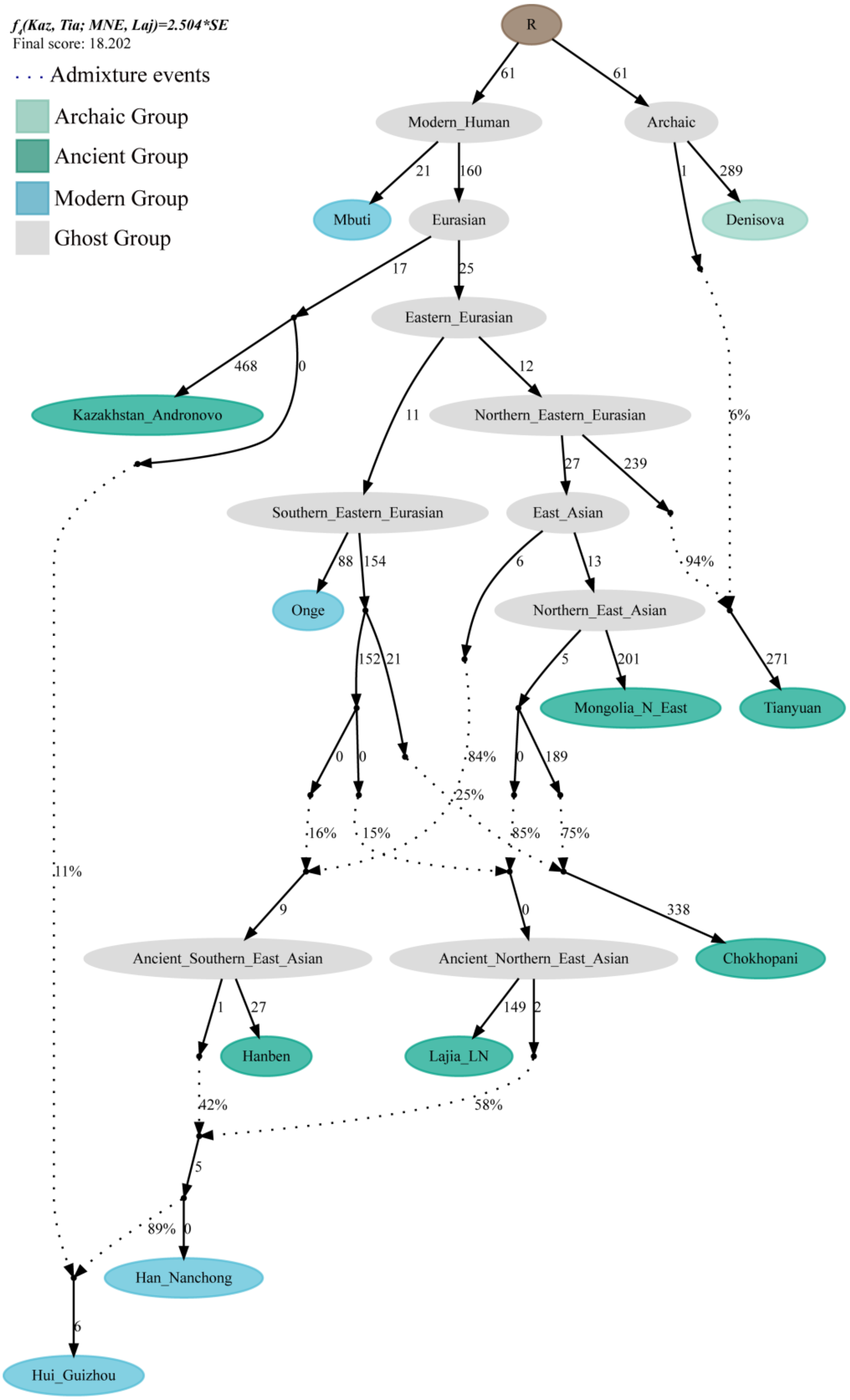

Figure S23. ***qpGraph*-based admixture graph showed the western Eurasian gene flow event related to Kazakhstan Andronovo into Guizhou Hui.**

Branch length was marked with the f_2 shared drift distance (1000 times). Admixture events were denoted as dotted line. Admixture proportion was marked along the dotted line. EBA: Early Bronze Age; LN, Late Neolithic; N, Neolithic.

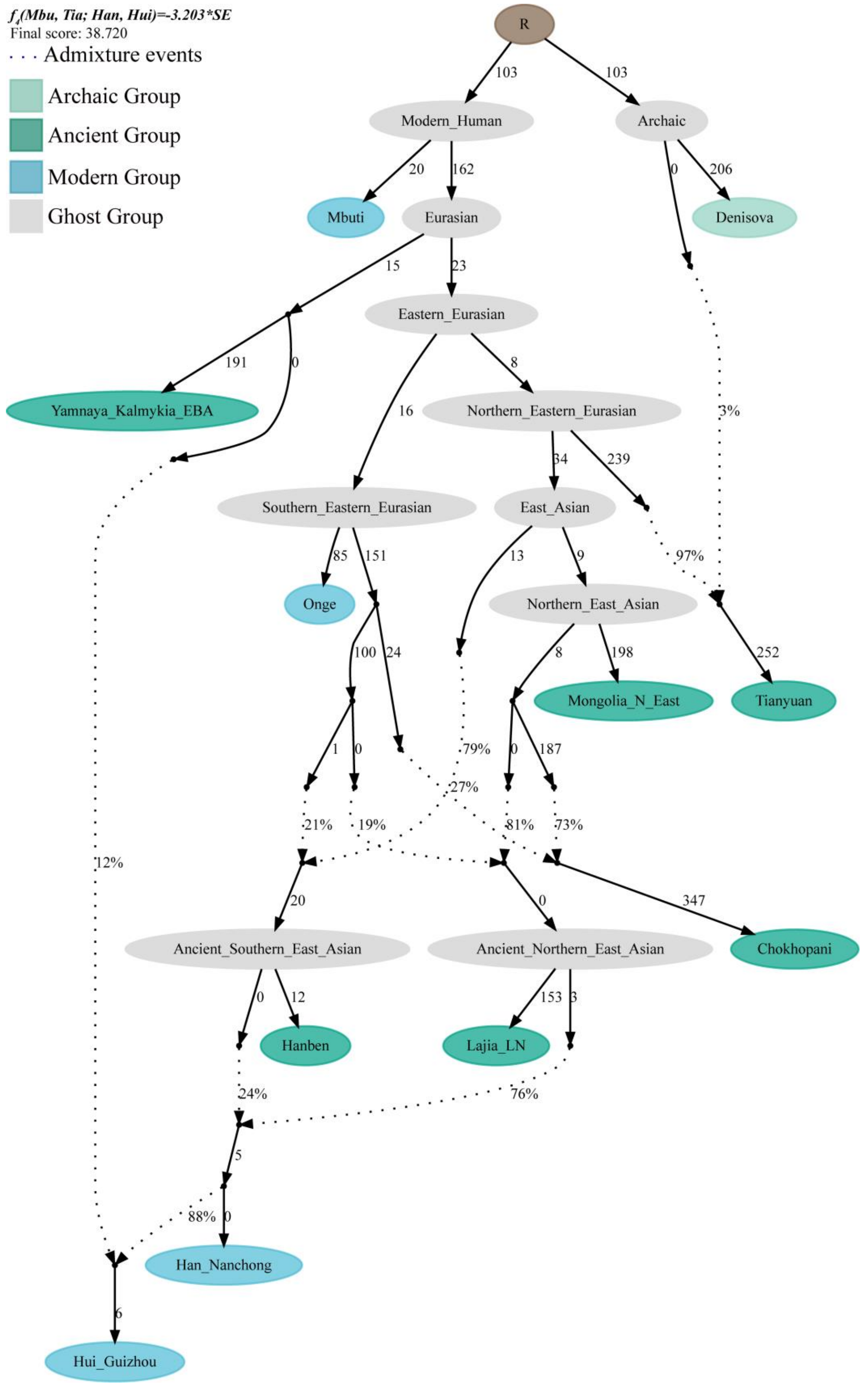

Figure S24. *qpGraph*-based admixture graph showed the western Eurasian gene flow event related to Kalmykia Yamnaya into Guizhou Hui.

Branch length was marked with the f_2 shared drift distance (1000 times). Admixture events were denoted as dotted line. Admixture proportion was marked along the dotted line. EBA: Early Bronze Age; LN, Late Neolithic; N, Neolithic.

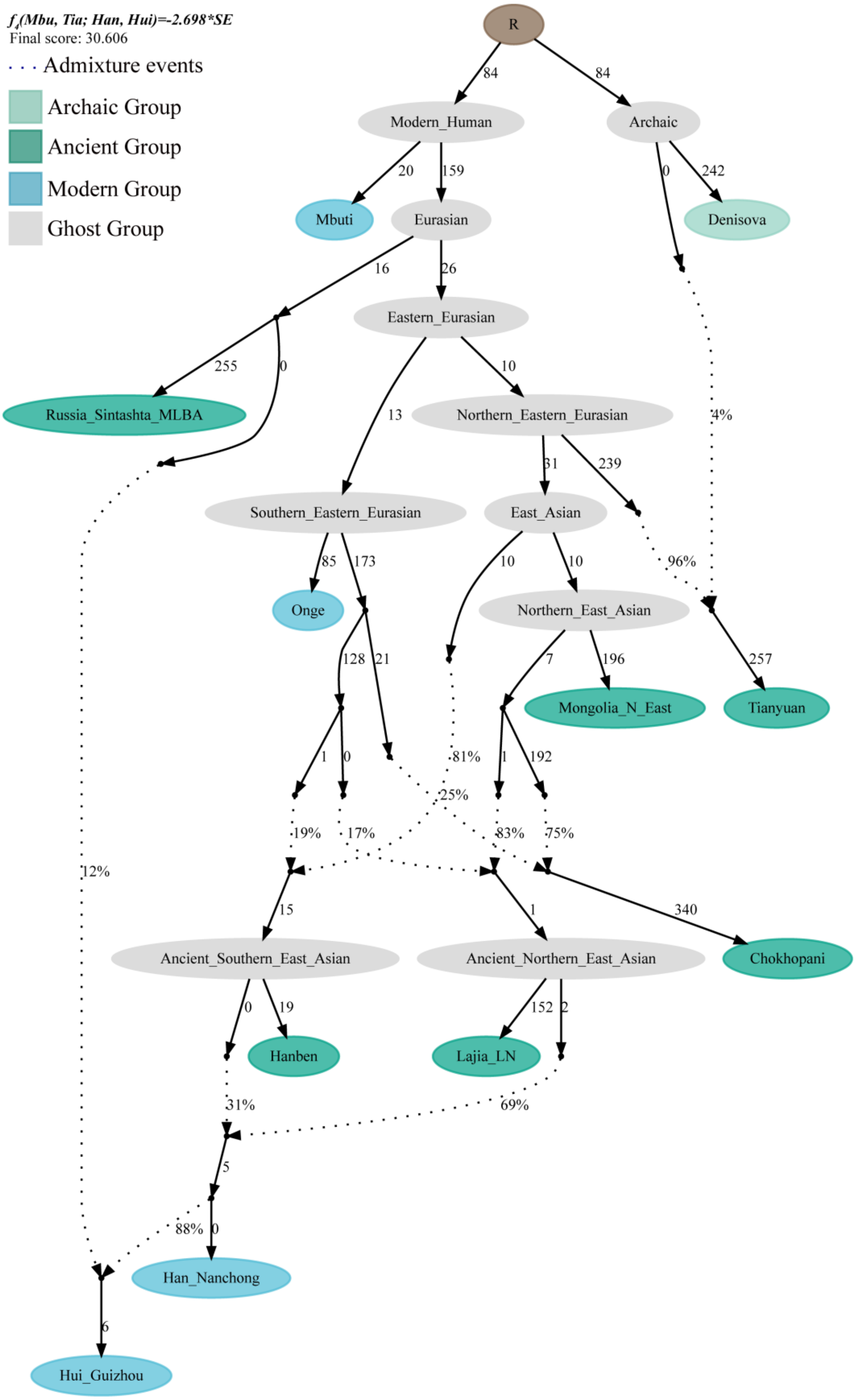

Figure S25. ***qpGraph*-based admixture graph showed the western Eurasian gene flow event related to Russia_Sintashta_MLBA into Guizhou Hui.**

Branch length was marked with the f_2 shared drift distance (1000 times). Admixture events were denoted as dotted line. Admixture proportion was marked along the dotted line. MLBA: Middle-Late Bronze Age;

LN, Late Neolithic; N, Neolithic.

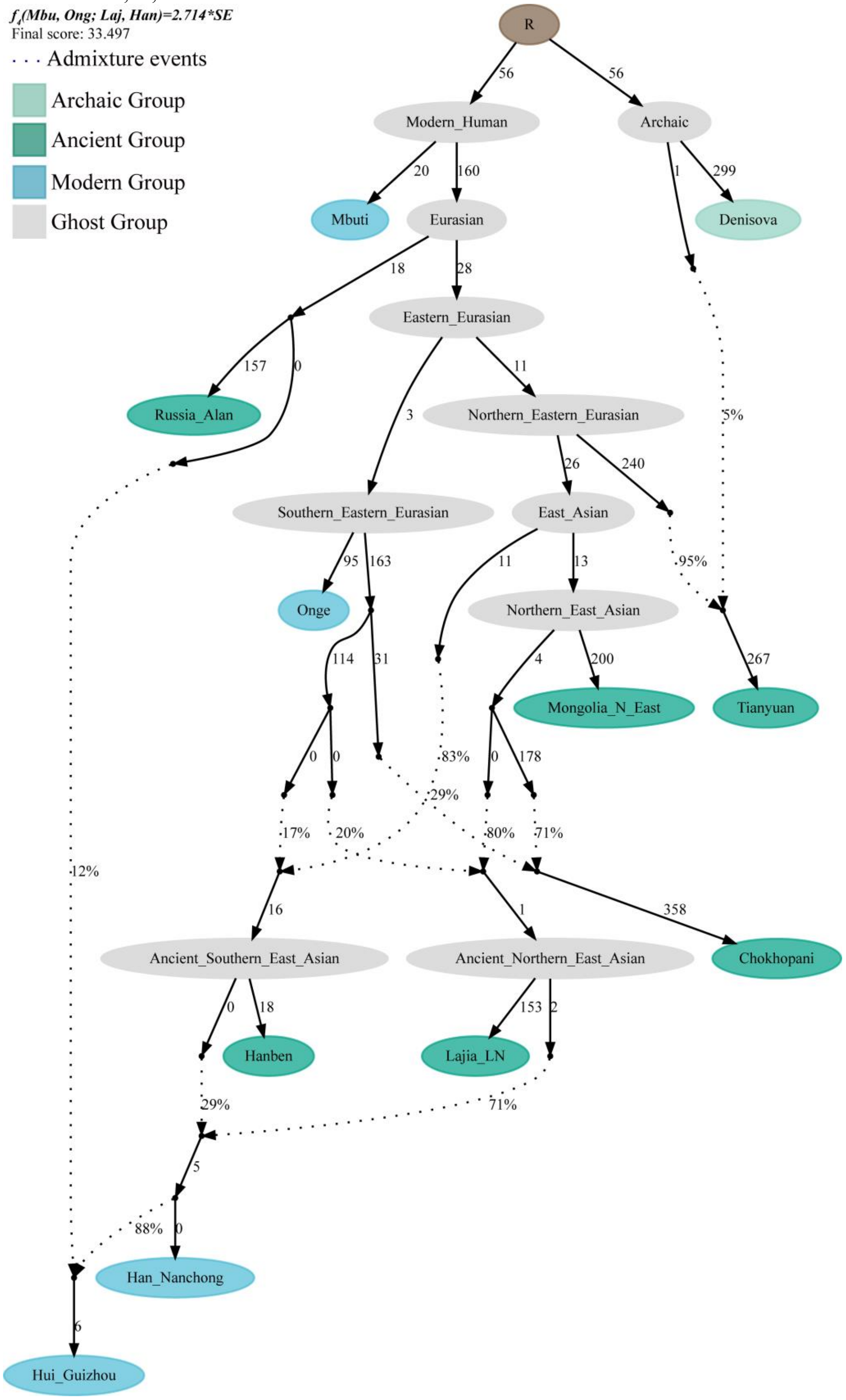

Figure S26. ***qpGraph*-based admixture graph showed the western Eurasian gene flow event related to Russia Alan into Guizhou Hui.**

Branch length was marked with the f_2 shared drift distance (1000 times). Admixture events were denoted as dotted line. Admixture proportion was marked along the dotted line. LN, Late Neolithic; N, Neolithic.

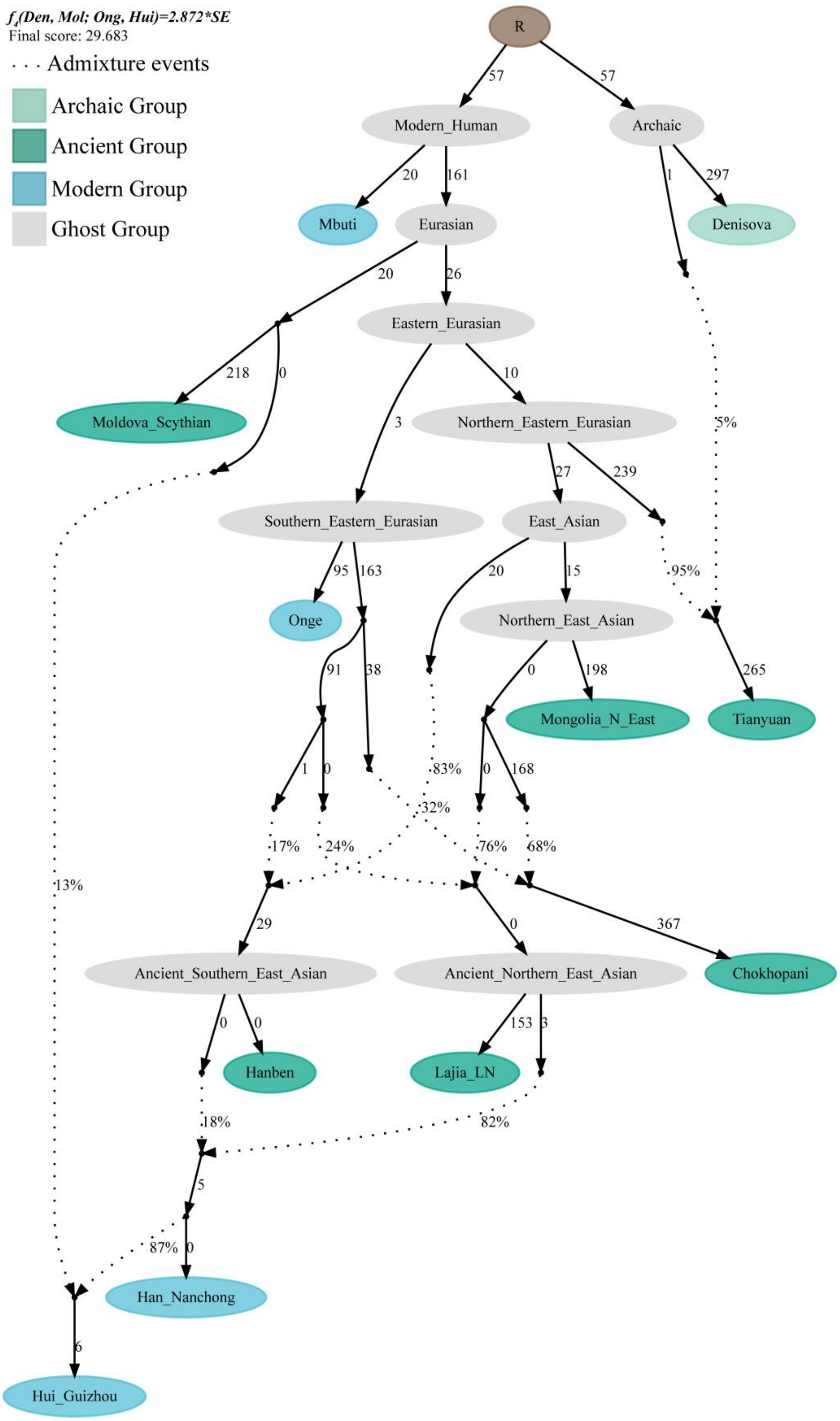

Figure S27. ***qpGraph*****-based admixture graph showed the western Eurasian gene flow event related to Moldova Scythian into Guizhou Hui.**

Branch length was marked with the f_2 shared drift distance (1000 times). Admixture events were denoted as dotted line. Admixture proportion was marked along the dotted line. LN, Late Neolithic; N, Neolithic.

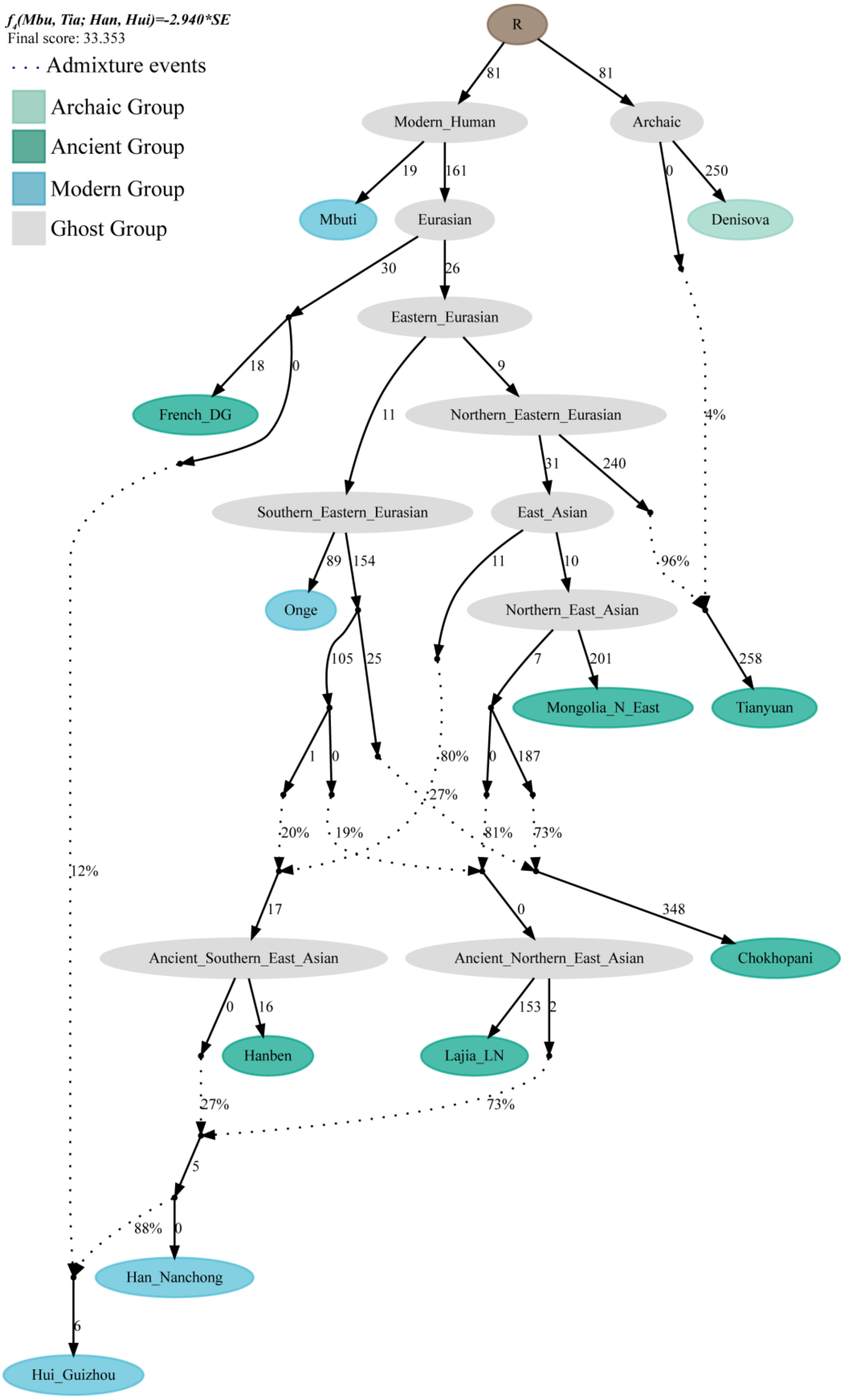

Figure S28. ***qpGraph*-based admixture graph showed the western Eurasian gene flow event related to French into Guizhou Hui.**

Branch length was marked with the f_2 shared drift distance (1000 times). Admixture events were denoted as dotted line. Admixture proportion was marked along the dotted line. LN, Late Neolithic; N, Neolithic.

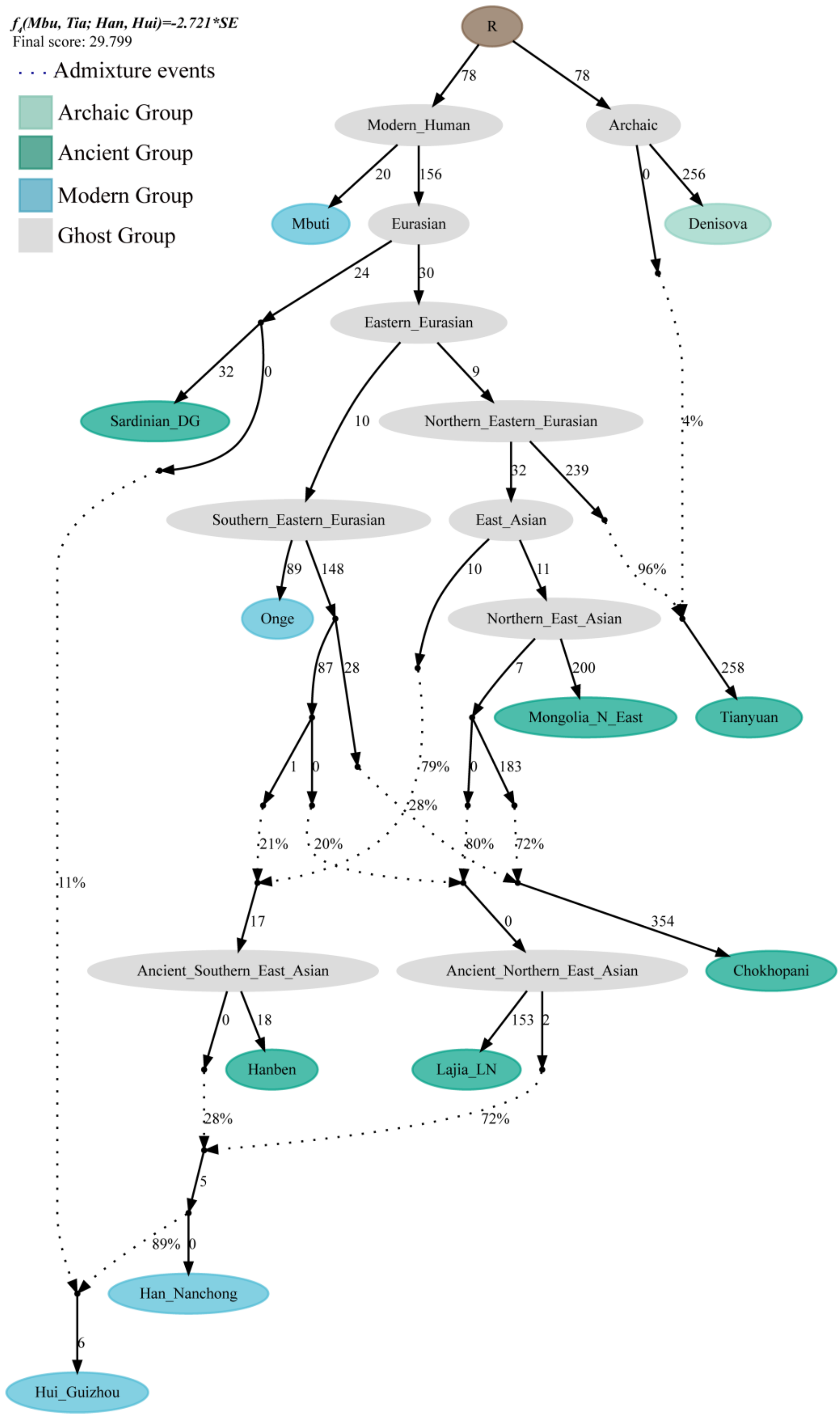

Figure S29. ***qpGraph*****-based admixture graph showed the western Eurasian gene flow event related to Sardinian into Guizhou Hui.**

Branch length was marked with the f_2 shared drift distance (1000 times). Admixture events were denoted as dotted line. Admixture proportion was marked along the dotted line. LN, Late Neolithic; N, Neolithic.

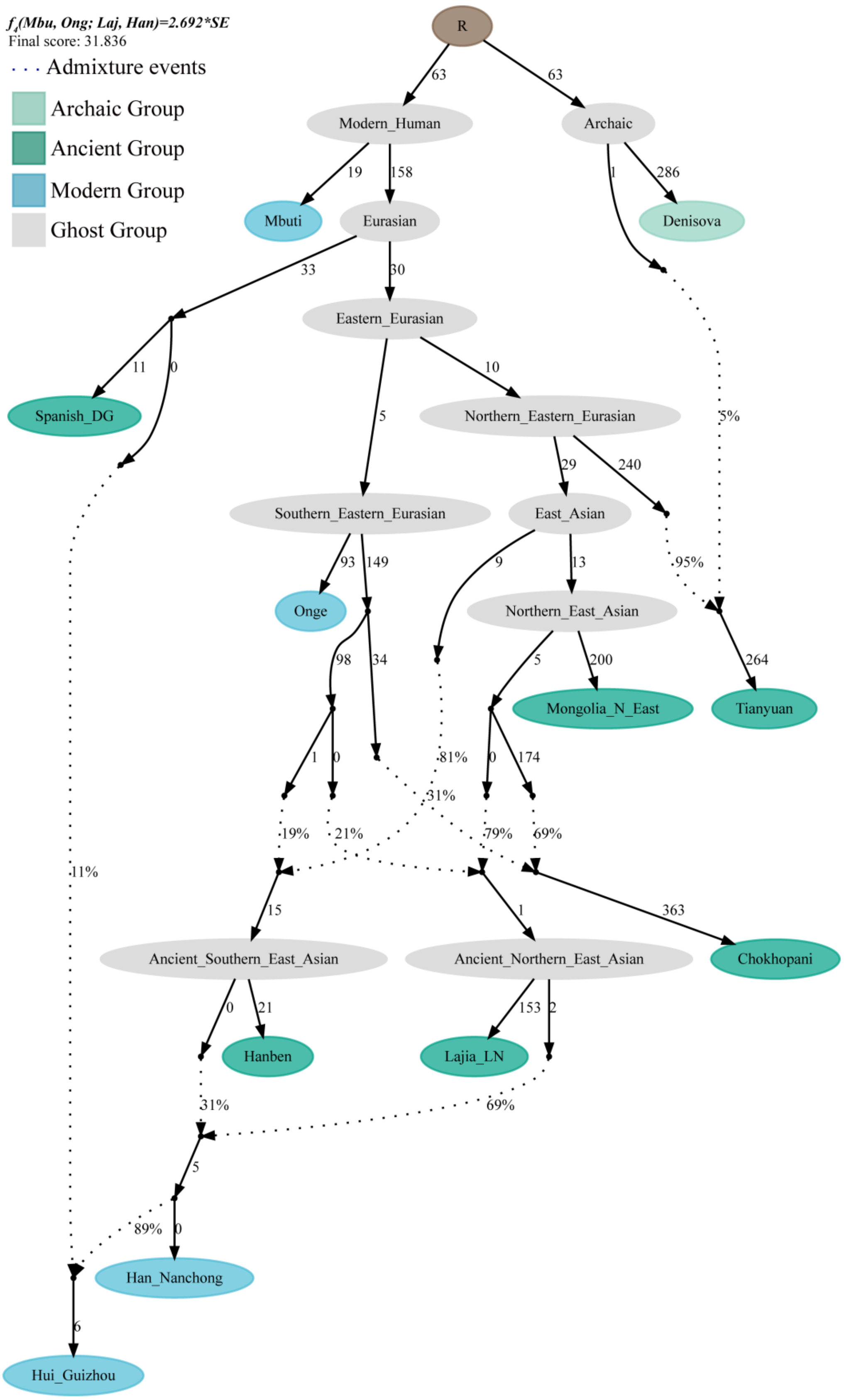

Figure S30. *qpGraph*-based admixture graph showed the western Eurasian gene flow event related to Spanish into Guizhou Hui.

Branch length was marked with the f_2 shared drift distance (1000 times). Admixture events were denoted as dotted line. Admixture proportion was marked along the dotted line. LN, Late Neolithic; N, Neolithic.

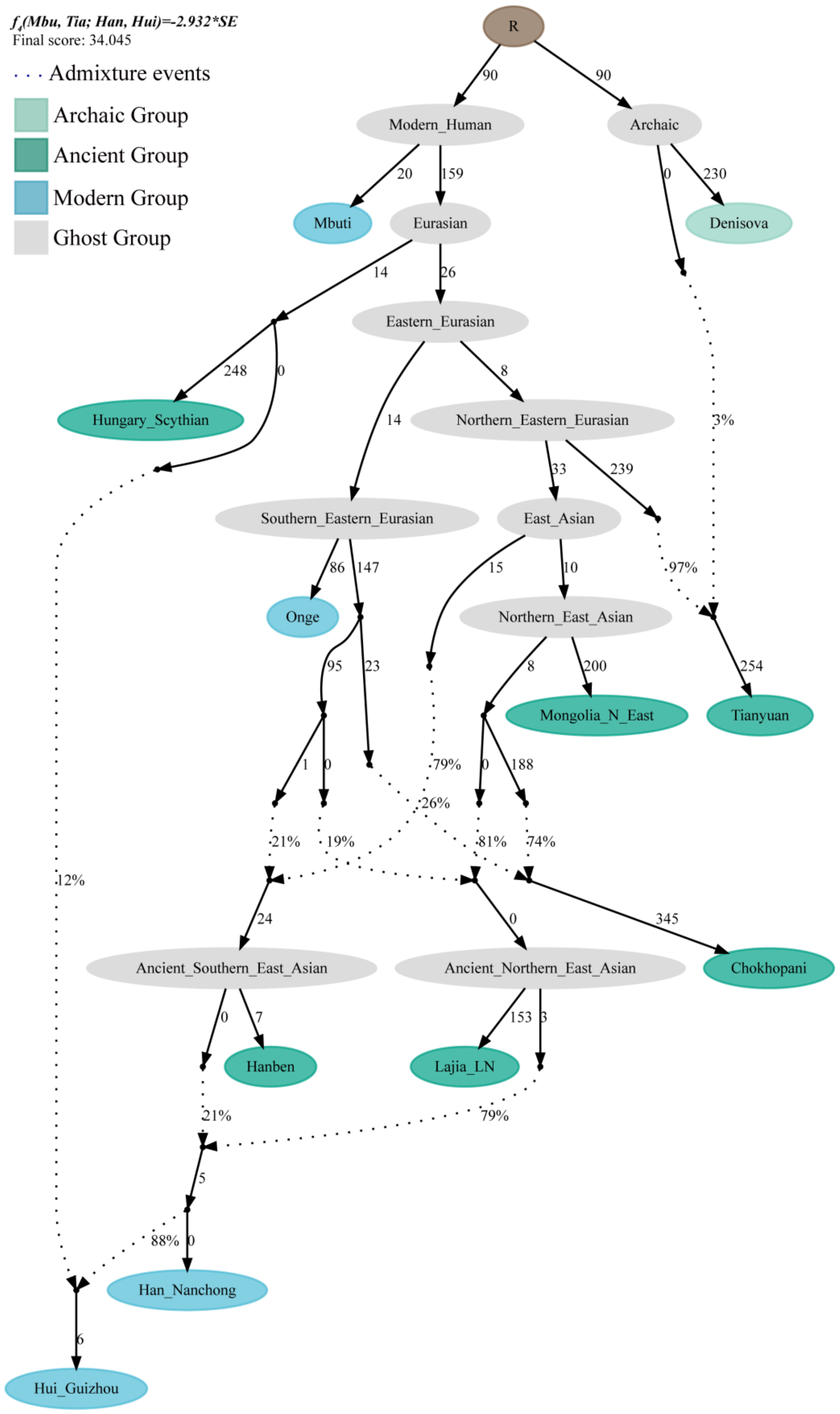

Figure S31. ***qpGraph*-based admixture graph showed the western Eurasian gene flow event related to Hungary Scythian into Guizhou Hui.**

Branch length was marked with the f_2 shared drift distance (1000 times). Admixture events were denoted as dotted line. Admixture proportion was marked along the dotted line. LN, Late Neolithic; N, Neolithic.

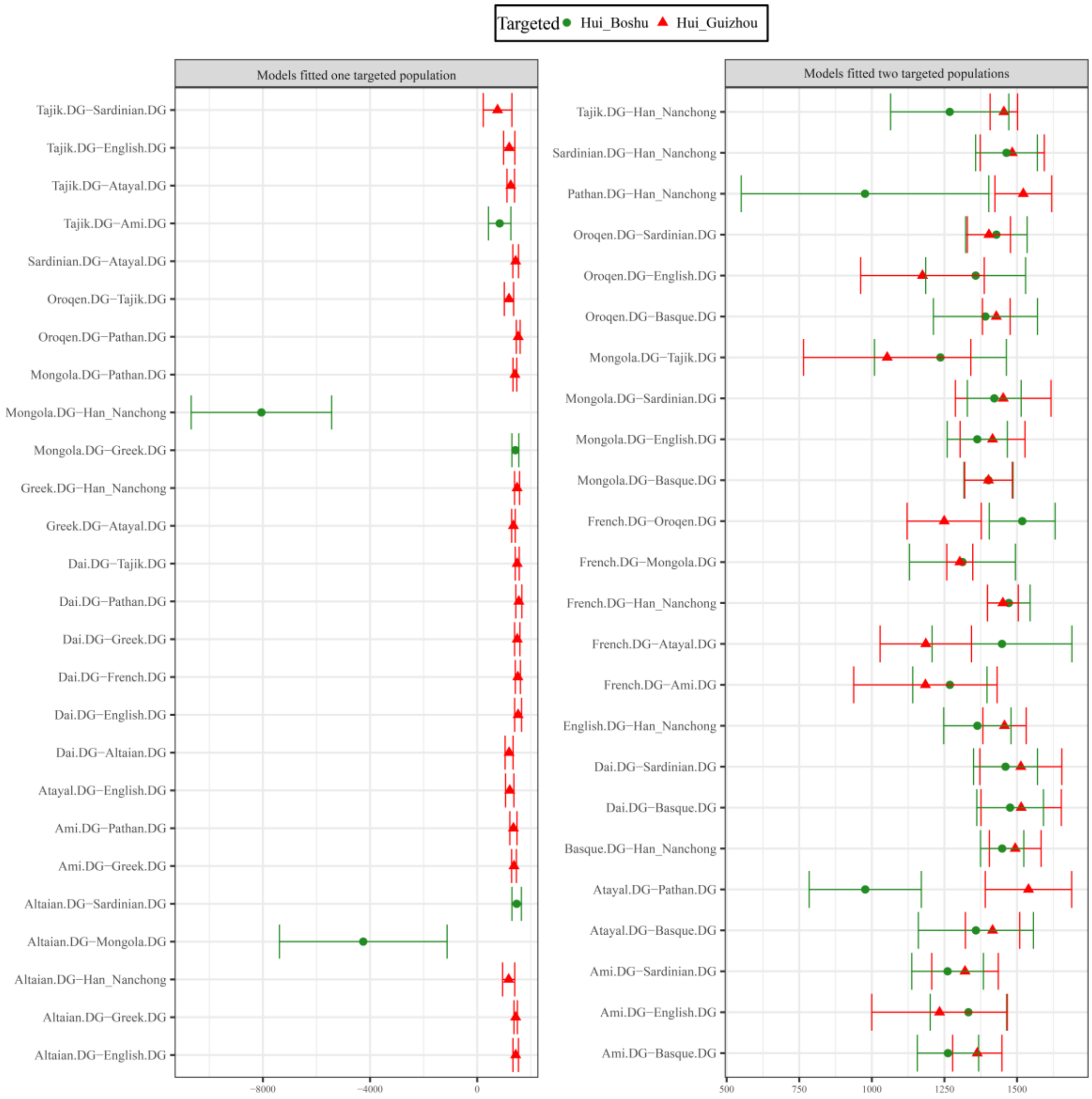

Figure S32. Admixture-introduced linkage disequilibrium (ALDER) based admixture time between different northern and southern or eastern and western ancestral sources based on 1240K dataset.

We used 28 years as the one generation length. All marked years in the bottom was calculated using the formula as Year=1950-28*(Generation-1). All comprehensive raw data were presented in Supplementary **Table S23**
